# A revision of the new genus *Amiga* Nakahara, Willmott & Espeland, gen. n., described for *Papilioarnaca* Fabricius, 1776 (Lepidoptera, Nymphalidae, Satyrinae)

**DOI:** 10.3897/zookeys.821.31782

**Published:** 2019-01-31

**Authors:** Shinichi Nakahara, Gerardo Lamas, Stephanie Tyler, Mario Alejandro Marín, Blanca Huertas, Keith R. Willmott, Olaf H. H. Mielke, Marianne Espeland

**Affiliations:** 1 McGuire Center for Lepidoptera and Biodiversity, Florida Museum of Natural History, University of Florida, Gainesville, Florida 32611 USA University of Florida Gainesville United States of America; 2 Museo de Historia Natural, Universidad Nacional Mayor de San Marcos, Lima, Peru Universidad Nacional Mayor de San Marcos Lima Peru; 3 School of Architecture, Rice University, 6100 Main Street, Houston, Texas 77005 USA Rice University Houston United States of America; 4 Departamento de Biologia Animal and Museu de Zoologia, Instituto de Biologia, Universidade Estadual de Campinas – UNICAMP. 13083-970 Campinas, São Paulo, Brazil Universidade Estadual de Campinas Campinas Brazil; 5 Life Sciences Department, Natural History Museum, Cromwell Road, London SW7 5BD, UK Natural History Museum London United Kingdom; 6 Laboratório de Estudos de Lepidoptera Neotropical, Departamento de Zoologia, Universidade Federal do Paraná, Caixa postal 19020, 81.531 Curitiba, Paraná, Brazil Universidade Federal do Paraná Curitiba Brazil; 7 Arthropoda Department, Zoological Research Museum Alexander Koenig, Adenauer Allee 160, 53113 Bonn, Germany Zoological Research Museum Alexander Koenig Bonn Germany

**Keywords:** DNA barcodes, Euptychiina, species delimitation, subspecies, systematics, taxonomy

## Abstract

We here propose a new, monotypic genus, *Amiga* Nakahara, Willmott & Espeland, **gen. n.**, to harbor a common Neotropical butterfly, described as *Papilioarnaca* Fabricius, 1776, and hitherto placed in the genus *Chloreuptychia* Forster, 1964. Recent and ongoing molecular phylogenetic research has shown *Chloreuptychia* to be polyphyletic, with *C.arnaca* proving to be unrelated to remaining species and not readily placed in any other described genus. *Amigaarnaca***gen. n. et comb. n.** as treated here is a widely distributed and very common species ranging from southern Mexico to southern Brazil. A neotype is designated for the names *Papilioarnaca* and its junior synonym, *Papilioebusa* Cramer, 1780, resulting in the treatment of the latter name as a junior objective synonym of the former. A lectotype is designated for *Euptychiasericeella* Bates, 1865, which is treated as a subspecies, *Amigaarnacasericeella* (Bates, 1865), **comb. n. et stat. n.**, based on molecular and morphological evidence. We also describe two new taxa, *Amigaarnacaadela* Nakahara & Espeland, **ssp. n.** and *Amigaarnacaindianacristoi* Nakahara & Marín, **ssp. n.**, new subspecies from the western Andes and eastern Central America, and northern Venezuela, respectively.

## Introduction

Butterflies are considered to have the best-studied taxonomy of any insect group, but the nymphalid subfamily Satyrinae includes some of the remaining groups still in most need of research, in part owing to its high diversity. Many new satyrine taxa have recently been discovered and described, including some that are among the most common elements of the butterfly fauna around us (e.g., Cong and Grishin 2014; Freitas et al. 2016).

*Chloreuptychia* Forster, 1964 is a genus in the Satyrinae subtribe Euptychiina, currently (Lamas 2004) containing some of the most brightly coloured euptychiine species, which possess a bluish-lilac reflection on their wings. Butler (1867) made one of the first attempts to classify those species later placed in *Chloreuptychia* by Forster (1964) in a systematic context. The “Division IV” proposed by Butler (1867) was distinguished from other closely related species by “Wings dorsally fuscous, mostly with a violet sheen; ventrally with ocellated spots, those of the hindwings often with their centers elongated and silver”, and he further separated this “Division IV” into two subdivisions. The “subdivision 1” was characterized by “Wings ventrally with regular-shaped ocelli, not elongated” and included *Euptychiasericeella* Bates, 1865 and *Papilioebusa* Cramer, 1780, “subdivision 2” was characterized by “Hindwings ventrally with ocelli centered with irregular and elongated spots” and included *P.chloris* Cramer, 1780, *P.herse* Cramer, 1775, *E.callichloris* Butler, 1867, *E.hewitsonii* Butler, 1867, *E.agatha* Butler, 1867, and *E.tolumnia* Cramer, 1777. Both subdivisions also contained some species currently (Lamas 2004) not placed in *Chloreuptychia*. Subsequently, Butler (1877) grouped species related to species currently placed in *Chloreuptychia* under his “*arnaea* [sic] group”, although without providing a diagnosis, including *Euptychiaarnaea* [sic] Fabricius, 1776, *E.sericeella*, *E.chloris*, *E.herse*, *E.callichloris*, *E.hewitsonii*, *E.agatha*, and *E.tolumnia*, in addition to three unrelated species. Unlike in Butler (1867), *Euptychiaarnaea* [sic] and *E.sericeella* were apparently considered as conspecific in Butler (1877). Weymer (1911) recognized *E.arnaea* [sic], *E.sericella* [sic], *E.chloris*, *E.marica* Weymer, 1911, *E.herse*, and *E.callichloris* in his “Arnaea [sic] group”, and *E.tolumnia*, *E.catharina* Staudinger, [1886], and *E.hewitsonii* in his “Tolumnia group”, in which the latter group was characterized by the presence of forewing androconial scales (scent-scales) in males. Forster (1964) erected the genus *Chloreuptychia* by designating *P.chloris* as the type species of the genus, and recognized *E.arnaea* [sic], *E.sericella* [sic], *P.chloris*, *E.herse*, *E.marica*, *E.catharina*, *E.tolumnia*, and *E.hewitsonii* in his new genus. This classification was followed by Miller (1968). Lamas (2004) recognized 10 species in *Chloreuptychia*, namely *C.agatha*, *C.arnaca*, *C.callichloris*, *C.catharina*, *C.chlorimene* (Hübner, [1819]), *C.herseis* (Godart, [1824]), *C.hewitsonii*, *C.marica*, *C.sericeella*, and *C.tolumnia*, in addition to two undescribed species from Peru. Importantly, Lamas (2004) listed replacement names provided by subsequent authors for the preoccupied names *P.herse* and *P.chloris*, namely *C.herseis* and *P.chlorimene* respectively, both of which were ignored in earlier studies. Recently, *C.amethysta* Brévignon & Benmesbah, 2012 was described as a new species, bringing the total number of described species for this genus to eleven (Lamas 2004; Brévignon and Benmesbah 2012).

Previous authors apparently placed species in *Chloreuptychia* based mainly on the bluish-lilac reflection on the wing surface, without testing monophyly and/or synapomorphies, and the genus has proved to be highly polyphyletic based on recent broad collaborative research on the group (Espeland et al. 2019; unpubl. data). In order to contribute towards a better understanding of the Euptychiina systematics, we here describe a new genus to harbor *P.arnaca*, and review the taxonomy of this common component of the butterfly community in the Neotropics. Neotypes for the names *P.arnaca* and *P.ebusa* are designated, and two new subspecies are described based on wing pattern and DNA data.

## Material and methods

### Morphological study

We studied external morphology by soaking legs, labial palpi, and abdomens in hot 10% KOH solution for 5–10 minutes and dissecting them, storing them in glycerine after examination. Membranous structures of the phallus and female genitalia were stained with chlorazol black prior to examination to better visualize membranous structures. Drawings of external morphology were done using a camera lucida attached to a Leica MZ 16 stereomicroscope at FLMNH. Terminology for wing venation and wing pattern elements follows Nakahara et al. (2018b); nomenclature of genitalia follows Nakahara et al. (2018a).

The following collection acronyms are used throughout the text:

**BME** Bohart Museum of Entomology, University of California, Davis, CA, USA

**CBF** Colección Boliviana de Fauna, La Paz, Bolivia

**CMNH** Carnegie Museum of Natural History, Pittsburgh, USA

**DZUP** Departamento de Zoologia, Universidade Federal do Paraná, Curitiba, Brazil

**FLMNH** McGuire Center for Lepidoptera and Biodiversity, Florida Museum of Natural History, University of Florida, Gainesville, USA

**FRPI** Francisco Piñas collection, Quito, Ecuador

**HERENCIA** Corporación Herencia Natural y Cultural collection, Florencia, Colombia

**ICNA** Ichiro Nakamura collection, Williamsville, USA

**INABIO** Instituto Nacional de Biodiversidad, Ecuador (formerly MECN)

**LBCB** L. & C. Brévignon collection, Cayenne, French Guiana

**MEFLG** Museo Entomológico ‘Francisco Luis Gallego’, Medellín, Colombia

**MHNNKM** Museo de Historia Natural ‘Noel Kempff Mercado’, Santa Cruz, Bolivia

**MNHU** Museum für Naturkunde, Leibniz-Institut für Evolutions- und Biodiversitätsforschung an der Humboldt Universität, Berlin, Germany (formerly ZMHU)

**MUA** Museo Universitario, Universidad de Antioquia, Medellín, Colombia

**MUSM** Museo de Historia Natural, Universidad Nacional Mayor de San Marcos, Lima, Peru

**NHMUK** Natural History Museum, London, UK (formerly BMNH)

**PUCE** Museo de Entomología, Pontificia Universidad Católica del Ecuador, Quito, Ecuador

**RMNH** Rijksmuseum van Natuurlijke Historie (presently Netherlands Centre for Biodiversity Naturalis), Leiden, Netherlands

**UMG** Hunterian Museum, University Museum of Glasgow, Glasgow, UK

**USNM** National Museum of Natural History, Smithsonian Institution, Washington, DC, USA

**ZSM** Zoologische Staatssammlung München, Munich, Germany

**ZUEC** Museu de Zoologia da Universidade Estadual de Campinas ‘Adão José Cardoso’, Campinas, Brazil

### Phylogeny and species delimitation

DNA was extracted from leg or thorax tissue, either dried or stored in 96% ethanol. Voucher specimens are deposited at FLMNH, DZUP and ZUEC.

DNA extraction methods, PCR conditions and primers used for amplification of cytochrome c oxidase I (COI), elongation factor 1-alpha (EF1-a), glyceraldehyde 3-phosphate dehydrogenase (GAPDH) and ribosomal protein S5 (RPS5) follow Nakahara et al. (2015, 2018a, 2018b). Sequences for additional specimens were taken from Peña et al. (2010), Matos-Maraví et al. (2013) and Espeland et al. (2019) (Table 1). The complete concatenated Sanger dataset contained 19 taxa, including eight *A.arnaca* specimens, and 2934bps. As it has previously been shown that using the standard genes above might not provide sufficient support for the deeper relationships of Euptychiina (Peña et al. 2010), we also inferred a phylogeny using the 368 loci (182, 350bps) for 17 of the taxa analyzed by Espeland et al. (2019). This dataset contains single specimens of *Amiga* gen. n., *Chloreuptychiachlorimene*, *C.herseis*, and *C.catharina* as well as outgroups to better show the placement of *arnaca* relative to other members of *Chloreuptychia*. These are available on Mendeley Data (https://doi.org/10.17632/m7gc59vnp3.1).

**Table 1.** GenBank accession numbers for specimens used for molecular analysis in this study.

**Voucher code**	**Genus**	**Species/ Subspecies**	**Genes**			
			**COI**	**EF1a**	**GAPDH**	**RPS5**
LEP-16938	* Pseudodebis *	valentina	SAMN09745417	SAMN09745417	SAMN09745417	SAMN09745417
LEP-10646	* Taygetis *	cleopatra	MK305304	N/A	N/A	N/A
BC-DZ-250	* Archeuptychia *	cluena	SAMN09745463	SAMN09745463	SAMN09745463	SAMN09745463
LEP-19580	* Chloreuptychia *	* herseis *	SAMN09745427	N/A	N/A	N/A
LEP-14945		* herseis *	MK279723	N/A	N/A	N/A
KW 081111 51		* herseis *	MK279714	N/A	N/A	N/A
LEP-04394		* herseis *	MK279719	MK305298	MK305291	MK305285
LEP-09835	* Euptychoides *	* eugenia *	SAMN09745408	SAMN09745408	SAMN09745408	SAMN09745408
LEP-10685		* eugenia *	MK305305	N/A	N/A	N/A
LEP-10059		* eugenia *	MK305306	N/A	N/A	N/A
YPH0575	* Nhambikuara *	cerradensis	MF489987	N/A	N/A	N/A
NW149-11	* Taydebis *	peculiaris	GQ864811	GQ864905	GQ865036	GQ865499
CP06 70	* Megeuptychia *	monopunctata	GU205852	GU205908	GU205964	GU206024
LEP-16939	* Magneuptychia *	philippa	SAMN09745418	SAMN09745418	SAMN09745418	SAMN09745418
LEP-10710	* Chloreuptychia *	* agatha *	MK305307	N/A	N/A	N/A
LEP-04406		* agatha *	N/A	MK305299	MK305292	MK305286
KW 140708-02		* chlorimene *	SAMN09745398	SAMN09745398	SAMN09745398	SAMN09745398
KW-140705-07		* agatha *	MK305308	N/A	N/A	N/A
02-SRNP-5948	* Amiga *	* arnaca adela *	ADE52482	N/A	N/A	N/A
07-SRNP-100105		* arnaca adela *	AFA15201	N/A	N/A	N/A
BC-DZ_Willmott-055		* arnaca arnaca *	MK291952	N/A	N/A	N/A
BC-DZ_Willmott-056		* arnaca arnaca *	MK291953	N/A	N/A	N/A
BC-DZ_Willmott-057		* arnaca arnaca *	MK291954	N/A	N/A	N/A
CP06-76		* arnaca arnaca *	GU205829	GU205885	GU205941	GU206001
DNA99-015		* arnaca arnaca *	AY508527	AY509054	N/A	N/A
LEP-04408		* arnaca arnaca *	MK291955	N/A	N/A	N/A
LEP-09788		* arnaca adela *	MK291956	N/A	MK305293	MK305287
LEP-09930		* arnaca adela *	MK291957	MK305300	MK305294	MK305288
LEP-09931		* arnaca adela *	MK291958	N/A	N/A	N/A
LEP-10696		* arnaca arnaca *	MK291959	MK305301	MK305295	
LEP-10697		* arnaca arnaca *	MK291960	N/A	N/A	N/A
LEP-10703		* arnaca arnaca *	MK291961	MK305302	MK305296	MK305289
LEP-15082		* arnaca arnaca *	MK291962	N/A	N/A	N/A
LEP-16997		* arnaca sericeella *	MK291963	MK305303	MK305297	MK305290
LEP-34357		* arnaca arnaca *	MK291964	N/A	N/A	N/A
LEP-37404		* arnaca arnaca *	MK291965	N/A	N/A	N/A
LEP-37411		* arnaca arnaca *	MK291966	N/A	N/A	N/A
LEP-37416		* arnaca arnaca *	MK291967	N/A	N/A	N/A
LEP-37525		* arnaca arnaca *	MK291968	N/A	N/A	N/A
LEP-55465		* arnaca arnaca *	MK291969	N/A	N/A	N/A
MGCL-LOAN-028		* arnaca arnaca *	MK291970	N/A	N/A	N/A
MGCL-LOAN-090		* arnaca arnaca *	MK291971	N/A	N/A	N/A
MGCL-LOAN-139		* arnaca arnaca *	MK291972	N/A	N/A	N/A
MGCL-LOAN-144		* arnaca arnaca *	MK291973	N/A	N/A	N/A
MGCL-LOAN-162		* arnaca arnaca *	MK291974	N/A	N/A	N/A
MGCL-LOAN-217		* arnaca arnaca *	MK291975	N/A	N/A	N/A
YB-BCI23691		* arnaca arnaca *	KP848781	N/A	N/A	N/A
YB-BCI35406		* arnaca arnaca *	AKN57330	N/A	N/A	N/A
YB-BCI35436		* arnaca arnaca *	AKN57333	N/A	N/A	N/A
YB-BCI46591		* arnaca arnaca *	AKN57332	N/A	N/A	N/A
YB-BCI46628		* arnaca arnaca *	AKN57334	N/A	N/A	N/A
YB-BCI49395		* arnaca arnaca *	AKN57335	N/A	N/A	N/A
YB-BCI6807		* arnaca arnaca *	ADK42359	N/A	N/A	N/A
YB-BCI766		* arnaca arnaca *	ADK42362	N/A	N/A	N/A
KW 140618-01		* arnaca arnaca *	SAMN09745390	SAMN09745390	SAMN09745390	SAMN09745390

Sequences generated by Sanger sequencing were assembled using Geneious 10 (Biomatters), and aligned using MAFFT v. 7 (Katoh 2013). Phylogenies were inferred for each gene separately (Sanger data only) as well for the concatenated data. The genes were partitioned to codon position and partitions and models were selected using ModelFinder (Kalyaanamoorthy et al. 2017) in IQ-Tree 1.6.7 (Nguyen et al. 2015). Thereafter 200 tree searches were performed in IQ-tree and the tree with the highest likelihood was selected. Support was calculated based on 2000 ultrafast bootstrap replicates with the -*bnni* option to reduce the risk of overestimating branch support (Hoang et al. 2017). The trees were rooted with *Cyllopsishedemanni* R. Felder, 1869 based on prior information (Espeland et al. 2019). The hybrid enrichment data were cleaned, assembled and aligned according to Espeland et al. (2018, 2019). A phylogeny was inferred using IQ-tree as above, but with 1000 ultrafast bootstrap replicates, also rooting with *Cyllopsishedemanni*. All nodes with a support lower than 75 were collapsed and this tree was subsequently used as a constraint tree for the concatenated Sanger data, which was analyzed in IQ-Tree as above, leading to the final dataset containing 31 taxa. GenBank accession numbers for sequences used in this study can be found in Table 1.

For species delimitation, a dataset consisting only of COI sequences with unique haplotypes was used. Sequences were aligned as above. The alignment was shortened to minimize the amount of missing data at both ends and the final dataset consisted of 30 sequences with a length of 615 bps. This included 16 *Amigaarnaca* comb. n. specimens and multiple outgroups. Sequences were not available for one of the proposed taxa (*A.arnacaindianacristoi* ssp. n.). Single threshold GMYC (Generalized Mixed Yule Coalescent; Pons et al. 2006; Fujisawa and Barraclough 2013), bPTP (bayesian Poisson Tree Processes; Zhang et al. 2013) and ABGD (Automated Barcode Gap Discovery; Puillandre et al. 2012) were used to assess the threshold between infra- and interspecific relationships in *A.arnaca* comb. n. GMYC assigns branching events to either a yule process (interspecific) or the coalescent (intraspecific). The reduced COI dataset was partitioned into codon positions, and model selection and phylogenetic inference was done as above. The resulting tree was rendered ultrametric using semi-parametric penalized likelihood (Sanderson 2002) by applying the *chronos* function in the APE package (Paradis et al. 2004, Popescu et al. 2012) in R. The fit of four clock models (strict clock, discrete clock with 10 rate categories, correlated clock and relaxed clock) was tested using the Φ information criterion by Paradis (2013). For all four clock models three different values (0.01, 0.1, 1) of the smoothing parameter (lambda) were tested. A strict clock model was found to be the best fit and the smoothing parameter did not affect the species delimitation result, so only GMYC results for the strict clock tree with lambda = 1 are shown below. Support values of species clusters delimited by GMYC were calculated using information-theoretic multimodel inference (Fujisawa and Barraclough 2013). GMYC was performed using the SPLITS package v. 1.0-19 (from http://r-forge.r-project. org/projects/splits) in R. bPTP models branching events were based on the number of substitutions (Zhang et al. 2013), and consequently do not require an ultrametric input tree. We used the ML tree inferred above as input for bPTP analyses on the bPTP webserver (https://species.h-its.org/ptp/). The Markov Chain Monte Carlo (MCMC) was run for a total of 500,000 generations with thinning set to 1000 and burnin to 0.1. ABGD tries to find a barcode gap in the distribution of pair wise differences (Puillandre et al. 2012) and does not require an input tree. The reduced COI alignment was used as input for ABGD analyses on the ABGD webserver (http://wwwabi.snv.jussieu.fr/public/abgd/abgdweb.html) using the Kimura distance with transition/transvertion ratio set to 2. Pmin, Pmax, Steps and Nb bins were kept as default, and the relative gap width (X) was set to 1. The genetic distances were calculated based on the Tamura-Nei model using Geneious version 11.1.5 (Biomatters Ltd.) based on COI data (Table 2).

**Table 2.** Genetic distances calculated based on the Tamura-Nei model.

		**1**	**2**	**3**	**4**	**5**	**6**	**7**	**8**	**9**	**10**	**11**	**12**	**13**	**14**	**15**	**16**	**17**	**18**	**19**	**20**	**21**	**22**	**23**	**24**	**25**	**26**	**27**	**28**	**29**	**30**	**31**	**32**	**33**	**34**	**35**	**36**
**1**	02-SRNP-5948 Amigaarnacaadela (Costa Rica)		0.003	0.076	0.073	0.067	0.061	0.064	0.058	0.024	0.024	0.024	0.058	0.061	0.058	0.058	0.044	0.073	0.064	0.064	0.064	0.024	0.024	0.049	0.073	0.03	0.03	0.067	0.067	0.033	0.033	0.03	0.033	0.033	0.034	0.03	0.033
**2**	07-SRNP-100105 Amigaarnacaadela (Costa Rica)	0.003		0.063	0.065	0.058	0.058	0.074	0.058	0.026	0.026	0.027	0.057	0.06	0.056	0.062	0.043	0.061	0.056	0.059	0.056	0.026	0.026	0.049	0.062	0.029	0.029	0.059	0.061	0.028	0.028	0.027	0.028	0.028	0.03	0.026	0.028
**3**	BC-DZ_Willmott-055 Amigaarnacaarnaca (Maranhao, Brazil)	0.076	0.063		0.005	0.022	0.032	0.034	0.033	0.06	0.06	0.062	0.032	0.033	0.03	0.037	0.055	0.006	0.027	0.029	0.027	0.061	0.061	0.052	0.003	0.058	0.058	0.025	0.024	0.058	0.058	0.055	0.058	0.058	0.057	0.056	0.058
**4**	BC-DZ_Willmott-056 Amigaarnacaarnaca (Para, Brazil)	0.073	0.065	0.005		0.024	0.032	0.034	0.031	0.06	0.06	0.06	0.03	0.032	0.03	0.035	0.053	0.006	0.027	0.024	0.024	0.061	0.061	0.049	0	0.053	0.053	0.02	0.019	0.059	0.056	0.057	0.059	0.059	0.058	0.057	0.059
**5**	BC-DZ_Willmott-057 Amigaarnacaarnaca (Espirito Santo, Brazil)	0.067	0.058	0.022	0.024		0.029	0.022	0.028	0.063	0.062	0.062	0.026	0.021	0.026	0.028	0.05	0.022	0.017	0.019	0.017	0.061	0.061	0.059	0.022	0.058	0.058	0.006	0.005	0.056	0.056	0.055	0.056	0.056	0.059	0.054	0.056
**6**	CP06-76 Amigaarnacaarnaca (Amazonas, Peru)	0.061	0.058	0.032	0.032	0.029		0.027	0.006	0.059	0.059	0.059	0.002	0.005	0.002	0.006	0.046	0.031	0.022	0.024	0.023	0.06	0.06	0.042	0.031	0.055	0.055	0.027	0.029	0.056	0.056	0.051	0.056	0.056	0.055	0.054	0.056
**7**	DNA99-015 Amigaarnacaarnaca (Napo, Ecuador)	0.064	0.074	0.034	0.034	0.022	0.027		0.023	0.073	0.073	0.073	0.026	0.023	0.025	0.031	0.055	0.034	0.011	0.011	0.011	0.075	0.075	0.077	0.034	0.072	0.072	0.022	0.022	0.075	0.075	0.072	0.075	0.075	0.075	0.072	0.075
**8**	LEP-04408 Amigaarnacaarnaca (Morona-Santiago, Ecuador)	0.058	0.058	0.033	0.031	0.028	0.006	0.023		0.06	0.06	0.056	0.002	0.005	0.002	0.01	0.043	0.031	0.023	0.023	0.021	0.06	0.06	0.039	0.031	0.054	0.054	0.026	0.028	0.056	0.056	0.053	0.056	0.056	0.057	0.055	0.056
**9**	LEP-09788 Amigaarnacaadela (Guayas, Ecuador)	0.024	0.026	0.06	0.06	0.063	0.059	0.073	0.06		0	0	0.059	0.061	0.057	0.064	0.041	0.06	0.061	0.061	0.061	0	0	0.036	0.06	0.014	0.014	0.061	0.063	0.015	0.015	0.012	0.015	0.015	0.014	0.013	0.015
**10**	LEP-09930 Amigaarnacaadela (Pichincha, Ecuador)	0.024	0.026	0.06	0.06	0.062	0.059	0.073	0.06	0		0	0.059	0.061	0.057	0.064	0.041	0.06	0.061	0.061	0.061	0	0	0.036	0.06	0.014	0.014	0.061	0.062	0.015	0.015	0.012	0.015	0.015	0.014	0.013	0.015
**11**	LEP-09931 Amigaarnacaadela (Esmeraldas, Ecuador)	0.024	0.027	0.062	0.06	0.062	0.059	0.073	0.056	0	0		0.058	0.061	0.057	0.064	0.041	0.06	0.059	0.059	0.059	0	0	0.036	0.06	0.013	0.013	0.06	0.062	0.015	0.015	0.012	0.015	0.015	0.014	0.013	0.015
**12**	LEP-10696 Amigaarnacaarnaca (Zamora-Chinchipe, Ecuador)	0.058	0.057	0.032	0.03	0.026	0.002	0.026	0.002	0.059	0.059	0.058		0.003	0	0.005	0.045	0.03	0.021	0.021	0.021	0.059	0.059	0.039	0.03	0.053	0.053	0.025	0.026	0.055	0.055	0.05	0.055	0.055	0.054	0.054	0.055
**13**	LEP-10697 Amigaarnacaarnaca (Pastaza, Ecuador)	0.061	0.06	0.033	0.032	0.021	0.005	0.023	0.005	0.061	0.061	0.061	0.003		0.003	0.005	0.046	0.032	0.018	0.018	0.018	0.062	0.062	0.046	0.032	0.055	0.055	0.02	0.021	0.058	0.058	0.053	0.058	0.058	0.057	0.056	0.058
**14**	LEP-10703 Amigaarnacaarnaca (Zamora-Chinchipe, Ecuador)	0.058	0.056	0.03	0.03	0.026	0.002	0.025	0.002	0.057	0.057	0.057	0	0.003		0.005	0.044	0.03	0.023	0.023	0.023	0.058	0.058	0.039	0.03	0.053	0.053	0.025	0.026	0.054	0.054	0.049	0.054	0.054	0.052	0.052	0.054
**15**	LEP-15082 Amigaarnacaarnaca (Morona-Santiago, Ecuador)	0.058	0.062	0.037	0.035	0.028	0.006	0.031	0.01	0.064	0.064	0.064	0.005	0.005	0.005		0.051	0.035	0.023	0.023	0.023	0.064	0.064	0.042	0.035	0.058	0.058	0.026	0.028	0.06	0.06	0.052	0.06	0.06	0.055	0.058	0.06
**16**	LEP-16997 Amigaarnacasericeella (Copan, Honduras)	0.044	0.043	0.055	0.053	0.05	0.046	0.055	0.043	0.041	0.041	0.041	0.045	0.046	0.044	0.051		0.053	0.044	0.044	0.044	0.041	0.041	0.028	0.053	0.041	0.041	0.046	0.048	0.044	0.044	0.044	0.044	0.044	0.047	0.042	0.044
**17**	LEP-34357 Amigaarnacaarnaca (St-Laurent du Maroni, French Guiana)	0.073	0.061	0.006	0.006	0.022	0.031	0.034	0.031	0.06	0.06	0.06	0.03	0.032	0.03	0.035	0.053		0.024	0.027	0.024	0.059	0.059	0.052	0.003	0.056	0.056	0.024	0.022	0.056	0.056	0.053	0.056	0.056	0.055	0.054	0.056
**18**	LEP-37404 Amigaarnacaarnaca (San Martin, Peru)	0.064	0.056	0.027	0.027	0.017	0.022	0.011	0.023	0.061	0.061	0.059	0.021	0.018	0.023	0.023	0.044	0.024		0.003	0	0.059	0.059	0.052	0.024	0.054	0.054	0.016	0.017	0.054	0.055	0.053	0.054	0.054	0.055	0.052	0.054
**19**	LEP-37411 Amigaarnacaarnaca (San Martin, Peru)	0.064	0.059	0.029	0.024	0.019	0.024	0.011	0.023	0.061	0.061	0.059	0.021	0.018	0.023	0.023	0.044	0.027	0.003		0	0.061	0.061	0.052	0.024	0.051	0.051	0.012	0.014	0.058	0.055	0.057	0.058	0.058	0.058	0.056	0.058
**20**	LEP-37416 Amigaarnacaarnaca (San Martin, Peru)	0.064	0.056	0.027	0.024	0.017	0.023	0.011	0.021	0.061	0.061	0.059	0.021	0.018	0.023	0.023	0.044	0.024	0	0		0.06	0.06	0.052	0.024	0.052	0.052	0.013	0.014	0.055	0.055	0.054	0.055	0.055	0.055	0.053	0.055
**21**	LEP-37525 Amigaarnacaadela (Esmeraldas, Ecuador)	0.024	0.026	0.061	0.061	0.061	0.06	0.075	0.06	0	0	0	0.059	0.062	0.058	0.064	0.041	0.059	0.059	0.061	0.06		0	0.036	0.06	0.014	0.014	0.061	0.063	0.014	0.014	0.012	0.014	0.014	0.014	0.013	0.014
**22**	LEP-55465 Amigaarnacaadela (Carchi, Ecuador)	0.024	0.026	0.061	0.061	0.061	0.06	0.075	0.06	0	0	0	0.059	0.062	0.058	0.064	0.041	0.059	0.059	0.061	0.06	0		0.036	0.06	0.014	0.014	0.061	0.063	0.014	0.014	0.012	0.014	0.014	0.014	0.013	0.014
**23**	MGCL-LOAN-028 Amigaarnacaadela (Antioquia, Colombia)	0.049	0.049	0.052	0.049	0.059	0.042	0.077	0.039	0.036	0.036	0.036	0.039	0.046	0.039	0.042	0.028	0.052	0.052	0.052	0.052	0.036	0.036		0.049	0.03	0.03	0.056	0.059	0.03	0.03	0.03	0.03	0.03	0.03	0.03	0.03
**24**	MGCL-LOAN-090 Amigaarnacaarnaca (Para, Brazil)	0.073	0.062	0.003	0	0.022	0.031	0.034	0.031	0.06	0.06	0.06	0.03	0.032	0.03	0.035	0.053	0.003	0.024	0.024	0.024	0.06	0.06	0.049		0.053	0.053	0.021	0.019	0.056	0.056	0.054	0.056	0.056	0.055	0.055	0.056
**25**	MGCL-LOAN-139 Amigaarnacaadela (Choco, Colombia)	0.03	0.029	0.058	0.053	0.058	0.055	0.072	0.054	0.014	0.014	0.013	0.053	0.055	0.053	0.058	0.041	0.056	0.054	0.051	0.052	0.014	0.014	0.03	0.053		0	0.051	0.053	0.005	0.002	0.003	0.005	0.005	0.005	0.003	0.005
**26**	MGCL-LOAN-144 Amigaarnacaadela (Choco, Colombia)	0.03	0.029	0.058	0.053	0.058	0.055	0.072	0.054	0.014	0.014	0.013	0.053	0.055	0.053	0.058	0.041	0.056	0.054	0.051	0.052	0.014	0.014	0.03	0.053	0		0.051	0.053	0.005	0.002	0.003	0.005	0.005	0.005	0.003	0.005
**27**	MGCL-LOAN-162 Amigaarnacaarnaca (Minas Gerais, Brazil)	0.067	0.059	0.025	0.02	0.006	0.027	0.022	0.026	0.061	0.061	0.06	0.025	0.02	0.025	0.026	0.046	0.024	0.016	0.012	0.013	0.061	0.061	0.056	0.021	0.051	0.051		0.002	0.058	0.055	0.057	0.058	0.058	0.06	0.056	0.058
**28**	MGCL-LOAN-217 Amigaarnacaarnaca (Bahia, Brazil)	0.067	0.061	0.024	0.019	0.005	0.029	0.022	0.028	0.063	0.062	0.062	0.026	0.021	0.026	0.028	0.048	0.022	0.017	0.014	0.014	0.063	0.063	0.059	0.019	0.053	0.053	0.002		0.059	0.056	0.058	0.059	0.059	0.062	0.058	0.059
**29**	YB-BCI23691 Amigaarnacaadela (Panama)	0.033	0.028	0.058	0.059	0.056	0.056	0.075	0.056	0.015	0.015	0.015	0.055	0.058	0.054	0.06	0.044	0.056	0.054	0.058	0.055	0.014	0.014	0.03	0.056	0.005	0.005	0.058	0.059		0	0.002	0	0	0	0.002	0
**30**	YB-BCI35406 Amigaarnacaadela (Panama)	0.033	0.028	0.058	0.056	0.056	0.056	0.075	0.056	0.015	0.015	0.015	0.055	0.058	0.054	0.06	0.044	0.056	0.055	0.055	0.055	0.014	0.014	0.03	0.056	0.002	0.002	0.055	0.056	0		0.002	0	0	0	0.002	0
**31**	YB-BCI35436 Amigaarnacaadela (Panama)	0.03	0.027	0.055	0.057	0.055	0.051	0.072	0.053	0.012	0.012	0.012	0.05	0.053	0.049	0.052	0.044	0.053	0.053	0.057	0.054	0.012	0.012	0.03	0.054	0.003	0.003	0.057	0.058	0.002	0.002		0.002	0.002	0.002	0	0.002
**32**	YB-BCI46591 Amigaarnacaadela (Panama)	0.033	0.028	0.058	0.059	0.056	0.056	0.075	0.056	0.015	0.015	0.015	0.055	0.058	0.054	0.06	0.044	0.056	0.054	0.058	0.055	0.014	0.014	0.03	0.056	0.005	0.005	0.058	0.059	0	0	0.002		0	0	0.002	0
**33**	YB-BCI46628 Amigaarnacaadela (Panama)	0.033	0.028	0.058	0.059	0.056	0.056	0.075	0.056	0.015	0.015	0.015	0.055	0.058	0.054	0.06	0.044	0.056	0.054	0.058	0.055	0.014	0.014	0.03	0.056	0.005	0.005	0.058	0.059	0	0	0.002	0		0	0.002	0
**34**	YB-BCI49395 Amigaarnacaadela (Panama)	0.034	0.03	0.057	0.058	0.059	0.055	0.075	0.057	0.014	0.014	0.014	0.054	0.057	0.052	0.055	0.047	0.055	0.055	0.058	0.055	0.014	0.014	0.03	0.055	0.005	0.005	0.06	0.062	0	0	0.002	0	0		0.002	0
**35**	YB-BCI6807 Amigaarnacaadela (Panama)	0.03	0.026	0.056	0.057	0.054	0.054	0.072	0.055	0.013	0.013	0.013	0.054	0.056	0.052	0.058	0.042	0.054	0.052	0.056	0.053	0.013	0.013	0.03	0.055	0.003	0.003	0.056	0.058	0.002	0.002	0	0.002	0.002	0.002		0.002
**36**	YB-BCI766 Amigaarnacaadela (Panama)	0.033	0.028	0.058	0.059	0.056	0.056	0.075	0.056	0.015	0.015	0.015	0.055	0.058	0.054	0.06	0.044	0.056	0.054	0.058	0.055	0.014	0.014	0.03	0.056	0.005	0.005	0.058	0.059	0	0	0.002	0	0	0	0.002	

## Results and discussion

### Molecular phylogeny and species delimitation

**Figure 1.**
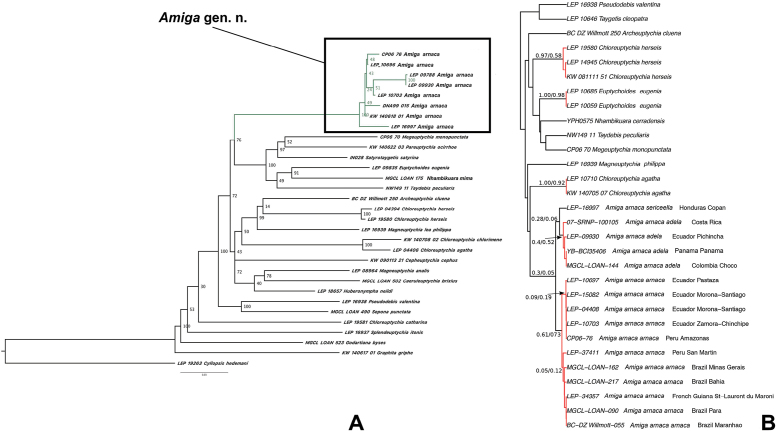
**A** Results of maximum likelihood analysis based on concatenated dataset of 4 genes (COI, EF1a, RPS5, GAPDH) with hybrid enrichment data used as a constraint tree. Support values indicate ultrafast bootstrap. **B** Results from species delimitation analyses based on unique COI sequences only. Clades in red were delimited as separate units in all analyses (GMYC, bPTP and ABGD). Delimitated clades within *A.arnaca* comb. n. are here interpreted as subspecies. Support values indicate the probabilities that the specimens connected at that node constitute a separate unit (GMYC/bPTP).

The ML tree based on four genes and with the 368 gene hybrid enrichment tree used as a constraint tree is shown in Figure 1A. Although deeper relationships are mostly not well supported it is clear that *C.arnaca* is not closely related to the remaining *Chloreuptychia* species, as also found by Espeland et al. (2019) based on 368 loci, as well as phylogeny inferred based on hybrid enrichment data generated for this study (see Suppl. material 1).

All three species delimitation methods delimited the same three “species” within *Amiga* gen. n., with allopatric distributions: eastern Central America to western Ecuador (*A.arnacaadela* ssp. n.), east of the Andes (*A.arnacaarnaca* comb. n.), and Mexico to Honduras (*A.arnacasericeella* comb. n. et stat. n.), although support values are not very high (Fig. 1B). We here decide to consider these taxa as subspecies since *A.arnaca* comb. n. is a morphologically very distinct species itself, and although morphological differences exist between the subspecies they are not very prominent in comparison with other groups of sympatric, closely related euptychiine species. This taxonomic arrangement is reinforced by three subspecies being recovered only in the gene tree inferred using COI and one nuclear gene (GAPDH), but not in any of the trees inferred using EF1a, RPS5 or in the combined tree (Fig. 1A, B; Suppl. material 1). Because nuclear genes evolve slowly and lineage sorting is slower compared to mitochondrial genes, we consider these data to provide evidence for the subspecific status of the taxa discussed below. Braby et al. (2012) defined subspecies in butterflies as partially isolated, allopatric, lineages within a species that are phenotypically distinguishable with at least one fixed diagnosable character state correlated with genetic structure, which matches exactly what we find here. In addition to the three subspecies delimited above we consider another allopatric lineage (*A.arnacaindianacristoi* ssp. n.) as a subspecies based on morphological differences only, as we could not obtain sequence data from this particular taxon.

#### Amiga

Taxon classificationAnimaliaNymphalidaeNymphalidae

Nakahara, Willmott & Espeland gen. n.

http://zoobank.org/ADBD4E75-4ACB-4C53-93D6-D557DA94CB4B

##### Type species.

*Papilioarnaca* Fabricius, 1776, by present designation

##### Systematic placement and diagnosis.

Espeland et al. (2019) recovered *Amigaarnaca* comb. n. as sister to the “*Pareuptychia* clade”, whose composition partially corresponded to that found in Peña et al. (2010), with a high support (BS and PP > 0.95). The “*Pareuptychia* clade” itself was also well supported (BS and PP > 0.95), including *Satyrotaygetissatyrina* (Bates, 1865), *Magneuptychiainani* (Staudinger, [1886]), *Euptychoidesalbofasciata* (Hewitson, 1869), *Neonymphaareolatus* (Smith, 1797), *Erichthodesantonina* (C. Felder & R. Felder, 1867), *Pareuptychiaocirrhoe* (Fabricius, 1776), *Megeuptychiaantonoe* (Cramer, 1775), *Splendeuptychiadoxes* (Godart, [1824]), *Nhambikuaramima* (Butler, 1867), and *Euptychoideseugenia* (C. Felder & R. Felder, 1867). *Amiga* gen. n. is distinguished from all members of the “*Pareuptychia* clade” by the presence of bluish-lilac coloration on the dorsal hindwing and by the purplish sheen in the tornal half of the VHW. Furthermore, the absence of cornuti and membranous lamella antevaginalis of *Amiga* gen. n. appear to be unusual character states among the clade. The type species of *Chloreuptychia*, *Papiliochloris* Cramer, 1780 (= *Chloreuptychiachlorimene*) was recovered as sister to a moderately supported (BS and PP > 0. 75 < 0. 95), clade including the “*Pareuptychia* clade”, “*Taygetis* clade”, “*Splendeuptychia* clade” and “*Archeuptychia* clade”.

##### Description.

**MALE**: Forewing length: 18.5–22 mm (*n* = 6)

**Head**: Eyes with sparse golden hair-like setae, with white scales along margin; frons dark brownish, covered with creamy brownish scales and hair-like scales towards antennae; first segment of labial palpi similar in width to second segment, similar in length to third segment, adorned with white long hair-like scales and brownish long hair-like scales ventrally, second segment longer than eye depth and covered with white hair-like scales, brownish hair-like scales, and white scales laterally and dorsally, with brownish hair-like scales and brownish scales along edge of distal two-thirds of dorsal surface, ventrally adorned with brownish hair-like scales and whitish hair-like scales longer than segment width, third segment roughly one-third of second segment in length and covered with brownish scales dorsally and ventrally, with white scales laterally; antennae approximately half of forewing length, with ca 34 segments (*n* = 1), scape about as twice as long as pedicel, white scales on each side of base of flagellomeres, distal 10–12 segments composing club.

**Thorax**: Brownish, dorsally scattered with grayish scales and lightly colored long hair-like scales; ventrally scattered with white scales and white long hair-like scales; foreleg (Fig. 5c) whitish, femur, tibia and tarsus similar in length; midleg and hindleg with femur white ventrally, tibia and tarsus grayish dorsally, ocher ventrally tibia and tarsus adorned with spines ventrally, pair of tibial spurs present at distal end of tibia.

**Abdomen** (Fig. 3a, b): Eighth tergite sclerotized in a narrow anterior band and slightly broader posterior patch, which appear as two separate plates.

**Wing venation** (Fig. 3): Basal half of forewing subcostal vein swollen; base of cubitus swollen; forewing recurrent vein present as small projection slightly above origin of M_2_; hindwing humeral vein developed; origin of M_2_ slightly towards M_1_ than M_3_.

**Wing shape**: Forewing triangular, apex rounded, costal margin slightly convex, outer margin almost straight below M_2_, inner margin almost straight, but curving inwards towards thorax near base; hindwing somewhat rectangular, slightly elongate, costal margin almost straight, angled inwards at base, outer margin slightly undulating, inner margin slightly curved near tornus, anal lobe convex, slightly rounded.

**Dorsal forewing**: Ground color light brownish, slightly translucent, thus subtly revealing ventral bands and ocelli in cell M_1_.

**Dorsal hindwing**: Ground color similar to forewing, iridescent bluish lilac reflection covering most of dorsal hindwing, area near costa and area distal to marginal band revealing ground color; slightly translucent, thus subtly revealing ventral bands and ocelli.

**Ventral forewing**: Ground color pale grayish brown; pale reddish-brown discal band extending from radial vein, crossing discal cell, passing origin of Cu_2_, terminating at 2A; concolorous scales present along discocellular vein; pale reddish-brown postdiscal band extending from radial vein towards inner margin until reaching vein 2A, slightly wider than discal band; broad, faint, iridescent bluish-lilac reflection extending just distal of postdiscal band towards outer margin, prominent between postdiscal band and umbra; sinuate, narrow submarginal band, almost concolorous to basal two bands, extending from apex towards tornus, jagged above Cu_1_, almost straight below this vein; concolorous marginal band, not jagged, appearing narrower than submarginal band, traversing along marginal area from apex to tornus; fringe brownish; ocellus in cell M_1_, spilling out from veins M_1_ and M_2_, black with two whitish pupils in center, ringed in yellowish orange, ocelli present in cell M_2_ and M_3_, appearing as two slate-gray patches surrounded by yellowish-orange ring; umbra appearing as broad rather faint band concolorous to four ventral bands, visible around ocelli, extending to cell Cu_2_.

**Ventral hindwing**: Ground color similar to forewing; general wing pattern similar to forewing except as follows: iridescent bluish-lilac reflection extending from base of wing, towards outer and inner margin, especially area where iridescent bluish scales are present on dorsal surface; discal band passing cubital vein area basal to origin of Cu_2_; postdiscal band passing origin of Cu_1_, bent inwards in cell Cu_2_; submarginal band broadening towards tornus after passing Cu_1_; five submarginal ocelli, those in cells M_1_ and Cu_1_ similar to that in VFW cell M_1_ but with single pupil, those in cells M_2_ and M_3_ similar to those in VFW cells Rs, M_2_ and M_3_ but slate grayish patch appearing as single patch in middle.

**Genitalia** (Fig. 4c–g): Tegumen appears semi-circular in lateral view, anteriorly and dorsally convex, ventral margin rather straight; uncus longer than tegumen in lateral view, apparently without setae, middle section somewhat broadening in dorsal view, tapering posteriorly and terminating in single point; brachium tapering towards apex, apical point positioned above uncus in lateral view, parallel to uncus with apical edge curving inwards in dorsal view; combination of ventral arms from tegumen and dorsal arms from saccus slightly curved distally; appendices angulares present; saccus narrow, concavity at base of ventral margin, anteriorly rounded, similar or shorter than uncus in length; juxta present as plate with deep concavity at dorsal margin in posterior view; valva distally setose, valva appearing roughly parallelogram in lateral view, ventral margin convex, dorsal margin distal of costa curved, costa curved inwards, apical process somewhat curving upwards; phallus roughly straight, similar in length to valva plus saccus, phallobase about one-third of phallus, ductus ejaculatorius visible as illustrated, posterior portion of aedeagus somewhat curved upwards, manica covering more than half of aedeagus, cornuti absent.

**FEMALE**: forewing length: 19–21 mm (*n* = 6)

**Similar to male except as follows**: Five tarsomeres present in foretarsus, with spines along some tarsomeres; forewing appearing somewhat rounded and broad; dorsal hindwing submarginal band somewhat more prominent; bluish lilac reflection appearing more purplish, extending to origin of M_1_ or further anteriorly; feeble pearly reflection present on dorsal forewing (but see below for further information). **Female genitalia and abdomen** (Figs 4h–k): Inter-segmental membrane between 7^th^ and 8^th^ abdominal segments pleated and expandable, with weakly sclerotized region present; lamella antevaginalis membranous; lateral side of 8^th^ abdominal segment sclerotized (referred to as “lamella postvaginalis” by Willmott et al. 2018), this sclerotized plate fused to lamella antevaginalis at anterior margin; ductus bursae membranous, somewhat inflated around origin of ductus seminalis, located at approximately one-fifth distance from ostium bursae to corpus bursae, ductus bursae apparently weakly sclerotized at region posterior to origin of ductus seminalis; corpus bursae roughly oval in dorsal view, with two relatively narrow signa, together with ductus bursae extending to juncture of 4^th^ and 5^th^ sternite.

##### Variation.

This species exhibits geographic variation in wing pattern, some of which is recognized here with subspecific names. A broad, faint, iridescent bluish-lilac reflection between the ventral forewing postdiscal band and the umbra is present in specimens from the eastern Andes, whereas it is absent in many specimens from west of the Andes. The absence of this bluish-lilac reflection on the ventral forewing seems rather stable in specimens from western Colombia and western Ecuador. However, this character appears in a few specimens from Panama, and is present in some specimens from Costa Rica and Nicaragua, although the degree of reflection is variable. The bluish-lilac reflection seen mainly on both the dorsal forewing and hindwing is variable in color, varying from light blue to purple. Especially in female specimens, the extent of bluish-lilac reflection on the dorsal forewing is variable, being absent in some specimens, whereas covering most of the discal cell and cells Cu_1_ and Cu_2_ in others (see below for further information). There exists a feeble pearly reflection on the ventral forewing from the base to the postdiscal band in specimens from southeastern Brazil (see below for further information), although this is absent in a few specimens. The presence or absence of a ventral forewing ocellus in cell M_3_ is variable, appearing as a trace in some specimens. The size of the ventral hindwing ocellus in cell M_1_ is variable in comparison with the ventral hindwing ocellus in cell Cu_1_, ranging from similar in size to almost twice as large. The genitalia appear not to be informative in separating specimens from east and west of the Andes, although specimens from Central America, including *A.arnacasericeella* comb. n. et stat. n., seem to have a rather curved dorsal margin of the uncus in lateral view (Fig. 4e).

##### Etymology.

The new generic name is derived from the feminine Spanish noun “amiga”, meaning “a (female) friend”, alluding to the fact that this is a common, familiar butterfly. The generic name is regarded as feminine.

##### Biology.

Janzen and Hallwachs (2018) report four grass species (Poaceae), *Ichnanthusnemorosus*, *Ichnanthuspallens*, *Lasiacisruscifolia*, and *Paspalumdecumbens*, as hostplants for *Amiga* gen. n. in Costa Rica. In addition, DeVries (1987) reported three grass genera, namely *Eleusine*, *Ichnanthus*, and *Oplismenus*, as hostplants in Costa Rica. Singer and Ehrlich (1993) reported *Ichnanthuspallens* as a hostplant in Trinidad and Tobago. The egg, mature larva, and pupa of *Amiga* gen. n. were described and the latter two stages illustrated in DeVries (1987). Various images of the penultimate instar, ultimate instar, prepupa and pupa are figured by Janzen and Hallwachs (2018), based on material reared in Costa Rica. In Costa Rica, adult females of *Amiga* gen. n. were seen ovipositing late in the afternoon, and some eggs were observed to be parasitized by trichogrammatid wasps (DeVries 1987). The species occurs from sea level to at least 1850 m, and it is common, indeed ubiquitous, in undisturbed to heavily disturbed rain and cloud forest. Both sexes fly low (0.5 m) along shady trails throughout the middle of the day (09:00–15:00), and males are often observed perching singly on tops of leaves, maintaining apparent territories, and patrolling for ca 10 m along a trail.

##### Distribution

(Fig. 6). This genus ranges from southern Mexico throughout virtually all of tropical Central and South America, where its southernmost distribution appears to be southern Brazil.

## Taxonomy

*Amiga* gen. n. is regarded as monotypic, with total of four subspecies recognized, of which two are named and described herein.

*Amiga* Nakahara, Willmott & Espeland, gen. n.

(– denotes a subspecies, – – denotes a synonym)

*arnaca* (Fabricius, 1776) comb. n.

– –*ebusa* (Cramer, 1780)

– –*priamis* (D’Almeida, 1922)

–*adela* Nakahara & Espeland, ssp. n.

–*sericeella* (Bates, 1865) comb. n. et stat. n.

–*indianacristoi* Nakahara & Marín, ssp. n.

### Amiga arnaca arnaca

Taxon classificationAnimaliaNymphalidaeNymphalidae

(Fabricius, 1776) comb. n.

**Figure 2.**
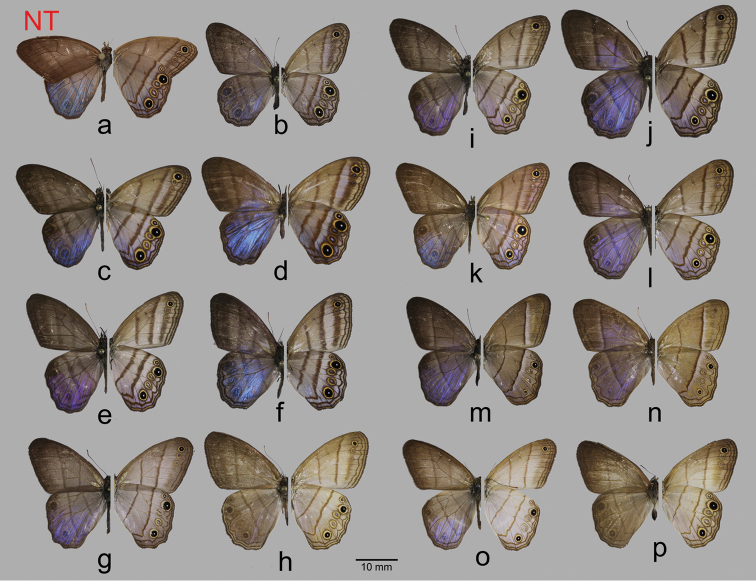
*Amigaarnaca* spp. specimens spanning its range (dorsal on left, ventral on right): **a** nominotypical subspecies from Suriname, neotype male (USNM) **b** nominotypical subspecies from Guyana, female (FLMNH-MGCL 263373) **c** nominotypical subspecies from E Ecuador, male (FLMNH-MGCL 257121) **d** nominotypical subspecies from Peru, female (FLMNH-MGCL 262953); **e** nominotypical subspecies from N Brazil, male (FLMNH-MGCL1036223) **f** Nominotypical subspecies from N Brazil, female (FLMNH-MGCL 207984) **g** Nominotypical subspecies from SE Brazil, male (FLMNH-MGCL 1036213); **h** nominotypical subspecies from SE Brazil, female (FLMNH-MGCL 1036218) **i***A.arnacaadela* from Costa Rica, male (FLMNH-MGCL 207991) **j***A.arnacaadela* from Costa Rica, female (FLMNH-MGCL 207992) **k***A.arnacaadela* from W Ecuador, holotype male (FLMNH-MGCL 151127) **l***A.arnacaadela* from W Ecuador, female (FLMNH-MGCL 257087) **m***A.arnacasericeella*, male from Mexico (FLMNH-MGCL 207900) **n***A.arnacasericeella* from Mexico, female (FLMNH-MGCL 207896) **o***A.arnacaindianacristoi* from NW Venezuela, paratype male (FLMNH-MGCL 263107) **p***A.arnacaindianacristoi* from N Venezuela, paratype female (FLMNH-MGCL 1036235).

**Figure 3.**
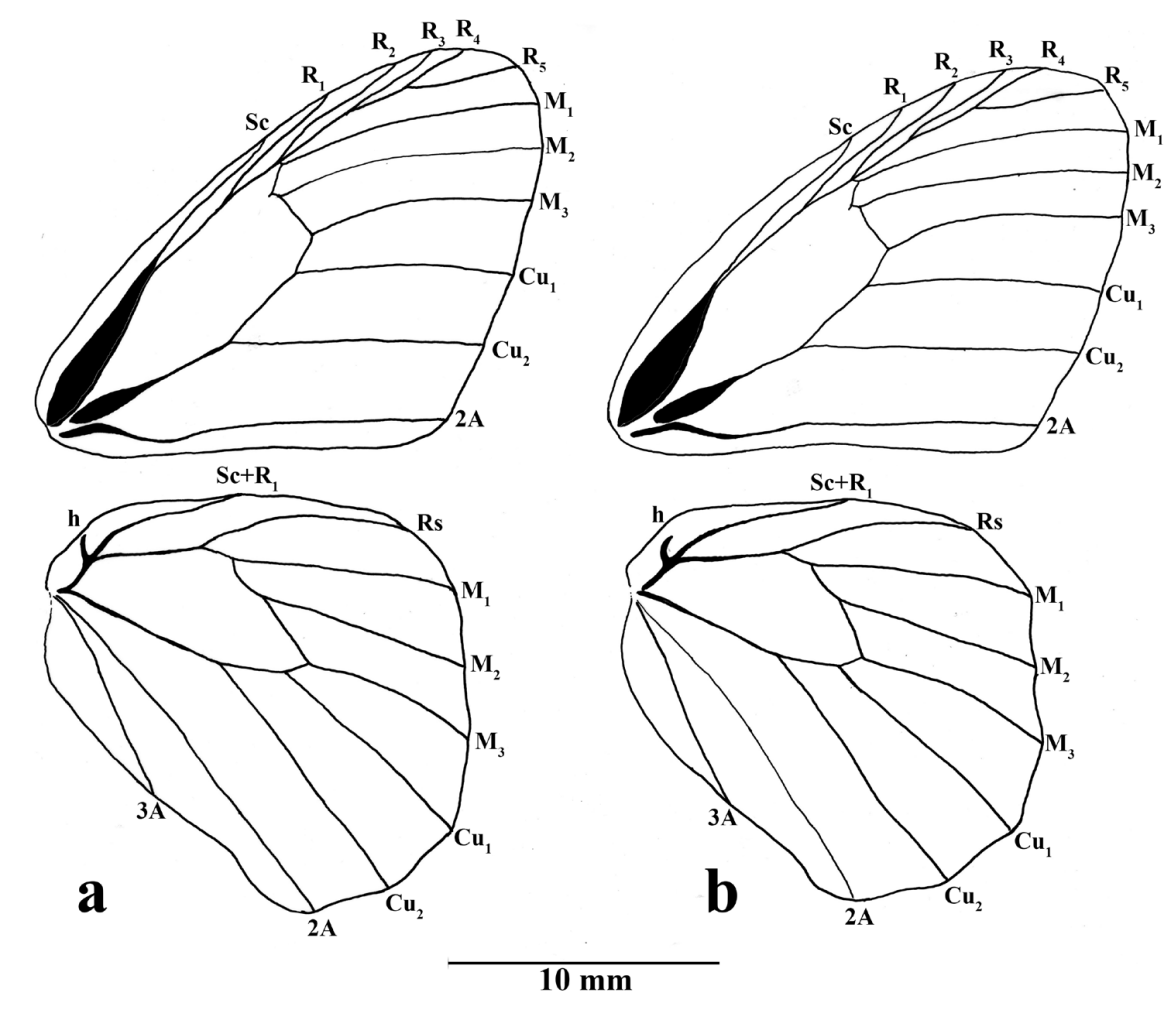
*Amigaarnacaarnaca* comb. n. wing venation: **a** male (FLMNH-MGCL specimen 257164) **b** female (FLMNH-MGCL specimen 257166).

**Figure 4.**
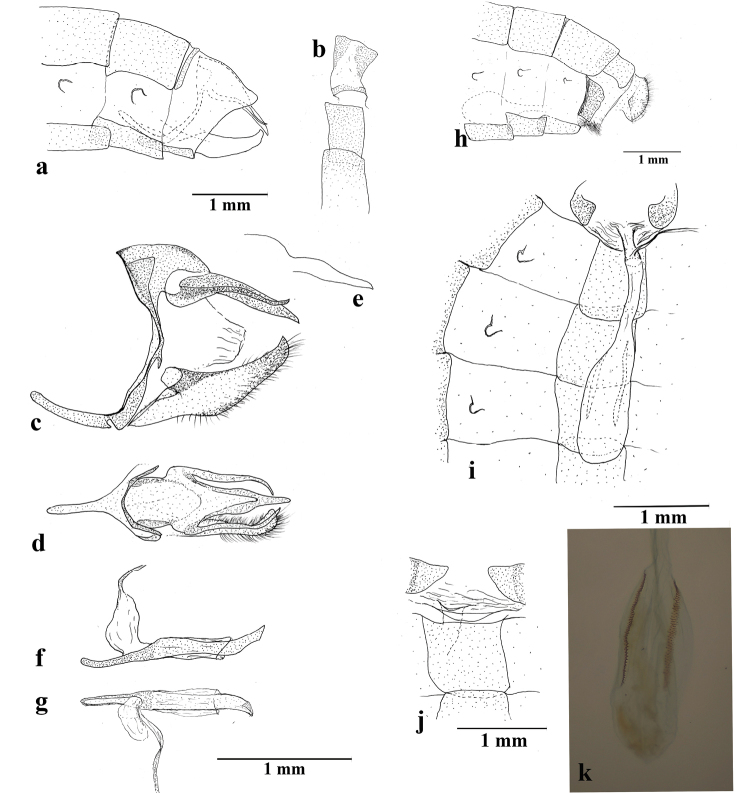
*Amigaarnacaarnaca* comb. n. abdomen and genitalia (*A.arnacaadela* ssp. n. for Fig. 4e): **a** male abdomen terminal sclerites in lateral view **b** male terminal tergites in dorsal view **c** male genitalia in lateral view **d** male genitalia in dorsal view **e** uncus in lateral view, based on KW-15-73 (FLMNH-MGCL Specimen 207904) **f** phallus in lateral view **g** phallus in dorsal view **h** female abdomen terminal sclerites in lateral view, based on SN-17-235 (FLMNH-MGCL Specimen 263371) **i** female genitalia in dorsal view **j** lamella antevaginalis in ventral view **k** signa. Illustrated genitalia: SN-17-148 for male (FLMNH-MGCL specimen 257164); SN-17-150 for female (FLMNH-MGCL specimen 257166), unless indicated otherwise.

**Figure 5.**
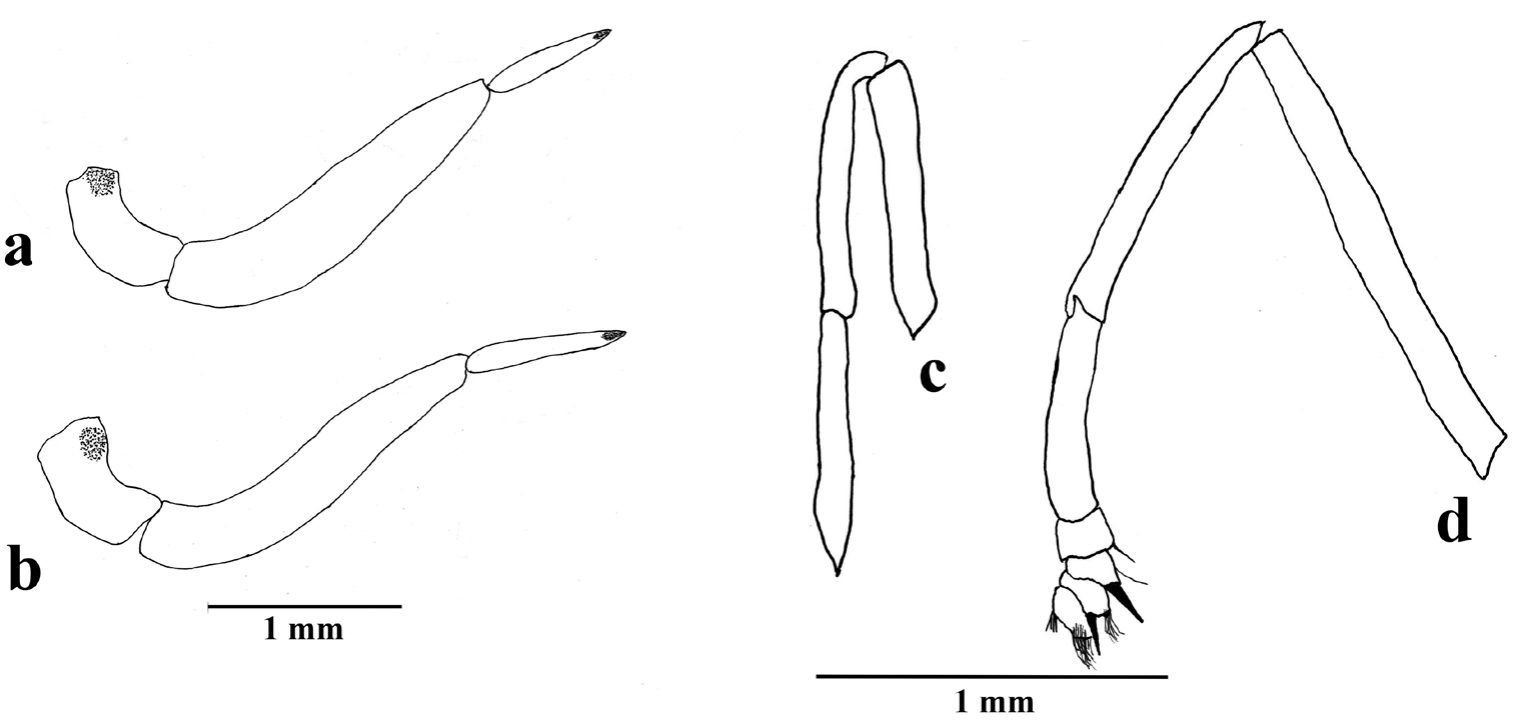
*Amigaarnacaarnaca* comb. n. appendages: **a** male palpus with Reuter’s sensitive patch and Vom Raths organ indicated by dots **b** female palpus with Reuter’s sensitive patch and Vom Rath’s organ indicated by dots **c** male foreleg **d** female foreleg (illustration: FLMNH-MGCL specimen 257163 for male; FLMNH-MGCL specimen 257167 for female).

**Figure 6.**
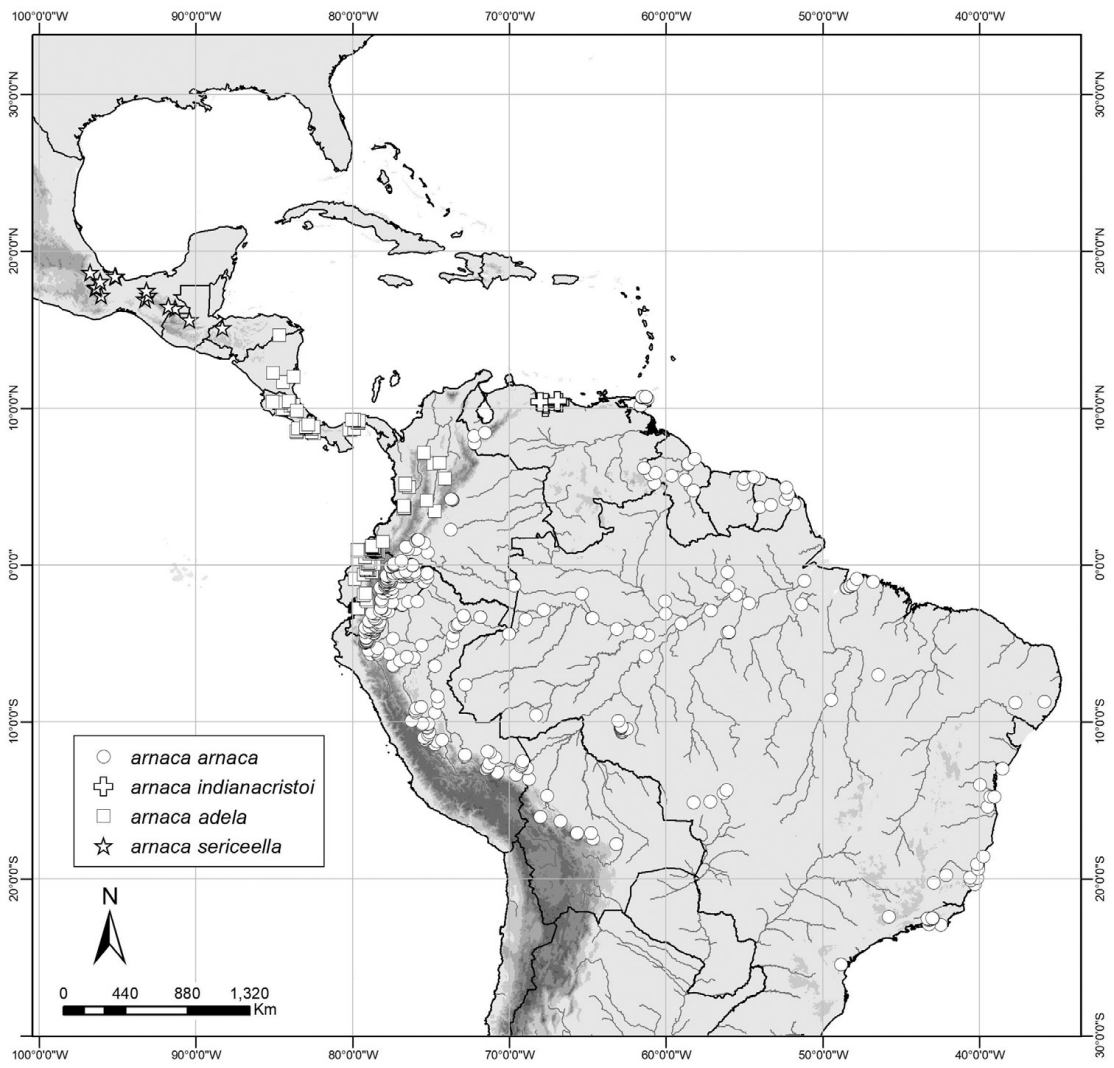
Distribution map of *Amigaarnaca* ssp.

Figs 2a–h , 3 , 4a–d , 4f–k , 5 , 6

Papilio arnaca Fabricius (1776: 260–261). **Type locality**: Suriname. **Neotype** ♂ **(here designated)**: // Suriname Brokopondo Brownsberg, rainforest km 6–12, 30.1.1982, Olle Pellmyr // USNMENT 00913953 // (USNM) [examined]
= Papilioebusa Cramer (1780: 9; pl. CCXCII: figs F, G). **Type locality**: Suriname. **Neotype** ♂ **(here designated)**: Suriname Brokopondo Brownsberg, rainforest km 6–12, 30.1.1982, Olle Pellmyr // USNMENT 00913953 // (USNM) [examined]
= Euptychiaarnaea [sic] formpriamisD’Almeida (1922: 99). **Type locality**: Três Rios, Jacarepaguá [Rio de Janeiro (city), Rio de Janeiro (state), Brazil]. **Holotype** ♂: // Euptychia ar-naea priamis d’Alm. 1922. ♂ // Jacarépaguá, Tres-Rios. Rio, 19.. ♂ // HOLOTIPO// Coll. D’Almeida // No 5630 // DZ 34.684// (DZUP) [examined]
Papilio arnaea [sic]: Fabricius 1781: 85; Fabricius 1787: 37; Kirby 1871: 53; Butler and Druce 1874: 337; Butler 1876: 489; Kaye 1904: 181; Hall 1939: 34.
Papilio aranea [sic]: Fabricius 1793: 97.
Satyrus aranea [sic]: Godart [1824]: 492; Butler 1867: 489.
Euptychia ebusa : Butler 1867: 489; Möschler 1877: 323; Kirby 1879: 135; Godman and Salvin 1880–1881: 88–89; Dyar 1914: 143.
Euptychia arnaea [sic]: Kirby 1871: 53; Butler and Druce 1874: 337; Butler 1877: 122; Möschler 1877: 323; Godman and Salvin 1880–1881: 88–89; Sharpe 1890: 569; Kaye 1904: 181; Weymer 1911: 219, pl. 49d; Hall 1939: 34; Whittaker 1983 (misspelled as “arneae” in figs 1, 2); D’Abrera 1988: 770–771; Emmel and Austin 1990: 10; Mielke and Casagrande 1991: 181; Cock 2014: 11.
Euptychia arnaea [sic] formpriamis: D’Almeida 1937: 254; Brown 1975: 41.
Euptychia arnea [sic]: Gaede 1931: 439; Barcant 1970: 143, pl. 13, fig. 18.
Euptychia arnea [sic] var.priamis: Gaede 1931: 439.
Euptychia arnaca arnaca : Bryk 1953: 63.
Chloreuptychia arnea [sic]: Forster 1964: 120, fig. 131.
Chloreuptychia arnaea [sic]: DeVries 1987:271, pl. 48 figs 18, 19; 261, figs B, C; Ramos 1996: 40; Brown and Freitas 2000: 105.
Cissia arnaea [sic]: Singer and Ehrlich 1993 251, fig. 1.
Chloreuptychia arnaca : Lamas 1994: 165; Lamas and Grados 1996: 58; Lamas et al. 1996: 65; Lamas et al. 1999: 10; Lamas 2004: 218; Beccaloni et al. 2008: 328; Brévignon 2008: 70; Marín and Uribe 2009: 24; Peña et al. 2010: 246; Francini et al. 2011: 65; Paluch et al. 2011: 235; Brévignon and Benmesbah 2012: 52; Cock 2014: 11; Freitas et al. 2016: 320; Paluch et al. 2016: 4.

#### Identification and taxonomy.

*Papilioarnaca* Fabricius, 1776 was described based on an unspecified number of specimens from Suriname, in Johann Dominicus Schulze's collection (Fabricius 1776). Fabricius' description was not accompanied by any illustration of this species, and he did not specify either the sex nor the number of specimens he examined. However, his Latin description is somewhat precise and the identity of the species may be guessed from the description, given the mention of the following wing pattern characters: “forewing, towards the apex there are three ocelli: the distalmost ("exteriori") bi-pupilled”; “hindwings bluish; under surface with five ocelli”; “Hindwings bluish above, iridescent; below bluish with two oblique dark stripes. Submargin with five ocelli, the first and fourth the largest and black, the remainder dark”. The mention of multiple ocelli on the (ventral) forewing excludes the possibility of this specimen being other “*Chloreuptychia*” species, which also possess bluish iridescent coloration, but have only a single ocellus on the ventral forewing. Fabricius (1793) considered *P.lea* Cramer, 1777 and *P.arnaca* as probably being conspecific, and these two names were associated by some subsequent authors (e.g., Godman and Salvin 1880–1881), although the mention of five ventral hindwing ocelli for *P.arnaca* is inconsistent with the six ocelli on the ventral hindwing of *P.lea*. On the other hand, *Neonymphairis* C. Felder & R. Felder, 1867 and *Euptychiatricolor* Hewitson, 1850, two species now placed in *Magneuptychia* Forster, 1964, also match the aforementioned wing pattern characters provided in Fabricius’ original description. These two species do occur in Suriname, and based on the description provided for *P.arnaca*, it is difficult to exclude the possibility of Fabricius having examined one of these two species. Regardless of this fact, the name *arnaca* has been applied in numerous publications and collections to the species as it is identified here (e.g., Whittaker 1983). Considering this situation, stabilizing the nomenclature as currently perceived by many others is crucial regarding the specific epithet *arnaca* and a neotype is therefore designated for this name below.

A worn specimen (whose sex cannot be confidently determined) in William Hunter’s entomological collection is at the UMG, and it was photographed by GL as a potential type specimen of *P.arnaca* (see Warren et al. 2018). GL assumed that William Hunter may have received a "duplicate" from Schulze through Fabricius, who did indeed supply Hunter with duplicates, resulting in many Fabrician type specimens being found in Hunter’s insect collection (Hancock 2015; Tuxen 1967). However, we have found no evidence to support that this specimen was originally in Johann Dominicus Schulze's collection, and the Surinamese provenance of the specimen is questionable given its rather narrow ventral bands, which are typical of *A.arnacaindianacristoi* ssp. n. rather than of specimens from Suriname. The specimen in Glasgow is missing its head and abdomen, in addition to having worn and faded wings, thus somewhat obscuring its true identity. Given this situation, combined with the fact that no authentic Schulze specimens appear to be in existence (e.g., Benmesbah et al. 2018), in addition to the explanation above, we therefore designate a male specimen from Suriname (type locality) as a neotype for *P.arnaca* following Article 75.3 of the ICZN (1999) (**neotype designation**): //Suriname Brokopondo Brownsberg, rainforest km 6-12, 30.1.1982, Olle Pellmyr // USNMENT 00913953// (USNM). (Fig. 2a).

After introducing this species to science, the specific epithet was misspelled as “Arnaea” by Fabricius himself in 1781 and 1787, and as “Aranea” in 1793. Subsequently, the specific epithet *arnaca* has been erroneously spelled in various ways in a disturbingly high number of publications (e.g., Butler and Druce 1874; Butler 1877; Godman and Salvin 1881; Sharpe 1890; Kaye 1904; Weymer 1911; D’Almeida 1922; Gaede 1931; Forster 1964; Brown 1975; Whittaker 1983; DeVries 1987; see also above), including some influential works on the classification of this group. This confusion surrounding its species-group name adds a special urgency for a neotype designation for this common butterfly.

*Papilioebusa* Cramer, 1780 was described in Pieter Cramer’s *De uitlandsche Kapellen voorkomende in de drie Waereld-Deelen Asia, Africa en America*. The original description describes the bluish-lilac reflection on both wing surfaces, although no further description of any wing element was provided in Cramer’s Dutch and French description. Instead, Cramer compared *P.ebusa* to *P.junia* Cramer, 1780, an immediately preceding species described and named in Cramer (1780), but regarded as a junior subjective synonym of *P.lea* Cramer, 1777 by Lamas (2004). Evidently, *P.ebusa* and *P.junia* are not conspecific judging from the illustrations in Cramer (1780: 9; pl. CCXCII: figs D–G), and the illustrations of *P.ebusa* combined with Cramer’s description enable this taxon to be confidently identified. *Papiliochloris* Cramer, 1780 (now known as *Chloreuptychiachlorimene* (Hübner, [1819])), is perhaps the only taxon known from Suriname which might have resulted in a similar illustration; however, the illustration of *P.chloris* provided by Cramer (1780: CCXCIII: figs A, B) excludes this possibility. Based on the Dutch and French description provided for *P.ebusa*, Cramer based his illustration on what he thought was a female specimen, although the illustration of the dorsal surface (Fig. F) showing the bluish-lilac reflection only on the hindwing indicates that this illustrated specimen is likely to be a male (but see also above for further information). In addition, whether the original description was based on a single specimen or several specimens cannot be unambiguously determined. During our attempt to locate syntype(s) of *P.ebusa*, two specimens with rounded labels indicating “[Johan] Calkoen” with the locality “Brasilia” were found in RMNH. Along with the collection of Joan Raye Heer van Breukelerwaard, Johan Calkoen’s collection includes Cramer types, although given the locality “Brasilia”, these two specimens are most likely not syntypes of *P.ebusa*. Considering that we were unable to find any additional possible syntype(s) of *P.ebusa*, we here designate a neotype for this name. Although treated as a valid species in the past (e.g., Butler 1867; Godman and Salvin 1880–1881), in order to maintain its status as a junior synonym of *P.arnaca*, first recognized by Kirby (1871) and followed by most subsequent authors (e.g., Weymer 1911; Gaede 1931; Lamas 2004), we designate the specimen designated as the neotype of *P.arnaca* as the neotype of *P.ebusa* as well and retain its synonymy as a junior objective synonym (**neotype designation**).

D’Almeida (1922) described *Euptychiaarnaea* [sic] formpriamis based on a single male from Três Rios, Jacarepaguá, Rio de Janeiro, Rio de Janeiro, Brazil, currently housed at the DZUP. Following Article 73.1.2. of the ICZN (1999), we consider this male specimen to be the holotype fixed by monotypy based on the statement of “one male collected at the type locality” provided in the original description (D’Almeida 1922). Lamas (2004) regarded this taxon as a junior subjective synonym of *Papilioarnaca* without providing any justification. D’Almeida’s (1922) original description provides some wing pattern characters which he considered to separate f.priamis, namely “Underside, feeble pearly reflections extending from the base to the line of ocelli”; “Underside, the two rays in the middle are narrow”. These two wing pattern characters are seen in the holotype male, and indeed, the overall phenotype of specimens from the Brazilian states of Minas Gerais, Espírito Santo, and Rio de Janeiro does look somewhat different compared to the neighbouring nominotypical subspecies. Although the feeble pearly reflection extending from the base of the ventral forewing is not seen in the nominotypical subspecies, a few specimens from the aforementioned states in southeastern Brazil appear to lack this reflection (e.g., FLMNH-MGCL-1036218). The narrow ventral bands of many specimens from southeastern Brazil resemble those of *A.arnacaindianacristoi* ssp. n., although the ventral bands are slightly variable in width and a few specimens (e.g., FLMNH-MGCL-262982, 263014) possess bands that are similar in width to the nominotypical subspecies. Thus, the majority of the specimens from Minas Gerais, Espírito Santo, and Rio de Janeiro are distinguishable from the nominotypical subspecies based on the aforementioned characters except for specimens from Bahia consistently possessing wider ventral bands and/or lacking the feeble pearly reflections on the ventral surface. Nevertheless, we decided not to treat *A.arnaca* from Minas Gerais, Espírito Santo, and Rio de Janeiro as a distinct subspecies because, based on molecular data, this taxonomy would result in the nominotypical subspecies being paraphyletic. Whether subspecies should simply represent geographical variation or should also represent an evolutionary unit (i.e. a monophyletic group) is not a focus of this study and this question merits further in-depth discussion and more data. To be consistent in terms of the subspecies concept used in this study, we consider that subspecies should ideally represent clades, unless there is a strong counter-argument, and thus retain the synonymy introduced in Lamas (2004).

#### Distribution

(Fig. 6). The nominotypical subspecies occurs from eastern Colombia south to Bolivia, and in Brazil, southern Venezuela and the Guianas, where it is typically common and widespread in lowland to submontane forest.

#### Examined specimens

(718 ♂, 207 ♀). See Appendix for the data of these specimens.

### Amiga arnaca adela

Taxon classificationAnimaliaNymphalidaeNymphalidae

Nakahara & Espeland subsp. n.

http://zoobank.org/737B03DC-05CC-4D17-B734-13E3E3B38918

Figs 2i–l , 3d , 6

#### Description and diagnosis.

**MALE**: forewing length 19–21 mm (*n* = 5): Differs from the nominotypical subspecies in the following respects: broad, faint, iridescent bluish-lilac reflection between the ventral forewing postdiscal band and the umbra is absent (but see also below).

**FEMALE**: forewing length 21–22 mm (*n* = 4): Differs from the nominotypical subspecies by the same ventral forewing character provided for the male.

#### Variation.

As explained under the variation section under the genus, the absence of the bluish-lilac reflection on the ventral forewing is consistent in specimens from western Colombia and western Ecuador. However, this character appears in a few specimens from Panama, Costa Rica, and Nicaragua (e.g., FLMNH-MGCL 208036, 257145, 263067), although the degree of reflection is variable.

#### Types.

**HOLOTYPE** ♂: // ECUADOR: *Esmeraldas*, W Maldonado–Selva Alegre rd., El Cerro 235 m, 0°58'22"N,78°55'19"W 21, 25.vii.2011, K. Willmott, & J. Hall FLMNH-MGCL-151127 // DNA voucher LEP-09931 // (FLMNH, to be deposited in INABIO). (Fig. 2k).

#### Other examined specimens

(244 ♂, 104 ♀). See Appendix for the data of these specimens. These specimens are not included in the type series as labelling will likely not be completed for over 300 specimens.

#### Etymology.

This species-group name is based on the Greek adjective “adelos”, meaning “indistinct” or “inconspicuous”, in reference to its lack of bluish reflection on the ventral forewing. This species-group name is treated as a Latinized feminine adjective in accordance with the feminine generic name.

#### Distribution

(Fig. 6). This subspecies occurs from Nicaragua to western Ecuador.

### Amiga arnaca sericeella

Taxon classificationAnimaliaNymphalidaeNymphalidae

(Bates, 1865), comb. n. et stat. n.

Figs 2m, n , 6

Euptychia sericeella Bates (1865: 202). **Type locality**: Vera Paz, Guatemala. **Lectotype (here designated)** ♀: Godman-Salvin Coll. 1904.–1. B.C.A. Lep. Rhop. Euptychiasericeella, Bates. // B.M. TYPE No. Rh 3181 Euptychiasericeella, ♀ Bates. // Forests of N. Vera Paz. F.D.G. & O.S. // Type. Sp. Figured. // ♀ // (NHMUK) [examined].
Euptychia sericeella : Butler 1867: 489; Kirby 1871: 53; Butler 1877: 122; Godman and Salvin 1880–1881: 89–90, pl. 8, figs 20, 21; Weymer 1911: 219; Riley and Gabriel 1924: 53; Gaede 1931: 464; D’Abrera 1988: 770–771.
Chloreuptychia sericella [sic]: Forster 1964: 120–121, fig. 132.
Chloreuptychia sericeella : R. de la Maza and J. de la Maza 1993: 182; Salinas-Gutiérrez et al. 2004: 136; Lamas 2004: 218.

#### Identification and taxonomy.

Bates (1865) described *Euptychiasericeella* based on an unspecified number of “male” specimen(s) from Vera Paz, Guatemala. Nevertheless, the only syntype specimen that we have located, in the NHMUK, is a female, a fact also noted by Godman and Salvin (1880: 89–90), who had this specimen in their possession and referred to it as “our specimen, marked as the type”. This specimen (B.M. TYPE No. Rh 3181) was also referred to as a type by Riley and Gabriel (1924: 53) and D’Abrera (1988: 770–771). Because of the complexity of euptychiine taxonomy, to provide an unambiguous reference for this name we here designate this specimen as the lectotype of *Euptychiasericeella* (**lectotype designation**). Bates explicitly stated that the taxon was closely allied to *Eu*[*ptychia*]*. ebusa* (= *Amigaarnacaarnaca* comb. n.), indicating that he clearly regarded *Euptychiasericeella* and *Amigaarnacaarnaca* as two different species. Despite the ambiguous diagnosis provided in the original description (“the fore-wing having a narrow costal border, the apex, and a broader outer border of a brown hue”), the lectotype, figured in Warren et al. (2018), exhibits several rather distinctive phenotypic differences compared to specimens from South America and Nicaragua to western Ecuador. Presumably due to these wing pattern differences, described further below, subsequent authors treated *E.sericeella* as a species-level taxon (e.g., Butler 1867, 1877; Weymer 1911; Gaede 1931; Forster 1964; Lamas 2004). Specimens from Zelaya department, Nicaragua (e.g., FLMNH-MGCL-263066, 263067, 263072) are phenotypically similar to specimens known from Costa Rica to western Ecuador, and *A.a.sericeella* thus seems to replace *A.a.adela* n. ssp. with little or no obvious gap or dispersal barrier separating these taxa, and conversely with no known area of sympatry. Furthermore, the presence of an iridescent bluish-lilac reflection between the postdiscal band and umbra in some specimens of the subspecies from Nicaragua, Costa Rica, and Panama might be a result of introgression with *A.a.sericeella*. Finally, divergence in the DNA barcode between a specimen from Honduras (LEP-16997) phenotypically similar to the lectotype of *sericeella* and other Central American *A.arnaca* from further south is no greater than between east and west Andean *A.arnaca* (Table 2). Based on these considerations, we downgrade *E.sericeella* to subspecific rank, proposing, for the first time, what we believe to be the most parsimonious hypothesis of a single species. This null hypothesis remains to be more strongly tested when new evidence, such as distributional, behavioral, or genetic data, becomes available.

Both sexes of *A.arnacasericeella* comb. n. et stat. n. differ from the nominotypical subspecies in the following respects: DHW feeble pearly reflection restricted to posterior one-third of hindwing and not extending into discal cell; ventral ground color somewhat paler; discal band, postdiscal band, submarginal band on the ventral forewing and ventral hindwing narrower; ventral hindwing postdiscal band not bent inwards in cell Cu_2_ and gently curving towards inner margin (but see also below); ventral ocelli smaller (but see also below); bluish-lilac reflection on ventral surface appearing purplish.

#### Variation.

The ventral ocelli are variable in terms of size; while many specimens seem to possess ventral ocelli smaller than the nominotypical subspecies and *A.arnacaindianacristoi* ssp. n., some appear to have ocelli that are similar to the aforementioned two taxa in terms of size. The VHW postdiscal band is bent inwards in cell Cu_2_ in some specimens, whereas it gradually curves towards the inner margin in other specimens.

#### Distribution

(Fig. 6). This subspecies ranges from southern Mexico to Honduras, where it appears to be uncommon.

#### Examined specimens

(46 ♂, 23 ♀). See Appendix for the data of these specimens.

### Amiga arnaca indianacristoi

Taxon classificationAnimaliaNymphalidaeNymphalidae

Nakahara & Marín subsp. n.

http://zoobank.org/E2CB94D6-ED44-498C-BD23-313D5139601F

Figs 2o, p , 6 , 7

#### Description and diagnosis.

**MALE**: forewing length 18.5–21 mm (*n* = 6): Differs from the nominotypical subspecies in the following respects: dorsal hindwing bluish lilac reflection restricted to posterior one-thirds of hindwing and not extending into discal cell; ventral ground color appearing somewhat paler; discal band, postdiscal band, submarginal band on ventral forewing and ventral hindwing narrower; feeble pearly reflection on the ventral forewing (distal side of postdiscal band) absent or insignificant.

**FEMALE**: forewing length 20–22 mm (*n* = 3): Differs from the nominotypical subspecies by the characters provided for the male, except for the first DHW character.

#### Variation.

The area of the dorsal hindwing bluish-lilac reflection is variable, restricted only to the distal side of cells Cu_1_ and Cu_2_ in some specimens, whereas extending up to the area around M_3_ in some specimens. One female (FLMNH-MGCL 1036235) lacks the dorsal hindwing bluish-lilac reflection, while it is present in three other females (FLMNH-MGCL-264685, 1036240, 1036239).

#### Types.

**HOLOTYPE** ♂: // VENEZUELA: CARABOBO Hda. María Teresa (IAN), W of Las Trincheras 400 m, moist forest 28.vii.1981 Lee D. Miller, sta. VE16 // Allyn Museum Acc. 1981-23 // FLMNH-MGCL Specimen 264660 // (FLMNH).

**PARATYPES** (15 ♂, 4♀): **Venezuela**: *Aragua*: Rancho Grande Biological Station, [10°20'58"N,67°41'3"W], 1100 m, (Heppner, J. B.), Jul 1981, 1 ♀ [FLMNH-MGCL-1036240], (FLMNH); km 22 Maracay–Ocumaré rd., Rancho Grande, 1100–1200 m, (Lichy, R.), 2 Aug 1943, 1 ♂ [FLMNH-MGCL-262940], (FLMNH); Maracay, Pozo del Diablo, [10°17'N,67°37'W], 420–440 m, (Miller, L. D.), 22 Jul 1981, 1 ♂ [FLMNH-MGCL-264654], 1 ♂ [FLMNH-MGCL-264656], (FLMNH), 27 Jul 1981, 1 ♂ [FLMNH-MGCL-264655], (FLMNH); Maracay–Ocumare hwy., 1100–1200 m, (Lichy, R.), 4 Sept 1942, 1 ♀ [FLMNH-MGCL-1036235], (FLMNH); *Carabobo*: nr. Puerto Cabello, San Esteban, [10°26'N,68°1'W], (Hahnel de Sagan), 1^er^ trimestre 1877, 1 ♀ [BMNH(E)#1420075], (NHMUK), Jun, Jul 1877, 1 ♂ [BMNH(E)#1420068], 1 ♂ [BMNH(E)#1420069], (NHMUK); Yuma, [10°6'N,67°42'W], 550 m, (Miller, L. D.), 16 Dec 1981, 1 ♂ [FLMNH-MGCL-1036241], 1 ♀ [FLMNH-MGCL-1036239], (FLMNH); *Cojedes*: El Baul, Hato Pinero, (Brenner, J.), Feb 1968, 1 ♂ [FLMNH-MGCL-1036242], (FLMNH); *Distrito Federal*: Caracas, [10°30'N,66°55'W], 1200 m, (Forster, W.), 29 Jun 1954, 1 ♂, (ZSM); *Miranda*: 10 miles S of Caracas, [10°23'46"N,66°53'26"W], 1311 m, 30 Mar 1970, 1 ♂ [woods], (USNM); [Caracas], Macizo Naiguatá, 720–800 m, (Lichy, R.), 6 Dec 1942, 1 ♂ [FLMNH-MGCL-262941], (FLMNH); Massif du Naiguatá, (Lichy, R.), 5 Jul 1945, 1 ♂ [FLMNH-MGCL-264683], (FLMNH); mist forest 20 km SE Caracas, [10°21'24"N,66°46'18"W], 500–750 m, (Miller, L. D. & J. Y., Dukes, D.), 1 Sep 1990, 1 ♂ [FLMNH-MGCL-264657], 1 ♂ [FLMNH-MGCL-264659], (FLMNH); Río Chacaito, [10°25'N,66°55'W], 980–1080 m, (Lichy, R.), 12 Sep 1936, 1 ♂ [FLMNH-MGCL-264658], (FLMNH).

#### Etymology.

This new species-group name is proposed in recognition of our friend and colleague, Indiana Cristóbal Ríos-Málaver, known as “Indiana Cristo”, who studied the butterflies of the area where this taxon occurs. Indiana Cristo has contributed to Neotropical lepidopterology in various ways, especially through social media, where he is bringing lepidopterology to the public. This species-group name is treated as a latinized masculine noun in the genitive case.

#### Distribution

(Fig. 6). This taxon occurs in the Venezuelan Cordillera de la Costa and northwestern Cordillera de Mérida, and possibly also into the Serranía de Perijá.

#### Remarks.

We have examined a single specimen from Monagas, Venezuela (FLMNH-MGCL-264682), with a phenotype that corresponds to this subspecies. Thus, this taxon’s range may extend further east than that indicated above. However, given that we have examined only a single specimen from the area, combined with the fact that specimens from the island of Trinidad, whose butterfly fauna has strong biogeographic affinities with Monagas (e.g., Neild 1996), clearly represent the nominotypical subspecies, there still remains the possibility of this specimen being mislabeled.

**Figure 7.**
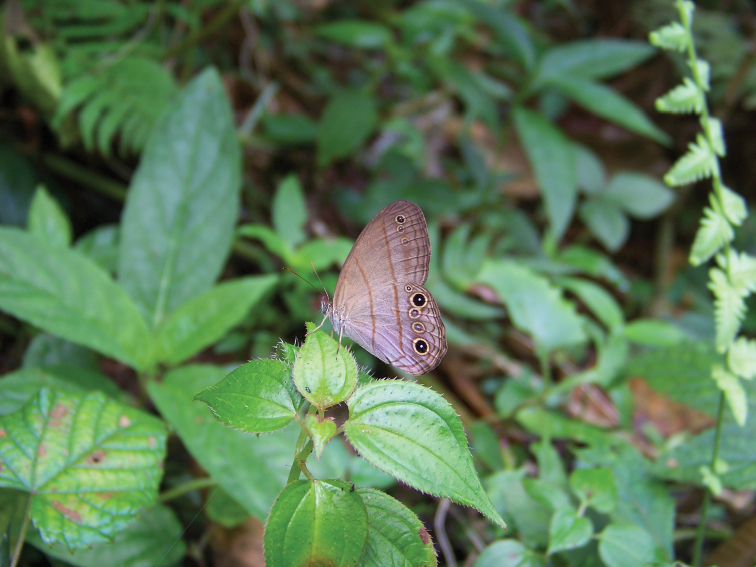
*Amigaarnacaindianacristoi* ssp. n. in nature, Altos de Pipe, Miranda, Venezuela (photographed by Indiana Cristóbal Ríos-Málaver, on 11 September 2011).

## Supplementary Material

XML Treatment for Amiga

XML Treatment for Amiga arnaca arnaca

XML Treatment for Amiga arnaca adela

XML Treatment for Amiga arnaca sericeella

XML Treatment for Amiga arnaca indianacristoi

## Appendix. Data of specimens examined in this study

**Abbreviations**: W&H = Willmott and Hall unpublished data; DABD = Darwin Andean Butterfly Database.

The locality of some specimens will appear the same as its state, province, department, etc., if no further information was available on the label.

***
Amiga arnaca arnaca
***

**Specimens examined (718 ♂, 207 ♀): Argentina**: *Entre Ríos*: Entre Ríos, (Smith, H. H.), Sep, 1 ♂ [BMNH(E)#1420202; Data unreliable. See B. M. 1915-3 K. J. Hayward MS'], (NHMUK). **Bolivia**: *Cochabamba*: Alto Palmar, [17°9'2"S,65°42'47"W], 1100 m, Nov 1960, 1 ♂, (ZSM); Valle del Sacta, [17°6'S,64°47'W], (Curso Mariposas Andinas), 12–16 Dec 2006, 1 ♂ [MHNNKM-15903], (MHNNKM); Yungas del Espíritu Santo, [17°6'S,65°40'W], (Germain, P.), 1888-1889, 1 ♂ [BMNH(E)#1420187], 1 ♂ [BMNH(E)#1420188], 1 ♀ [BMNH(E)#1420189], (NHMUK); *La Paz*: Madidi, [13°37'94"S,68°44'45"W], (Gareca, Y.), 23 Aug 2004, 1 ♂ [MUSM-LEP-100524], (MUSM); Río Zongo, [16°3'40"S,68°1'2"W], 1200 m, (Garlepp), 1895-1896, 2 ♂, (MNHU); Río Zongo, [16°3'40"S,68°1'2"W], 1200–1600 m, (Garlepp), 2 ♀, (MNHU); Torewa, [14°43'14"S,67°36'16"W], 245 m, (Apaza, M., Lipa, T.), 11–12 May 2004, 1 ♂ [MHNNKM-15902], (MHNNKM); Yungas de La Paz, [16°20'S,66°45'W], 1000 m, (Rolle), 1 ♀, (ZSM); *Pando*: Rutina, [12°16'49"S,68°31'43"W], 237 m, (Condori, J., Saire, A., Rojas, N.), 25 May 1999, 1 ♂ [MHNNKM-15901], (MHNNKM); *Santa Cruz*: Buenavista, [17°27'S,64°40'W], 750 m, (Steinbach, F.), Aug 1906–Apr 1907, 1 ♀ [BMNH(E)#1498940], (NHMUK); Santa Cruz de la Sierra, [17°48'S,63°10'W], (Steinbach, F.), Apr to beginning May 1904, 1 ♀ [BMNH(E)#1498939], (NHMUK), Feb 1904, 1 ♂ [BMNH(E)#1498937], (NHMUK), Feb 1909, 1 ♂ [BMNH(E)#1498938], (NHMUK); Sivingal, [18°2'42"S,64°2'32"W], 2026 m, 8 Mar 1991, 1 ♂ [MHNNKM-15904], (MHNNKM); *Not located*: ‘Bolivia’, 1 ♂ [BMNH(E)#1420190], 1 ♀ [BMNH(E)#1420191], (NHMUK). **Brazil**: *Acre*: 50 km NW Bujari, [9°32'53"S,68°18'9"W], 200 m, (DZUP); Cruzeiro do Sul, Pentecostes-Japim, [7°38'10"S,72°48'17"W], (DZUP); *Amapá*: Rio Preto, [1°90'S,51°21'W], 100 m, (Büche, M.), Dec 1996, 1 ♂ [MUSM-LEP-100522], (MUSM); *Amazonas*: Benjamin Constant, [4°23'S,70°2'W], (DZUP); Coari, [4°5'1"S,63°8'36"W], 35 m, (Hahnel), 1 ♂, (MNHU); Ega, [3°22'S,64°42'W], (Bates, H. W.), 1 ♂ [BMNH(E)#1420135], (NHMUK); Igarapé Massauari, [2°54'17"S,57°8'23"W], (Michael), 1892, 1 ♂, (MNHU); Manaus, [3°7'S,60°2'W], 1 ♂ [FLMNH-MGCL-263301], 1 ♂ [FLMNH-MGCL-263305], 1 ♂ [FLMNH-MGCL-263312], 1 ♂ [FLMNH-MGCL-263363], (FLMNH), (Hahnel), 1 ♂, (MNHU); Manaus, km 72 BR-174, Fazenda Dimona, [2°19'S,60°5'W], (Rogers, H., Sullivan, J.), Dec 1993, 1 ♂ [FLMNH-MGCL-207922], (FLMNH); Manicoré, [5°49'S,61°17'W], 1 ♀, (ZSM), Jun, 1 ♂, (ZSM), (Callaghan, C. J.), 16 Aug 1976, 1 ♂ [FLMNH-MGCL-263317], (FLMNH), (Hahnel), 1887, 1 ♂, 1 ♀, (MNHU); Nova Olinda do Norte, [3°45'24"S,59°2'54"W], Aug 1998, 1 ♂ [FLMNH-MGCL-264662], (FLMNH); nr. Benjamin Constant, Rio Javari, [4°23'S,70°2'W], (DZUP); Rio Japurá, Maraá, [1°50'S,65°22'W], Feb 1994, 1 ♂ [FLMNH-MGCL-207955], (FLMNH); Rio Purus, Ypiranga, [4°14'52"S,61°39'52"W], 30 m, 1 ♂ [FLMNH-MGCL-263313], (FLMNH); Rio Roosevelt, Apr 2006, 1 ♀ [FLMNH-MGCL-264653], (FLMNH); São Paulo de Olivença, [3°28'S,68°57'W], 1 ♂ [FLMNH-MGCL-263308], (FLMNH), (Mathan, M. de), 1 ♂ [BMNH(E)#1420129], 1 ♀ [BMNH(E)#1420133], (NHMUK), Jun, Jul 1883, 1 ♂ [BMNH(E)#1420130], 1 ♂ [BMNH(E)#1420131], 1 ♀ [BMNH(E)#1420132], (NHMUK), (Michael), 1891, 1 ♂, (MNHU), (Waehner, S.), Jan 1933, 1 ♂ [BMNH(E)#1497565], 1 ♂ [BMNH(E)#1497596], 1 ♂ [BMNH(E)#1497627], 1 ♂ [BMNH(E)#1497658], (NHMUK); Tefé, [3°22'S,64°44'W], (Hoffmanns, W.), Jun 1906, 1 ♂ [BMNH(E)#1498893], (NHMUK), (Mathan, M. de), Apr 1878–Jan 1879, 1 ♂ [BMNH(E)#1420137], (NHMUK), 3^er^ trimestre 1878, 1 ♂ [BMNH(E)#1420136], 1 ♂ [BMNH(E)#1498906], (NHMUK), Jan 1905, 1 ♂ [BMNH(E)#1498891], (NHMUK), Nov 1907, 1 ♂ [BMNH(E)#1498892], (NHMUK); Tonantins, [2°52'S,67°48'W], Mar 1993, 1 ♀ [FLMNH-MGCL-207957], (FLMNH); *Bahía*: Bahía, [12°59'S,38°31'W], 1 ♂ [BMNH(E)#1420141], (NHMUK); Cachimbo, 1890, 1 ♂ [BMNH(E)#1420138], 1 ♂ [BMNH(E)#1420139], 1 ♂ [BMNH(E)#1420140], (NHMUK); Camacã, [15°24'S,39°29'W], (DZUP); Camacã, Serra Bonita, (Freitas, A. V. L., Carreira, J. Y. O.), 18-20 Jul 2014, 1 ♂, (ZUEC); Ilhéus, [14°46'S,39°3'W], (DZUP); Itabuna, [14°47'S,39°17'W], (Becker, V.), 18 Jan 1967, 1 ♂ [FLMNH-MGCL-263302], 1 ♂ [FLMNH-MGCL-263303], 1 ♂ [FLMNH-MGCL-295863], (FLMNH); Rio das Contas, Jitaúna, [14°1'S,39°57'W], 150 m, (DZUP); Ubatã, 100 m, (Brown, K. S.), 21 Dec 1966, 1 ♂ [FLMNH-MGCL-263299], 1 ♀ [FLMNH-MGCL-263339], (FLMNH); Xerm, [22°33'S,43°17'W], (DZUP); *Espírito Santo*: Conceição da Barra, [18°34'S,39°45'W], (DZUP), 25 Sep 1969, 1 ♀, (ZSM); Espírito Santo, [20°20'S,40°17'W], 1 ♂ [BMNH(E)#1420142], 1 ♂ [BMNH(E)#1420143], 1 ♂ [BMNH(E)#1420144], 1 ♀ [BMNH(E)#1420160], 1 ♀ [BMNH(E)#1420166], (NHMUK); Linhares, [19°23'S,40°4'W], (DZUP), (Elias, P. C.), Aug 1972, 1 ♂ [FLMNH-MGCL-262993], 1 ♂ [FLMNH-MGCL-263008], 1 ♂ [FLMNH-MGCL-263019], 1 ♂ [FLMNH-MGCL-263024], (FLMNH), Feb 1972, 1 ♂ [FLMNH-MGCL-262991], 1 ♂ [FLMNH-MGCL-262992], 1 ♂ [FLMNH-MGCL-262998], 1 ♂ [FLMNH-MGCL-262999], 1 ♂ [FLMNH-MGCL-263000], 1 ♂ [FLMNH-MGCL-263001], 1 ♂ [FLMNH-MGCL-263002], 1 ♂ [FLMNH-MGCL-263005], 1 ♂ [FLMNH-MGCL-263006], 1 ♂ [FLMNH-MGCL-263009], 1 ♂ [FLMNH-MGCL-263010], 1 ♂ [FLMNH-MGCL-263017], 1 ♂ [FLMNH-MGCL-263018], 1 ♂ [FLMNH-MGCL-263023], 1 ♀ [FLMNH-MGCL-263034], 1 ♀ [FLMNH-MGCL-263035], (FLMNH), Jul 1972, 1 ♂ [FLMNH-MGCL-263013], 1 ♀ [FLMNH-MGCL-263043], (FLMNH), Mar 1972, 1 ♂ [FLMNH-MGCL-262986], 1 ♂ [FLMNH-MGCL-262988], 1 ♂ [FLMNH-MGCL-262989], 1 ♂ [FLMNH-MGCL-262990], 1 ♂ [FLMNH-MGCL-262995], 1 ♂ [FLMNH-MGCL-262996], 1 ♂ [FLMNH-MGCL-262997], 1 ♂ [FLMNH-MGCL-263003], 1 ♂ [FLMNH-MGCL-263004], 1 ♂ [FLMNH-MGCL-263007], 1 ♂ [FLMNH-MGCL-263011], 1 ♂ [FLMNH-MGCL-263012], 1 ♂ [FLMNH-MGCL-263014], 1 ♂ [FLMNH-MGCL-263015], 1 ♂ [FLMNH-MGCL-263020], 1 ♂ [FLMNH-MGCL-263022], 1 ♂ [FLMNH-MGCL-263025], 1 ♀ [FLMNH-MGCL-263036], 1 ♀ [FLMNH-MGCL-263037], 1 ♀ [FLMNH-MGCL-263038], 1 ♀ [FLMNH-MGCL-263040], 1 ♀ [FLMNH-MGCL-263041], 1 ♀ [FLMNH-MGCL-263042], 1 ♀ [FLMNH-MGCL-263044], 1 ♀ [FLMNH-MGCL-263045], 1 ♀ [FLMNH-MGCL-263046], (FLMNH), May 1972, 1 ♂ [FLMNH-MGCL-262982], 1 ♂ [FLMNH-MGCL-262983], 1 ♂ [FLMNH-MGCL-262984], 1 ♂ [FLMNH-MGCL-262985], 1 ♂ [FLMNH-MGCL-262987], 1 ♂ [FLMNH-MGCL-262994], 1 ♂ [FLMNH-MGCL-263016], 1 ♂ [FLMNH-MGCL-263021], 1 ♀ [FLMNH-MGCL-263047], (FLMNH), Sep 1972, 1 ♀ [FLMNH-MGCL-263039], (FLMNH), May 1972, 1 ♂ [FLMNH-MGCL-262981], (FLMNH); Reserva Ecológica de Sooretama, [19°3'25"S,40°8'50"W], 100 m, (DZUP), (Mielke, O. H. H., Casagrande, M. M.), 21-25 Jan 2014, 1 ♀, (DZUP); Santa Cruz, [19°57'S,40°9'W], (Callaghan, C. J.), 19 Jun 1976, 1 ♂/♀ [FLMNH-MGCL-207921], (FLMNH); Santa Leopoldina, [20°6'S,40°32'W], (DZUP); Santa Teresa, [19°56'S,40°36'W], (DZUP); *Goiás*: Goiás Velho, (DZUP); Ilha do Bananal, [8°35'S,49°28'W], (DZUP); N. Anápolis, Belo Horizonte-Brasília, km 47, (Callaghan, C. J.), 10 Apr 1973, 1 ♂ [FLMNH-MGCL-263029], (FLMNH); *Maranhão*: 26 km E Feira Nova do Maranhão, Fazenda Forquilha dos Brejos, [7°0'29"S,46°26'30"W], (DZUP), (Mielke, O. H. H.), 15-25 Feb 2012, 1 ♀, (DZUP); 30 km W Fortaleza dos Nogueiras, Retiro, 470 m, (DZUP); Imperatriz, (DZUP); km 108 Açailandia-Sta. Luzia, Fazenda Terrasse, (DZUP); *Mato Grosso*: 10 mi. S. Diamantino, Melguira, [14°32'S,56°19'W], 610 m, (Collenette, C. L.), 23 May–3 Jun 1927, 1 ♂ [BMNH(E)#1420156], (NHMUK); Barra do Garça, (DZUP); Cáceres, Coronel Rio Branco, Rio Vermelho, [15°9'S,58°15'W], 400 m, (DZUP); Diamantino, Alto Rio Arinos, Faz.[enda] S.[ão] João, [14°21'18"S,56°9'2"W], (DZUP); nr. Melguira, Alto Rio Paraguai, Barra do Bugres, [15°4'S,57°10'W], 150 m, (DZUP); no specific locality, (Germain, P.), 1886, 1 ♂ [BMNH(E)#1420155], (NHMUK); *Minas Gerais*: Belo-Brasilia, woods along river, 47 km N Anápolis, (Callaghan, C. J.), 10 Apr 1973, 1 ♂ [FLMNH-MGCL-263311], 1 ♂ [FLMNH-MGCL-263319], 1 ♂ [FLMNH-MGCL-263323], 1 ♂ [FLMNH-MGCL-263327], 1 ♂ [FLMNH-MGCL-263328], 1 ♂ [FLMNH-MGCL-263335], (FLMNH), 10 Apr 1973, 1 ♀ [FLMNH-MGCL-1036236], (FLMNH); Caratinga, Estação Biológica Caratinga, [19°47'S,42°8'W], 400 m, (DZUP); Conceição dos Ouros, Serra Grande, [22°25'S,45°47'W], 1365 m, (Melo, D.), 19 Nov 2012, 1 ♂, (ZUEC); nr. Col. Fabriciano, Parque Estadual do Rio Doce, (Callaghan, C. J.), 25 Jul 1972, 1 ♂ [FLMNH-MGCL-263031], 1 ♂ [FLMNH-MGCL-263032], (FLMNH), 25-28 Jul 1972, 1 ♂ [FLMNH-MGCL-263030; in recently burned forest area], (FLMNH); Parque Estadual do Rio Doce, [20°15'S,42°54'W], 200 m, (DZUP), (Callaghan, C. J.), 26 Jul 1972, 1 ♂ [FLMNH-MGCL-263310], (FLMNH); Teófilo Otoni, San Jacinto Valley, Apr 1908, 1 ♀ [BMNH(E)#1498895], 1 ♀ [BMNH(E)#1498898], (NHMUK), Feb 1908, 1 ♂ [BMNH(E)#1498889], 1 ♀ [BMNH(E)#1498897], (NHMUK), Jul 1908, 1 ♂ [BMNH(E)#1498890], (NHMUK), Mar 1908, 1 ♀ [BMNH(E)#1498894], (NHMUK), May 1908, 1 ♂ [BMNH(E)#1498887], 1 ♂ [BMNH(E)#1498888], (NHMUK), Nov 1907, 1 ♀ [BMNH(E)#1498896], (NHMUK); no specific locality, 1 ♂ [BMNH(E)#1420157], 1 ♀ [BMNH(E)#1420164], (NHMUK), (Callaghan, C. J.), 10 Apr 1973, 1 ♂ [FLMNH-MGCL-1036218], (FLMNH); *Pará*: [Rio] Tapájos, [4°16'8"S,55°59'10"W], 25 m, 3 Dec 2012, 1 ♂, (ZUEC); Pará, (Bates, H. W.), 1 ♂ [BMNH(E)#1420119], 1 ♂ [BMNH(E)#1420120], 1 ♀ [BMNH(E)#1420123], 1 ♀ [BMNH(E)#1420124], (NHMUK), (Smith, H. H.), Aug, 1 ♂ [BMNH(E)#1420122], (NHMUK), (Wallace, A. R.), 1 ♂ [BMNH(E)#1420121], (NHMUK); 15 km S Itaituba, [4°17'S,56°5'W], (Callaghan, C. J.), 23 Jul 1978, 1 ♂ [FLMNH-MGCL-263027], 1 ♂ [FLMNH-MGCL-263028], (FLMNH); Água Azul, (DZUP); Belém, [1°27'S,48°29'W], (Forster, W.), 15 Jun 1954, 1 ♀, (ZSM), 16 Jun 1954, 2 ♂, (ZSM), (Kesselring, J.), 4 Feb 1971, 1 ♂ [FLMNH-MGCL-263333], (FLMNH); Belém, Utinga, [1°27'S,48°25'W], (DZUP); Benevides, Neopolis, [1°22'S,48°15'W], (DZUP), (Carneiro, E., Dolibaina, D., Dias, F., Moreira), 2 Feb 2010, 1 ♀, (DZUP); Bragança, [1°3'S,46°47'W], (Mathan, M. de), 1 ♂ [BMNH(E)#1420127], (NHMUK); Ilha da Maracá, Alto Alegre, (DZUP); Itaituba on Río Tapajós, [4°16'8"S,55°59'10"W], Aug, 1 ♂ [FLMNH-MGCL-263306], 1 ♂ [FLMNH-MGCL-263307], (FLMNH); Itaituba, [4°17'S,55°59'W], (Michael), 1 ♂, (MNHU), 1890, 1 ♀, (MNHU); Maçauari-Belém, (DZUP); nr. Belém, Fazenda Velha, [1°20'S,48°14'W], (Callaghan, C. J.), 18 Nov 1973, 1 ♂ [FLMNH-MGCL-263304], 1 ♂ [FLMNH-MGCL-263326], 1 ♂ [FLMNH-MGCL-263329], 1 ♀ [FLMNH-MGCL-263338], (FLMNH), 19 Nov 1973, 1 ♂ [FLMNH-MGCL-263314], 1 ♂ [FLMNH-MGCL-263315], 1 ♂ [FLMNH-MGCL-263316], 1 ♂ [FLMNH-MGCL-263318], (FLMNH); Óbidos, [1°54'S,55°31'W], (DZUP); Pará, [1°0'S,51°11'W], 2 ♂, (MNHU), (Goeldi), 1 ♂ [BMNH(E)#1420114], 1 ♂ [BMNH(E)#1420115], (NHMUK), (Hahnel), 1 ♂, (MNHU), (Mathan, M. de), 1 ♂ [BMNH(E)#1420116], 1 ♂ [BMNH(E)#1420117], 1 ♂ [BMNH(E)#1420118], (NHMUK), (Moss, A. M.), 1 ♂ [BMNH(E)#1498899], 1 ♂ [BMNH(E)#1498900], 1 ♀ [BMNH(E)#1498901], (NHMUK), (Stevens, M.), 1 ♂ [BMNH(E)#1420113], (NHMUK); Pará, [1°0'S,51°11'W], 229 m, (Majerus, M.), 1 Mar 2007, 1 ♂ [BMNH(E)#1267133], (NHMUK); 'Raisao', 1 ♂ [FLMNH-MGCL-263298], (FLMNH); Rio Cuminá, [1°22'S,56°3'W], (DZUP); Rio Cuminá, Cachoeira Breu, [0°27'S,56°5'W], (DZUP); Santarém, [2°26'S,54°43'W], 1 ♂ [FLMNH-MGCL-263300], (FLMNH), (Smith, H. H.), 1 ♂ [BMNH(E)#1420134], (NHMUK); Santo Antônio do Tauá, Reserva Sonho Azul, [1°15'S,48°3'W], (DZUP); Taperinha, [0°52'S,47°49'W], (Boy, H. C.), Aug 1920, 1 ♂, (ZSM), Sep 1920, 4 ♂, (ZSM); Urumanza, 22/07/59, 1 ♂ [BMNH(E)#1670216], (NHMUK); *Paraná*: Alto da Serra, Morretes, [25°28'S,48°49'W], 850 m, (DZUP); *Pernambuco*: Jaqueira, RPPN Frei Caneca, [8°43'16"S,35°50'W], 650 m, (DZUP); Pernambuco, [8°47'S,37°42'W], 1 ♂ [BMNH(E)#1420128], (NHMUK); *Rio de Janeiro*: Duque de Caxias, Villa Equitativa, 100 m, (Callaghan, C. J.), 1 ♂ [FLMNH-MGCL-1036210], 1 ♂ [FLMNH-MGCL-1036211], 1 ♂ [FLMNH-MGCL-1036212], 1 ♂ [FLMNH-MGCL-1036213], (FLMNH); Guapimirim, [22°32'S,42°59'W], 400 m, (Callaghan, C. J.), 27 May 1972, 1 ♂ [FLMNH-MGCL-262974], (FLMNH); Guapimirim, [22°32'S,42°59'W], 50 m, (DZUP), (Callaghan, C. J.), 3 Sep 1971, 1 ♂ [FLMNH-MGCL-262972], 1 ♂ [FLMNH-MGCL-262973], (FLMNH); Imbariê, [22°38'S,43°13'W], 25 m, (DZUP), (Callaghan, C. J.), Jun 1976, 1 ♂ [FLMNH-MGCL-207914], 1 ♂ [FLMNH-MGCL-207915], 1 ♂ [FLMNH-MGCL-207916], (FLMNH); km 16 Rio Teresópolis, (Callaghan, C. J.), 10 May 1971, 1 ♂ [FLMNH-MGCL-262975], (FLMNH), 10 Oct 1971, 1 ♂ [FLMNH-MGCL-262977], (FLMNH), 5 Jun 1972, 1 ♂ [FLMNH-MGCL-263026], (FLMNH), 6 May 1972, 1 ♀ [FLMNH-MGCL-263048], (FLMNH); km 27 Rio Teresópolis, (Callaghan, C. J.), 24 Jun 1971, 1 ♂ [FLMNH-MGCL-262968], (FLMNH), 6 Sep 1971, 1 ♂ [FLMNH-MGCL-262976], (FLMNH); km 42 Rio de Janeiro-Teresópolis rd., Mt. Olivetti, [22°31'31"S,42°59'54"W], (Callaghan, C. J.), 2 Jul 1972, 1 ♀ [FLMNH-MGCL-263049], (FLMNH); Laguna de Sacuarema, [22°56'S,42°28'W], (Germain, P.), Sep 1884, 1 ♂ [BMNH(E)#1420145], 1 ♂ [BMNH(E)#1420146], 1 ♂ [BMNH(E)#1420147], 1 ♂ [BMNH(E)#1420148], 1 ♂ [BMNH(E)#1420149], 1 ♂ [BMNH(E)#1420150], 1 ♂ [BMNH(E)#1420151], 1 ♂ [BMNH(E)#1420152], 1 ♂ [BMNH(E)#1420153], 1 ♀ [BMNH(E)#1420161], 1 ♀ [BMNH(E)#1420162], 1 ♀ [BMNH(E)#1420163], (NHMUK); Petrópolis to Rio de Janeiro old rd., 300 m, (Callaghan, C. J.), 13 May 1972, 1 ♂ [FLMNH-MGCL-262967], 1 ♂ [FLMNH-MGCL-262978], 1 ♂ [FLMNH-MGCL-262979], 1 ♂ [FLMNH-MGCL-262980], (FLMNH); 'Rio de Janeiro Zubeaida', 25 m, (Ebert, H.), 10 Feb 1975, 1 ♂ [FLMNH-MGCL-207917], 1 ♂ [FLMNH-MGCL-207918], 1 ♂ [FLMNH-MGCL-207919], 1 ♂ [FLMNH-MGCL-207920], (FLMNH); Rio de Janeiro, [22°55'S,43°14'W], 1 ♂ [BMNH(E)#1420154], (NHMUK); Rio-Petrópolis Hwy. km 20, Magé, (Callaghan, C. J.), 2 May 1971, 1 ♂ [FLMNH-MGCL-262969], (FLMNH); Serra de Tingua, Xerém, [22°32'S,43°18'W], (Brown, K. S.), 21 Mar 1967, 1 ♂ [FLMNH-MGCL-262970], 1 ♂ [FLMNH-MGCL-262971], (FLMNH); Silvia Jardim, (Callaghan, C. J.), 8 Jul 1975, 1 ♂ [FLMNH-MGCL-263033], (FLMNH); Três Rios, Jacarepaguá, 1 ♂ [HT *priamis*: 'No 5630'//'Euptychia ar-naea priamis d' Alm 1992 Almeida det. ♂'//'Coll. D' Almeida'//'HOLOTIPO'//Jacarepaguá Três-Rios. Rio, 19.. ♂']; Vila Inhomirim, [22°34'5"S,43°10'35"W], 180 m, (DZUP); *Rondônia*: C -20 5 km N, 3 km W Cacaulândia, Fazenda Rancho Grande, Lot 25 forest trail, (Eiler, D. L.), 13 Nov 1992, 1 ♂ [FLMNH-MGCL-264636], (FLMNH), 20 Nov 1992, 1 ♂ [FLMNH-MGCL-264642], (FLMNH), 21 Nov 1992, 1 ♂ [FLMNH-MGCL-264635], (FLMNH), (Emmel, T. C.), 6 Nov 1989, 1 ♂ [FLMNH-MGCL-207970], 1 ♂ [FLMNH-MGCL-207971], (FLMNH), (Groce, L. L.), 22 Mar 1991, 1 ♂ [FLMNH-MGCL-207972], (FLMNH); 1 km N Cacaulândia, [10°31'30"S,62°48'W], (Austin, G. T.), 13 Nov 1992, 1 ♀ [FLMNH-MGCL-207956], (FLMNH); 1 km N Cacaulândia, Fernandes Trail (linea C-14) off B-65, [10°19'33"S,62°54'W], (Austin, G. T.), 10 Nov 1990, 1 ♂ [FLMNH-MGCL-1036222], (FLMNH), 8 Dec 1990, 1 ♂ [FLMNH-MGCL-207952], 1 ♂ [FLMNH-MGCL-207953], (FLMNH); 10 km S Cacaulândia, [10°37'24"S,62°48'W], (Austin, G. T.), 9 Dec 1990, 1 ♀ [FLMNH-MGCL-207948], (FLMNH); 12.5 km S Cacaulândia, [10°39'S,62°48'W], (Austin, G. T.), 12 Dec 1990, 1 ♂ [FLMNH-MGCL-207945], (FLMNH), 21 Mar 1991, 1 ♀ [FLMNH-MGCL-207950], (FLMNH), 3 Nov 1990, 1 ♂ [FLMNH-MGCL-207944], (FLMNH), 9 Dec 1990, 1 ♂ [FLMNH-MGCL-207946], 1 ♂ [FLMNH-MGCL-207947], (FLMNH); 15 km S Cacaulândia, [10°40'S,62°48'W], (Austin, G. T.), 22 Apr 1991, 1 ♀ [FLMNH-MGCL-207949], (FLMNH); 62 km S Ariquemes, Fazenda Rancho Grande, linea C-20, 7 km E B-65, [10°17'58"S,62°52'14"W], 170 m, (Austin, G. T.), 16 Sep 1992, 1 ♂ [FLMNH-MGCL-207958], (FLMNH), 20 Apr 1991, 1 ♂ [FLMNH-MGCL-1036223], (FLMNH), 22 Nov 1991, 1 ♂ [FLMNH-MGCL-207959], (FLMNH), 30 Oct 1990, 1 ♂ [FLMNH-MGCL-207962], (FLMNH), 5 Nov 1991, 1 ♂ [FLMNH-MGCL-207960], (FLMNH), 9 Nov 1990, 1 ♂ [FLMNH-MGCL-207961], (FLMNH), (Bongiolo, G.), 24 Mar 1991, 1 ♂ [FLMNH-MGCL-207963], (FLMNH); 67 km S Ariquemes, 5 km S of Cacaulândia on linha C-10 at Rio Pardo off B-65, [10°23'15"S,62°54'53"W], (Gomes, O.), 5 Sep 1993, 1 ♂ [FLMNH-MGCL-207951], (FLMNH), 7 Dec 1995, 1 ♂ [FLMNH-MGCL-264644], (FLMNH); Ariquemes 70 km S, B-80 between lineas C-10 and 15, [10°24'36"S,62°53'59"W], (Austin, G. T.), 8 Nov 1991, 1 ♂/♀ [FLMNH-MGCL-207943], (FLMNH); Ariquemes, [9°56'S,63°3'W], (DZUP); Fazenda Rancho Grande (Ariquemes 62 km S linha C-20, 7 km E B-65), [10°32'S,62°48'W], (Austin, G. T.), 11 Jun 1993, 1 ♀ [FLMNH-MGCL-207932], (FLMNH), 13 Nov 1992, 1 ♂ [FLMNH-MGCL-207930], (FLMNH), 14 Apr 1992, 1 ♂ [FLMNH-MGCL-207924], (FLMNH), 17 Sep 1992, 1 ♀ [FLMNH-MGCL-207933], (FLMNH), 19 Sep 1992, 1 ♀ [FLMNH-MGCL-207931], (FLMNH), 20 Apr 1991, 1 ♂ [FLMNH-MGCL-207926; station #2 (forest)], (FLMNH), 20 Nov 1991, 1 ♂ [FLMNH-MGCL-207928], (FLMNH), 20 Sep 1992, 1 ♂ [FLMNH-MGCL-207929], (FLMNH), 25 Oct 1992, 1 ♂ [FLMNH-MGCL-207927], (FLMNH), 30 Oct 1990, 1 ♂ [FLMNH-MGCL-207925], (FLMNH), 31 Oct 1992, 1 ♀ [FLMNH-MGCL-207934; station #6 forest], (FLMNH), 7 Dec 1990, 1 ♀ [FLMNH-MGCL-207935], (FLMNH), 8 Apr 1995, 1 ♂ [FLMNH-MGCL-264639; Trap #3], (FLMNH), (Bongiolo, G.), 24 Mar 1991, 1 ♂ [FLMNH-MGCL-207923], (FLMNH); Fazenda Rancho Grande (Ariquemes 62 km S C-20), [10°32'S,62°48'W], 165 m, (Harris, L. & H.), 17 April 1992 1 ♀ [FLMNH-MGCL-207984], (FLMNH); Fazenda Rancho Grande (Ariquemes 62 km S linha C-20, 7 km E B-65), [10°32'S,62°48'W], 143 m, (Harris, L. & H.), 20 Apr 1997, 1 ♂ [FLMNH-MGCL-264640], 1 ♂ [FLMNH-MGCL-264641], 1 ♂ [FLMNH-MGCL-264651], (FLMNH); Fazenda Rancho Grande (Ariquemes 62 km S linha C-20, 7 km E B-65), [10°32'S,62°48'W], 180 m, (Austin, G. T.), 21 Mar 1989, 1 ♂ [FLMNH-MGCL-263334], (FLMNH); Fazenda Rancho Grande (Ariquemes 65 km S linha C-20, 10 km E B-65, 3 km E Fazenda RG lot 18), (Harris, L. & C.), 12 Apr 1992, 1 ♂ [FLMNH-MGCL-207936], (FLMNH), 17 Apr 1992, 1 ♂ [FLMNH-MGCL-207937], (FLMNH), 21 Apr 1992, 1 ♂ [FLMNH-MGCL-207938], (FLMNH); Jaru, [10°27'S,62°30'W], (DZUP); Linea C-20 off B-65 at Rio Pardo, Fernandes Trail, (Austin, G. T.), 10 Dec 1990, 1 ♂ [FLMNH-MGCL-207954], (FLMNH); nr. Cacaulândia, 62 km. S. Ariquemes, Fazenda Rancho Grande, Linea C-20, 7 km E B-65, 20 Apr 1997, 1 ♂ [FLMNH-MGCL-207939], (FLMNH), 20 Apr 1997, 1 ♂ [FLMNH-MGCL-207940], 1 ♂ [FLMNH-MGCL-207941], 1 ♂ [FLMNH-MGCL-207942], (FLMNH), (Harris, L.), 20 Apr 1997, 1 ♂ [FLMNH-MGCL-1036229], (FLMNH); nr. Cacaulândia, 62 km. S. Ariquemes, Fazenda Rancho Grande, Linea C-20, 7 km E B-65, (Ross, G.), 13 Jan 1991, 1 ♂ [FLMNH-MGCL-207969], (FLMNH), 24 Jan 1991, 1 ♂ [FLMNH-MGCL-207968], (FLMNH), 29 Dec 1990, 1 ♀ [FLMNH-MGCL-207967], (FLMNH), 31 Dec 1991, 1 ♂ [FLMNH-MGCL-207965], (FLMNH), 4 Feb 1991, 1 ♂ [FLMNH-MGCL-207964], (FLMNH), 5 Feb 1991, 1 ♀ [FLMNH-MGCL-207966], (FLMNH), (Smith, J. A.), 18-26 Apr 1991, 1 ♂, (BME); nr. Cacaulândia, 62 km. S. Ariquemes, Fazendo Rancho Grande, [10°32'S,62°48'W], 180-200 m, (Heydon, S. L.), 4 Dec 1991, 1 ♂ [vapor headlamp], (BME); nr. Cacaulândia, 62 km. S. Ariquemes, linea C10, Fazenda Rancho Grande, [10°32'S,62°48'W], 180-200 m, (Austin, G. T.), 31 Oct 1989, 1 ♂ [FLMNH-MGCL-207973], 1 ♂ [FLMNH-MGCL-207974], (FLMNH); vicinity of Fazenda Rancho Grande, 62 km S Ariquemes off B-65, [10°17'58"S,62°52'14"W], 165 m, (Harris, L. & C.), 14 Apr 1992, 1 ♂ [FLMNH-MGCL-264646], (FLMNH); vicinity of Fazenda Rancho Grande, 62 km S Ariquemes off B-65, [10°17'58"S,62°52'14"W], 180 m, (Austin, G. T.), 24 Oct 1989, 1 ♂ [FLMNH-MGCL-263309], (FLMNH); *Roraima*: Amajari, Tepequén, 620 m, (DZUP); Maloca da Serra do Sul, (DZUP); Pacaraima, [4°27'57"S,61°8'32"W], 800 m, (DZUP); Surucucu, (DZUP); *São Paulo*: Ariri, (DZUP); *Not located*: ‘Brazil’, 1 ♂ [BMNH(E)#1420158], 1 ♂ [BMNH(E)#1420159], 1 ♀ [BMNH(E)#1420167], (NHMUK), (Camalte, B. D.), 1 ♀ [BMNH(E)#1420165], (NHMUK); Rio Tapajos, Barreiras, Oct, 2 ♂, (ZSM). **Colombia**: *Amazonas*: Río Caquetá, La Pedrera, [1°18'S,69°42'W], 170 m, May 1992, 1 ♂ [FLMNH-MGCL-257060], (FLMNH), Apr 1992, 1 ♂ [FLMNH-MGCL-257061], (FLMNH), (Simon, M. J.), 18 May 1992, 1 ♂ [FLMNH-MGCL-257064], 1 ♂ [FLMNH-MGCL-257068], (FLMNH), 25 May 1992, 1 ♂ [FLMNH-MGCL-257063], (FLMNH), 29 May 1992, 1 ♂ [FLMNH-MGCL-257062], (FLMNH), 7 May 1992, 1 ♂ [FLMNH-MGCL-257067], 1 ♂ [FLMNH-MGCL-257069], (FLMNH); Río San Miguel, Santa Rosa de Sucumbíos, (Simon, M. J.), 9 Sep 1971, 1 ♂ [FLMNH-MGCL-263103], (FLMNH); *Caquetá*: Montañita, [1°25'N,75°28'W], 366 m, (Nicolay, S. S.), 24 Jan 1971, 1 ♀ [FLMNH-MGCL-263133], (FLMNH), 25 Jan 1971, 1 ♂ [FLMNH-MGCL-263101], (FLMNH); *Meta*: Bosque Bavaria, w Villavicencio-Restrepo, [4°11'N,73°39'46"W], 600 m, (Lamas, G.), 12-13 Oct 2006, 1 ♂ [MUSM-LEP-100439], (MUSM); Cuchilla, 7 Sep 1943, 1 ♂ [FLMNH-MGCL-263106], (FLMNH); Río Guayabero, [2°15'N,73°45'W], 300 m, (Trapp, D.), 15 Jan 1959, 1 ♀ ['Macarena Sur'], (ZSM); Río Negro, [4°12'N,73°42'23"W], 732 m, (Nicolay, S. S.), 16 Jan 1971, 1 ♀ [FLMNH-MGCL-263128], (FLMNH); Villavicencio, [4°9'N,73°38'W], 500 m, (Robbins, R. K.), 9 Jul 1972, 1 ♂ [FLMNH-MGCL-263100], (FLMNH); *Putumayo*: Mocoa, [1°9'N,76°37'W], 650 m, (Sullivan, J. B.), 2 Jul 1981, 1 ♂ [FLMNH-MGCL-264643], (FLMNH); Orito, [0°17'N,76°53'W], 400 m, (Sullivan, J. B.), 3 Jul 1981, 1 ♂ [FLMNH-MGCL-264637], 1 ♂ [FLMNH-MGCL-264638], (FLMNH); Río Caqueta, Curillo, [1°0'N,76°0'w], 200 m, (Mallet, J., Jackson, D., Garcia C., P.), 1-7 Aug 1977, 1 ♂ [FLMNH-MGCL-264650], (FLMNH); Villa Garzón, [1°0'N,76°30'w], 300 m, (Mallet, J.), 18 Jun 1977, 1 ♂ [BMNH(E)#1498967], (NHMUK), (Mallet, J., Jackson, D., Garcia C., P.), 2 Feb 1977, 1 ♀ [FLMNH-MGCL-264652], (FLMNH), 2 Jul 1977, 1 ♂ [FLMNH-MGCL-264649], (FLMNH); *Valle del Cauca*: Anchicayá, [3°34'27"N,76°46'W], 305 m, (Sullivan, J. B.), 24 Jan 1988, 1 ♂ [FLMNH-MGCL-257054], (FLMNH); *Not located*: ‘Colombia’, (Ovalle, F.), 1 ♂ [FLMNH-MGCL-263102], (FLMNH). **Ecuador**: *Azuay*: 'Sigsig' – (error), (Lafebre, R. de), Jul 1970, 1 ♂ [FLMNH-MGCL-263349], 1 ♂ [FLMNH-MGCL-263355], 1 ♀ [FLMNH-MGCL-263370], (FLMNH), Jul 1970, 1 ♂ [FLMNH-MGCL-263343], (FLMNH); *Loja*: 'environs of Loja' – (error), 1886, 1 ♀ [BMNH(E)#1420066], (NHMUK); 'Loja' – (error), 1800 m, 1893, 1 ♂ [BMNH(E)#1498859], (NHMUK), (Gaujon), 1886, 1 ♂ [BMNH(E)#1498856], 1 ♂ [BMNH(E)#1498857], (NHMUK); 'Vilcabamba' – (error), (Lafebre, R. de), Jul 1970, 1 ♂ [FLMNH-MGCL-263350], 1 ♀ [FLMNH-MGCL-263374], (FLMNH); *Morona-Santiago*: 2 km N San Isidro, [2°11'54"S,78°9'24"W], 1250–1450 m, (Busby, R. C.), 10 Jan 2015, 1 ♂ [FLMNH-MGCL-195469], 1 ♂ [FLMNH-MGCL-195476], (FLMNH); E of Misión de Bomboiza, [3°26'6"S,78°30'18"W], 950–1300 m, (Busby, R. C.), 18 Sep 2014, 1 ♂ [FLMNH-MGCL-195374], (FLMNH); forest ridge nr. Yaupi, [2°51'15"S,77°56'48"W], 400 m, (Gallice, G.), 13 Jun 2009, 1 ♂ [FLMNH-MGCL-152428], (FLMNH), 16 Jun 2009, 1 ♂ [FLMNH-MGCL-152426], (FLMNH), 21 Jun 2009, 1 ♂ [FLMNH-MGCL-152429], (FLMNH), 4 Jul 2009, 1 ♂ [FLMNH-MGCL-152430], (FLMNH); Gualaquiza, Río Bomboiza, [3°26'S,78°32'W], 850 m, (Hall, J. P. W., Willmott, K. R.), 26–29 Jul 1993, 1 ♀ [FLMNH-MGCL-257138], (FLMNH); hill N of Santiago, [3°2'32"S,78°0'20"W], 350 m, (Busby, R. C.), 15 Sep 2014, 1 ♂ [FLMNH-MGCL-195371], (FLMNH); hill N of Santiago, [3°2'32"S,78°0'20"W], 380 m, (Hall, J. P. W., Willmott, K. R., J. C. R., J. I. R), 29 Jul, 2 Aug 2016, (INABIO); km 14 Chigüinda–Gualaquiza rd., [3°15'45"S,78°39'4"W], 1300 m, (Willmott, K. R., J. I. R., J. C. R.), 15 Jun 2013, (INABIO); km 14 Chigüinda–Gualaquiza rd., [3°15'45"S,78°39'4"W], 1300-1600 m, (Busby, R. C.), 17 Sep 2014, 1 ♂ [FLMNH-MGCL-195373], (FLMNH); km 2 Santiago–Puerto Morona rd., [3°2'8"S,78°0'W], 350-500 m, (Ahrenholz, D. H., Busby, R. C.), 15 Sep 2014, 1 ♂ [FLMNH-MGCL-195372], (FLMNH), (Busby, R. C., Ahrenholz, D. H.), 8 Jan 2015, 1 ♂ [FLMNH-MGCL-195464], 1 ♂ [FLMNH-MGCL-195465], (FLMNH); km 47.6 Santiago–Puerto Morona rd., [2°56'12"S,77°44'48"W], 245 m, (Busby, R. C.), 7 Jan 2015, 1 ♂ [FLMNH-MGCL-195445], (FLMNH); Río Waiwaim, Taisha, [2°22'36"S,77°30'W], 600 m, (Hall, J. P. W.), 1,4 Jun 1994, 1 ♂, (FLMNH); *Napo*: Aguano, [1°4'S,77°33'W], 350 m, (Simpson, A.), 1 ♂ [BMNH(E)#1420056], (NHMUK); Archidona, [0°55'S,77°48'W], 24 May 1900, 1 ♂, (ZSM); Cordillera Galeras, [0°49'24"S,77°34'6"W], 1187 m, (Radford, J.), 11 Aug 2010, 1 ♂ [GAN26], (FLMNH) (CULEPEX Expedition, 2010); km 14 Tena–Puyo rd., Apuya, [1°6'18"S,77°46'42"W], 600 m, (Busby, R. C.), 10 Sep 2014, 1 ♂ [FLMNH-MGCL-195370], (FLMNH); Río Anzu, [1°10'30"S,77°52'22"W], 700 m, (Velástegui, D.), 9 Jan 1969, 1 ♂ [MUSM-LEP-100441], (MUSM); Río Arajuno, [1°14'S,77°42'W], 900 m, (Brown, F. M.), 26 Apr 1941, 1 ♂ [FLMNH-MGCL-263325], (FLMNH); Río Misahuallí 10 min by boat above the junction with Río Napo, [1°0'2"S,77°40'23"W], 488 m, (Harris, L. N.), 14 Nov 1997, 1 ♂ [FLMNH-MGCL-257127], 1 ♂ [FLMNH-MGCL-264666], (FLMNH); Río Misahuallí, [1°1'36"S,77°40'W], 518 m, (Bowe, J. J.), 14 Sep 1998, 1 ♂ [FLMNH-MGCL-257123], (FLMNH), 16 Sep 1998, 1 ♂ [FLMNH-MGCL-257122], (FLMNH), 8 Sep 1998, 1 ♂ [FLMNH-MGCL-257124], (FLMNH), 9 Sep 1998, 1 ♂ [FLMNH-MGCL-257121], (FLMNH); Río Misahuallí, Misahuallí, [1°1'36"S,77°40'W], 350–400 m, (Nakamura, I.), 21 Jun 2006, (ICNA), 22 Jun 2006, (ICNA), 23 Jun 2006, (ICNA); Río Misahuallí, Misahuallí, [1°1'36"S,77°40'W], 400 m, (Smith, J. A.), 7–15 Sep 1997, 1 ♂, (BME), 8-14 Sep 1996, 1 ♂, (BME), (Taylor, T.), 26 Apr 1971, 1 ♂ [FLMNH-MGCL-263340], (FLMNH), 28 Apr 1971, 1 ♂ [FLMNH-MGCL-263348], (FLMNH); Río Misahuallí, Tena, [0°59'28"S,77°49'6"W], 500 m, (Jenkins, D. W. & J.), 23 Jan 1985, 1 ♂ [FLMNH-MGCL-257128], 1 ♂ [FLMNH-MGCL-257129], 1 ♂ [FLMNH-MGCL-263053], (FLMNH); Río Napo, 600 m, (Lafebre, R. de), 11 Sep 1968, 1 ♂ [FLMNH-MGCL-263354], (FLMNH); Río Napo, Misahuallí, [1°1'36"S,77°40'W], 518 m, (Bowe, J. J.), 16 Sep 1998, 1 ♂ [FLMNH-MGCL-263050], (FLMNH); Río Napo, Puerto Napo-Ahuano rd., Chichicorrumi, [1°4'11"S,77°37'45"W], 450 m, (Hall, J. P. W., Willmott, K. R.), 7 Sep 1993, 1 ♂ [FLMNH-MGCL-257126], (FLMNH); Tena-Baeza rd., Cotundo, [0°50'42"S,77°47'44"W], 750 m, (Lafebre, R. de), Oct 1975, 1 ♀ [FLMNH-MGCL-257131], (FLMNH); Tena-Baeza rd., Cotundo, [0°50'42"S,77°47'44"W], 800 m, (Nicolay, S. S.), 18 Dec 1976, 1 ♂ [FLMNH-MGCL-263358], (FLMNH); Tres Cruces, 1960 m, 28 Feb 1958, 1 ♂ [FLMNH-MGCL-263055], (FLMNH); *Orellana*: 48.5 km S Pompeya, Bogi 2 W stream, [0°42'44"S,76°28'29"W], 230 m, (Hall, J. P. W., Willmott, K. R., J. C. R., J. I. R), 28 Jul 2015, 3 ♂, (INABIO); Lago Agrio, [0°5'N,76°53'W], 300 m, 26 Jul 1978, 1 ♂ [FLMNH-MGCL-257130; J. J. Anderson collection], (FLMNH); lower Río Yasuní, 'Baradero' trail, [1°5'S,75°29'8"W], 170–220 m, (Willmott, K. R., J. I. R., J. C. R.), 18 Jul 2015, 1 ♂, 1 ♀, (INABIO); lower Río Yasuní, 'Pichincha' trail, [1°3'11"S,75°27'53"W], 170 m, (Willmott, K. R., J. I. R., J. C. R.), 22 Jul 2015, 1 ♂, (INABIO); Parque Nacional Yasuní, 10 km E Guardianía Pindo, [0°43'6"S,76°39'8"W], 330 m, (Hall, J. P. W., Willmott, K. R., J. C. R., J. I. R), 21,22 Jul 2016, (INABIO); Puerto Francisco de Orellana, [0°19'15"S,78°56'6"W], 1421 m, (Majerus, M.), 6 Mar 2007, 1 ♀ [BMNH(E)#1420052], (NHMUK); Reserva Biológica del Río Bigal, [0°31'41"S,77°25'7"W], 976 m, (Radford, J.), 21 Jul 2010, 1 ♀ [N15], (INABIO) (CULEPEX Expedition, 2010); Reserva Biológica del Río Bigal, [0°31'42"S,77°25'20"W], 917 m, (Radford, J.), 21 Jul 2010, 1 ♂ [N20], (FLMNH) (CULEPEX Expedition, 2010); Reserva Biológica del Río Bigal, main campsite, [0°31'30"S,77°25'4"W], 950 m, (Segebarth, C.), 1 Nov 2011, 1 ♂, (collection unknown) (Segebarth, C., pers. comm.), (Turner, J. D.), 30 Oct 2011, 1 ♂, (INABIO); Reserva Biológica del Río Bigal, main campsite, [0°31'30"S,77°25'4"W], 955 m, (Radford, J.), 21 Jul 2010, 1 ♂ [N13], (INABIO) (CULEPEX Expedition, 2010); Río Aguarico, Zancudococha, [0°34'23"S,75°26'13"W], 240 m, (Willmott, K. R., J. C. R., J. I. R., Aldaz, R.), 3, 5–8 Jul 2017, 1 ♂ [FLMNH-MGCL-288704], (FLMNH), (Willmott, K.R., J.C.R, J.I.R., Aldaz, R.), 3, 5–8 Jul 2017, (INABIO); Río Coca, [0°28'S,76°58'W], 300 m, (Lafebre, R. de), Jul 1971, 1 ♂ [FLMNH-MGCL-263341], 1 ♀ [FLMNH-MGCL-263378], (FLMNH), Jul 1971, 1 ♀ [FLMNH-MGCL-263337], (FLMNH), Jun 1971, 1 ♀ [FLMNH-MGCL-263372], (FLMNH); Río Napo, Sacha Lodge, [0°28'14"S,76°27'33''w], 240 m, (Gallice, G.), 21 Oct 2010, 1 ♂, (INABIO); Río Tiputini, Estación Científica Yasuní, parcela 50 Ha, [0°40'55"S,76°24'1"W], 250–270 m, (Willmott, K. R., J. I. R., J. C. R., Páez, E.), 7 Jul 2014, 1 ♀, (INABIO); Río Tiputini, Tiputini Biodiversity Station, [0°42'12"S,76°0'30"W], 300 m, (Melo, P.), May–Aug 2002, 1 ♀, (FLMNH); Río Tiputini, vía Auca, Estación Científica Yasuní, [0°40'27"S,76°23'49"W], 220-250 m, (Gallice, G.), 14 Jul 2009, 1 ♂ [FLMNH-MGCL-152425], (FLMNH), 4 Aug 2009, 1 ♂ [FLMNH-MGCL-152427], (FLMNH), (Harris, L. N.), 4 Oct 1998, 1 ♂ [FLMNH-MGCL-257135], (FLMNH), (Robinson Willmott, J. I., J. C.), 5 Jul 2014, 1 ♂, (INABIO); Río Tiputini, vía Auca, Estación Científica Yasuní, [0°40'27"S,76°23'49"W], 250 m, (Hall, J. P. W., Willmott, K. R., J. C. R., J. I. R), 26 Jul 2015, 1 ♂ [FLMNH-MGCL-209454], (FLMNH), 2 ♂, (INABIO); Yasuní, Estación Científica, [0°40'17"S,77°24'2"W], 229 m, (Majerus, M.), 1 Mar 2007, 1 ♂ [BMNH(E)#1420050], 1 ♂ [BMNH(E)#1420051], (NHMUK); Yasuní, Estación Científica, [0°40'17"S,77°24'2"W], 250 m, (Lamas, G.), 6 Dec 2004, 1 ♂ [MUSM-LEP-100523], (MUSM); Yasuní, Estación Científica, [0°40'17"S,77°24'2"W], 300 m, (Harris, L.), 8 Sept 2002, 1 ♂ [FLMNH-MGCL-1036227], (FLMNH); *Pastaza*: 10.5 km SW Palora, [1°45'20"S,78°1'49"W], 1000 m, (Hall, J. P. W., Willmott, K. R., J. C. R., J. I. R), 2 Aug 2015, 1 ♂, (INABIO); 25 km E of Puyo, 762 m, (Stevens, C. M.), 28 Jun 1980, 1 ♂ [FLMNH-MGCL-257133], 1 ♂ [FLMNH-MGCL-257134], (FLMNH); km 11 Mera-Río Anzu rd., [1°25'15"S,78°3'8"W], 1200 m, (Hall, J. P. W., Willmott, K. R., J. C. R., J. I. R), 31 Jul 2015, 1 ♂, (INABIO); nr. Mera, Río Puyo, [1°28'S,77°59'W], 1000 m, (McIntyre, W.), 24 Feb 1950, 1 ♂, (CMNH); Nushiño, [1°14'S,77°34'30"W], (Simpson, A.), 1 ♂ [BMNH(E)#1420055], (NHMUK); Pastaza, [1°34'36"S,77°45'W], 1 ♂ [BMNH(E)#1498855; 'forest on edge'], (NHMUK); Puyo, [1°28'S,77°59'W], 1000 m, 1 ♀, (CMNH), (McIntyre, W.), 17 Mar 1950, 1 ♀, (CMNH), 23 Mar 1950, 1 ♀, (CMNH), 24 Feb 1950, 1 ♀, (CMNH), 24 Mar 1950, 1 ♂, (CMNH), 29 Apr 1950, 1 ♀, (CMNH), 30 May 1950, 1 ♀, (CMNH), Apr 1950, 1 ♀, (CMNH), Feb 1950, 1 ♀, (CMNH), Jul 1950, 2 ♂, 1 ♀, (CMNH); Puyo, [1°28'S,77°59'W], 1067 m, (Nicolay, S. S.), 6 Dec 1972, 1 ♂ [FLMNH-MGCL-263324], (FLMNH), 7 Dec 1972, 1 ♂ [FLMNH-MGCL-263320], (FLMNH), 8 Dec 1972, 1 ♂ [FLMNH-MGCL-263336], (FLMNH), 9 Dec 1972, 1 ♂ [FLMNH-MGCL-263330], 1 ♀ [FLMNH-MGCL-263380], (FLMNH); Puyo-Macas rd., Pitirishca, [1°48'18"S,77°49'15"W], 800 m, (Godefroi, R., Busby, R. C.), Sep 2001, 1 ♂ [FLMNH-MGCL-257132], (FLMNH); Río Bobonaza, Canelos, [1°35'S,77°45'W], 640 m, (Palmer, M. G.), 1 ♂ [BMNH(E)#1420057], (NHMUK); Río Bobonaza, Sarayacu, [1°44'S,77°29'W], 700 m, (Lafebre, R. de), 15 Sep 1968, 1 ♂ [FLMNH-MGCL-263342], (FLMNH); Río Capahuari, Kapawi Lodge, [2°32'30"S,76°51'32"W], 250 m, (Willmott, K. R., Hall, J. P. W.), 21,22,27 Jul 2009, 1 ♂ [FLMNH-MGCL-149529], (FLMNH); Río Pastaza, (Lafebre, R. de), Dec 1968, 1 ♀ [FLMNH-MGCL-263373], (FLMNH); Río Pastaza, Kapawi village, [2°32'16"S,76°50'10"W], 260 m, (Willmott, K. R., Hall, J. P. W.), 23 Jul 2009, 1 ♂ [FLMNH-MGCL-149525], 1 ♀ [FLMNH-MGCL-149526], (FLMNH); Río Pindo Grande, Shell, [1°29'40"S,78°3'40"W], 1050 m, (Willmott, K. R., Hall, J. P. W.), 7,8 Feb 1995, (FLMNH); Shell-Mera Rd., (Taylor, T.), 28 Feb 1971, 1 ♂ [FLMNH-MGCL-263321], 1 ♂ [FLMNH-MGCL-263347], (FLMNH); Yutsuntsa, [2°21'4"S,76°27'14"W], 250 m, (Nakahara, S.), 9 Jul 2014, 1 ♂/♀, (FLMNH); *Pichincha*: 12 km E Santo Domingo de los Colorados, Hotel Tinalandia, [0°18'S,79°4'W], 650–750 m, (Nakamura, I.), 27 Jun 2006, (ICNA), 28 Jun 2006, (ICNA), 29 Jun 2006, (ICNA); *Sucumbíos*: Cerro Lumbaqui Norte, [0°1'42"N,77°19'W], 800–950 m, (Hall, J. P. W., Willmott, K. R., J. C. R., J. I. R), 15-19 Jul 2016, 1 ♂ [FLMNH-MGCL-209995], (FLMNH), (INABIO); Cuyabeno, Reserva de Producción Faunística, [0°0'4"S,76°10'50"W], 230 m, (Kareofelas, G., Witham, C. W.), 20 Nov-12 Dec 1993, 4 ♂, 2 ♀, (BME); km 10.5 Lumbaqui-Baeza rd., [0°0'15"S,77°25'W], 850 m, (Hall, J. P. W.), 17 Aug 2002, 1 ♂, (FLMNH); Río Napo, Limoncocha, [0°24'S,76°37'W], 300 m, 6 Apr 1977, 1 ♂ [BMNH(E)#1498854], (NHMUK), (Nicolay, S. S.), 10 Feb 1971, 1 ♂ [FLMNH-MGCL-263332], (FLMNH), 9 Feb 1971, 1 ♂ [FLMNH-MGCL-263331], (FLMNH); *Tungurahua*: Santa Inés, [1°24'44"S,78°13'43"W], 1250 m, (Simpson, A.), 1 ♀ [BMNH(E)#1420067], (NHMUK); *Zamora-Chinchipe*: 7 km SW Guayzimi, San José, [4°4'29"S,78°43'36"W], 1400 m, (Willmott, K. R., Hall, J. P. W.), 2 Aug 2009, 1 ♂ [FLMNH-MGCL-149522], (FLMNH); above Río Sabanilla, Soñaderos, [4°3'6"S,79°1'4"W], 1450 m, (Willmott, K. R.), 13 Nov 2010, 1 ♂, (INABIO); Cabañas Ecológicas Copalinga, Río Bombuscaro, [4°5'26"S,78°57'31"W], 1000 m, (Whelan, C.), 6 Dec 2008, 1 ♂ [FLMNH-MGCL-115951], (FLMNH), 8 Dec 2008, 1 ♂ [FLMNH-MGCL-115801], (FLMNH); E of Zumba, km 2.6 El Pite-Río Mayo rd., [4°51'58"S,79°5'40"W], 1000 m, (Willmott, K. R., J. I. R., J. C. R.), 26 Jun 2014, 1 ♀, (INABIO); E. of Chito, Tres Aguas, [4°53'31"S,78°59'34"W], 1850 m, (Willmott, K. R., J. I. R., J. C. R.), 5,6 Jun 2013, 1 ♂ [FLMNH-MGCL-157409], (FLMNH); km 11.5 Los Encuentros-Zarza, La Libertad, [3°47'54"S,78°36'26"W], (Nakahara, S.), 28 Jun 2014, 1 ♀, (FLMNH); km 11.5 Los Encuentros-Zarza, La Libertad, [3°47'54"S,78°36'26"W], 1250 m, (Willmott, K. R., Hall, J. P. W.), 6,8 Aug 2009, 1 ♂ [FLMNH-MGCL-149521], (FLMNH); km 18 Zumba-Los Sungas rd., Quebrada Huanchunangui, [4°55'11"S,79°9'54"W], 1100 m, (Willmott, K. R.), 11 Oct 2010, 1 ♂, (INABIO), (Willmott, K. R., J. I. R., J. C. R.), 2,3 Jun 2013, 1 ♂, (INABIO); km 2.5 Zumba-San Andrés rd., [4°51'18"S,79°3'47"W], 1370 m, (Willmott, K. R.), 12 Oct 2010, 1 ♂, (INABIO); km 22 Zumba-Loja rd., [4°46'10"S,79°7'6"W], 1180 m, (Padrón, S., Aldaz, R.), 13 May 2008, 1 ♂ [FLMNH-MGCL-118493], (FLMNH); km 29 Zumba-Los Sungas rd., [4°57'4"S,79°12'40"W], 1400 m, (Willmott, K. R., J. I. R., J. C. R.), 18 Jun 2014, 1 ♀ [FLMNH-MGCL-195105], (FLMNH); km 5 Zumba–Chito rd., [4°53'18"S,79°7'26"W], 950 m, (Willmott, K. R., J. I. R., J. C. R.), 1 Jun 2013, 1 ♂ [FLMNH-MGCL-157408], (FLMNH), 1 ♂, (INABIO); lower Río Numpatakaime, c. 3 km S Shaime, [4°21'S,78°39'28"W], 900 m, (Willmott, K. R., Hall, J. P. W.), 31 Jul 2009, 1 ♂ [FLMNH-MGCL-149523], 1 ♂ [FLMNH-MGCL-149524], (FLMNH); nr. Zamora, Río Bombuscaro, [4°6'48"S,78°57'54"W], (Nakahara, S.), 23 Jun 2014, 1 ♂/♀, (FLMNH); Progreso, c. km 10 Zumba-Valladolid rd., [4°50'2"S,79°6'6"W], 1400 m, (Padrón, S., Aldaz, R.), 15 May 2008, 1 ♂ [FLMNH-MGCL-118490], 1 ♂ [FLMNH-MGCL-118491], (FLMNH); rd. Zumbi-Yankuam, Quebrada Numbame, [4°9'5"S,78°38'40"W], 900 m, (Willmott, K. R., Hall, J. P. W.), 29 Jul 2009, 1 ♂ [FLMNH-MGCL-149527], 1 ♂ [FLMNH-MGCL-149528], (FLMNH); Río Mariposa, Nangaritza, 900 m, (Parker, T. A.), 28 Jul 1993, 1 ♂ [MUSM-LEP-100442], (MUSM); Río Nangaritza, 4 km S Zurmi, Reserva Maycú, [4°12'45"S,78°38'36"W], 850 m, (Willmott, K. R., J. I. R., J. C. R.), 28 Jun 2014, 1 ♀ [FLMNH-MGCL-195110], (FLMNH), 2 ♂, 2 ♀, (INABIO); Río Zamora, Zamora, [4°4'6"S,78°57'W], 1050 m, 1885, 1 ♂ [BMNH(E)#1498840], (NHMUK); Río Zamora, Zamora, [4°4'6"S,78°57'W], 920-1220 m, (Baron, O. T.), 1 ♂ [BMNH(E)#1498829], 1 ♂ [BMNH(E)#1498830], 1 ♂ [BMNH(E)#1498831], 1 ♂ [BMNH(E)#1498833], 1 ♂ [BMNH(E)#1498834], 1 ♂ [BMNH(E)#1498835], 1 ♂ [BMNH(E)#1498836], 1 ♂ [BMNH(E)#1498837], 1 ♂ [BMNH(E)#1498838], 1 ♀ [BMNH(E)#1498832], 1 ♀ [BMNH(E)#1498841], 1 ♀ [BMNH(E)#1498842], 1 ♀ [BMNH(E)#1498843], (NHMUK); Yacuambí, [3°38'10"S,78°55'34"W], 1250 m, (Aldaz, R.), 26, 29 Sep 2007, 1 ♂ [FLMNH-MGCL-113705], (FLMNH); Yacuambí-Tutupali rd., Cascada Tres Chorros, [3°32'43"S,78°57'54"W], 1525 m, (Willmott, K. R., J. I. R., J. C. R.), 18 Jun 2013, 1 ♂ [FLMNH-MGCL-157410], (FLMNH), (INABIO); 'Zamora Podocarpus' [=Río Bombuscaro], [4°6'48"S,78°57'54"W], 1000 m, (Kareofelas, G., Witham, C. W.), 7 Feb 1998, 2 ♂, (BME); Zamora, ridge to west, [4°4'30"S,78°58'7"W], 1450 m, (Godefroi, R., Busby, R. C.), 18 Sep 2000, 1 ♂ [FLMNH-MGCL-257136], (FLMNH); Zumba-Loja rd., Palanda, [4°40'S,79°8'W], 1150 m, 1986, 1 ♂ [BMNH(E)#1420061], (NHMUK), 7 Aug 1886, 1 ♂ [BMNH(E)#1498858], (NHMUK); *Not located*: ‘Ecuador’, 1 ♂, (MNHU). **French Guiana**: *Cayenne*: 13 km SE Sinnamary on Rte. N1, CIRAD Forest reserve, (Edwards, G. B.), 5–7 Apr 1999, 1 ♀ [FLMNH-MGCL-262965], (FLMNH); Cayenne, [4°56'N,52°20'W], 1 ♂ [BMNH(E)#1420099], 1 ♂ [BMNH(E)#1420100], 1 ♂ [BMNH(E)#1420101], 1 ♂ [BMNH(E)#1420196], 1 ♂ [BMNH(E)#1420197], 1 ♂ [BMNH(E)#1420201], 1 ♀ [BMNH(E)#1420195], (NHMUK), 1 ♂, (MNHU); Kaw Mountains, Relais de Patawa Lodge, (Emmel, T. C., Sourakov, A.), 3–5 Mar 1995, 1 ♂ [FLMNH-MGCL-262962], (FLMNH); SW of Regina on Approuage River, Saut Anthanase Camp, [4°10'33"N,52°21'24"W], (Emmel, T. C., Sourakov, A.), 6-9 Mar 1995, 1 ♀ [FLMNH-MGCL-262963], (FLMNH), (Smith, J. A.), 14–17 Oct 1999, 1 ♀, (BME); vic. Saül airport, [4°33'33"N,52°12'25"W], 228 m, (Smith, N. J., Gilbert, A.), 8-14 Aug 2012, 1 ♂, (BME); vicinity of Cayenne, Airport/mountain reservoir, (Harris, L. N.), 4 May 1998, 1 ♂ [FLMNH-MGCL-262961], (FLMNH); *St-Laurent du Maroni*: Maroni River, (DZUP), 1 ♂ [FLMNH-MGCL-263114], 1 ♂ [FLMNH-MGCL-263115], 1 ♂ [FLMNH-MGCL-263117], 1 ♂ [FLMNH-MGCL-263118], 1 ♂ [FLMNH-MGCL-263119], 1 ♂ [FLMNH-MGCL-263120], 1 ♂ [FLMNH-MGCL-263121], 1 ♂ [FLMNH-MGCL-263122], 1 ♀ [FLMNH-MGCL-263132], (FLMNH); Maroni river, Maripasoula, [3°41'N,54°2'W], (Kassarov, L.), 20 Jan 1991, 1 ♀ [FLMNH-MGCL-262964], (FLMNH), Jan 1992, 1 ♀ [FLMNH-MGCL-1036237], (FLMNH); Saint-Georges-de-l'Oyapock, [3°53'33"N,51°48'27"W], (Brévignon, C.), 8 Feb 1985, 1 ♂ [MUSM-LEP-100519], (MUSM); Saül, [3°51'30"N,53°18'14"W], 200-450 m, 1991, (LBCB), (Nakahara, S.), 5 Aug 2014, 1 ♂ [FLMNH-MGCL-209434; 'hill'], (FLMNH); St. Laurent du Maroni, [5°30'N,54°2'W], 1 ♂ [BMNH(E)#1420102], 1 ♂ [BMNH(E)#1420103], 1 ♂ [BMNH(E)#1420104], 1 ♂ [BMNH(E)#1420105], (NHMUK); *Not located*: ‘French Guiana’, 1 ♂ [BMNH(E)#1420106], 1 ♂ [BMNH(E)#1420107], 1 ♂ [BMNH(E)#1420108], 1 ♂ [BMNH(E)#1498905], 1 ♀ [BMNH(E)#1420109], 1 ♀ [BMNH(E)#1420110], 1 ♀ [BMNH(E)#1420111], 1 ♀ [BMNH(E)#1420112], (NHMUK). **Guyana**: *Cuyuni-Mazaruni*: Bartica, [6°24'N,58°37'W], 1 ♂ [BMNH(E)#1498870], (NHMUK), (Parish, H. S.), 1 ♂ [BMNH(E)#1420082], (NHMUK), (Parish, H. S., Hoffmanns, W.), 1 ♀ [BMNH(E)#1420089], 1 ♀ [BMNH(E)#1420090], (NHMUK); Cariamang River, [5°42'N,59°38'W], (Whitely, H.), 1 ♂ [BMNH(E)#1420085], (NHMUK); Kamarang, [5°53'N,60°40'W], (Jenkins, D. W.), 10–14 Oct 1977, 1 ♂ [FLMNH-MGCL-257170], 1 ♂ [FLMNH-MGCL-264684], (FLMNH); 'Mazaruni Potaro', (Steiner, W. E.), 24 Dec 1983, 1 ♂ [FLMNH-MGCL-1036219], 1 ♂ [FLMNH-MGCL-1036221], (FLMNH); Paruima, [5°48'N,61°1'W], 610 m, (Pinhas, A. S.), 1938, 1 ♀ [FLMNH-MGCL-263130], (FLMNH); Roraima, [5°12'N,60°44'W], 1 ♂ [BMNH(E)#1420084], (NHMUK); *East Berbice-Corentyne*: New River Triangle, Camp Jaguar, 152 m, (Steinhauser, S. R.), 17 Nov 1980, 1 ♀ [FLMNH-MGCL-263371], (FLMNH), 18 Nov 1980, 1 ♂ [FLMNH-MGCL-263112], (FLMNH); *Upper Demerara-Berbice*: Essequibo River, Shanklands Resort, 36 mi SW of Georgetown, [6°28'44"N,58°34'54"W], 20 m, (Douglas, M. G.), 20–28 Sep 2006, 1 ♂ [FLMNH-MGCL-257159], 1 ♂ [FLMNH-MGCL-257160], 1 ♂ [FLMNH-MGCL-257161], 1 ♂ [FLMNH-MGCL-257162], 1 ♂ [FLMNH-MGCL-257163], 1 ♂ [FLMNH-MGCL-257164], 1 ♂ [FLMNH-MGCL-257165], 1 ♀ [FLMNH-MGCL-257166], 1 ♀ [FLMNH-MGCL-257167], 1 ♀ [FLMNH-MGCL-257168], (FLMNH); Omai, [5°26'N,58°45'W], (Schaus, W. M.), 1 ♂ [BMNH(E)#1420086], (NHMUK); *Not located*: ‘Guyana’, 1 ♂ [BMNH(E)#1420088], 1 ♂ [BMNH(E)#1498871], 1 ♂ [BMNH(E)#1498872], (NHMUK), (Bartlett, A. W.), 1 ♂ [BMNH(E)#1498904], 1 ♀ [BMNH(E)#1498965], (NHMUK), (Parish, H. S.), 1 ♂ [BMNH(E)#1498903], (NHMUK); Demerara river, 1 ♀ [BMNH(E)#1498873], (NHMUK); Demerara, [6°47'N,58°10'W], (Holmes, W.), 1 ♂ [BMNH(E)#1420083], (NHMUK); Parish, [4°46'N,58°15'W], 1 ♂ [BMNH(E)#1420087], (NHMUK); Rio Essequibo, Makouria, Feb 1999, 1 ♂ [FLMNH-MGCL-264645], (FLMNH), Feb 1999, 1 ♂ [FLMNH-MGCL-264648], (FLMNH); Suruwabaru CK, Mt. Wokamung, 686 m, (Fratello, S.), Nov, 1 ♂ [FLMNH-MGCL-257169], (FLMNH). **Peru**: *Amazonas*: 0–5 km E La Peca, [5°37'S,78°26'W], 1100–1400 m, (Lamas, G.), 23 Sep 1999, 1 ♂ [MUSM-LEP-100527], (MUSM); 4 km W Abra Wawajin, [5°18'S,78°24'W], 750 m, (Lamas, G.), 24 Sep 1999, 1 ♀ [MUSM-LEP-100536], (MUSM); Alfonso Ugarte, [3°55'S,78°26'W], 1000–1200 m, (Lamas, G.), 15 Jul 1994, 1 ♂ [MUSM-LEP-100452], 1 ♂ [MUSM-LEP-100453], (MUSM), 16 Jul 1994, 1 ♂ [MUSM-LEP-100450], 1 ♂ [MUSM-LEP-100451], (MUSM); Cordillera del Cóndor, P.V. 12 de Enero (P.V. 32), [3°39'30"S,78°18'552"W], 700 m, (Grados, J., Asenjo, A.), 23 Nov 2003, 1 ♂ [MUSM-LEP-100456], (MUSM); Cordillera del Cóndor, Qda. Chinganasa (Qda. Ponce) c.a. P.V., [3°46'47"S,78°20'W], 680 m, (Grados, J., Asenjo, A.), 10 Nov 2003, 1 ♂ [MUSM-LEP-100459], (MUSM); Cordillera del Cóndor, Qda. Chinganasa (Qda. Ponce) c.a. P.V., [3°46'47"S,78°20'W], 700–850 m, (Grados, J., Asenjo, A.), 13 Nov 2003, 1 ♂ [MUSM-LEP-100457], 1 ♂ [MUSM-LEP-100461], (MUSM); Cordillera del Cóndor, Qda. Chinganasa (Qda. Ponce) c.a. P.V., [3°46'47"S,78°20'W], 850–1160 m, (Grados, J., Asenjo, A.), 15 Nov 2003, 1 ♂ [MUSM-LEP-100454], 1 ♂ [MUSM-LEP-100455], 1 ♂ [MUSM-LEP-100458], 1 ♂ [MUSM-LEP-100460], (MUSM); Cordillera del Cóndor, Quebrada Kegkem, [3°38'S,78°18'W], 700 m, (Grados, J., Asenjo, A.), 20 Nov 2003, 1 ♀ [MUSM-LEP-100534], (MUSM); Falso Paquisha, [3°58'S,78°25'W], 800 m, (Lamas, G.), 22 Oct 1987, 1 ♂ [MUSM-LEP-100445; in copula], 1 ♂ [MUSM-LEP-100446; in copula], 1 ♂ [MUSM-LEP-100448; in copula], (MUSM), 23 Oct 1987, 1 ♂ [MUSM-LEP-100447; in copula], (MUSM), 25 Oct 1987, 1 ♀ [MUSM-LEP-100533; in copula], (MUSM), 26 Oct 1987, 1 ♂ [MUSM-LEP-100449; in copula], (MUSM), 30 Oct 1987, 1 ♂ [MUSM-LEP-100444; in copula], 1 ♀ [MUSM-LEP-100532; in copula], (MUSM); Mendoza, Quebrada Yanahuayco, [6°24'S,77°26'W], 1600-1800 m, (Calderón, B.), Aug 1998, 1 ♀ [MUSM-LEP-100538], (MUSM); near La Iguana, [5°41'S,78°24'W], 850 m, (Lamas, G.), 26 Jun 1995, 1 ♀ [MUSM-LEP-100535], (MUSM); Nueva Esperanza, 3 km N, 1700 m, (Calderón, B.), 11 Mar 1986, 1 ♂ [MUSM-LEP-100462], (MUSM); Pongo de Retema, Rentema Falls, [5°29'S,78°33'W], 305 m, (Pratt, A. & E.), 1 ♂ [BMNH(E)#1420181], (NHMUK); Quebrada Gebil, [6°28'S,77°24'W], 1300 m, (Lamas, G.), 20 Sep 1999, 1 ♀ [MUSM-LEP-100537], (MUSM); *Cajamarca*: 5 km W Jaén, [5°42'S,78°50'W], 800 m, (Lamas, G.), 10–11 Nov 1975, 1 ♀ [MUSM-LEP-100531], (MUSM); Charape, [5°25'S,78°59'W], 1220 m, (Pratt, A. & E.), Sep–Oct 1912, 1 ♀ [BMNH(E)#1420184], (NHMUK); W de Tamborapa, 600 m, (Lamas, G.), 17 Mar 1985, 1 ♂ [MUSM-LEP-100443], (MUSM); *Cuzco*: Cosñipata, Quebrada Quitacalzón, [13°1'S,71°30'W], 1100 m, (Harris, B.), 7 Feb 2011, 1 ♂ [MUSM-LEP-100520], (MUSM), (Lamas, G.), 10 May 2012, 1 ♀ [MUSM-LEP-100562], (MUSM), 26 Apr 2015, 1 ♀ [MUSM-LEP-100902], (MUSM), 27 Jan 2013, 1 ♂ [MUSM-LEP-100528], (MUSM); Pilcopata, [12°55'S,71°24'W], 550 m, (Lamas, G.), 5 Feb 2010, 1 ♂ [MUSM-LEP-100479], (MUSM); Quincemil, [13°14'S,70°46'W], 600–800 m, (Steiner, W. E.), 30 Jan 1979, 1 ♀ [FLMNH-MGCL-262960], (FLMNH); Quincemil, Quebrada Yanaorcco, [13°16'S,70°47'W], 720 m, (Rodríguez, M.), 16 Oct 2009, 1 ♂ [MUSM-LEP-100521], 1 ♂ [MUSM-LEP-100525], (MUSM), 9 Dec 2009, 1 ♀ [MUSM-LEP-100560], (MUSM); Río Urubamba, Kitaparay, [12°11'8"S,72°49'14"W], 459 m, (Cerdeña, J., Farfán, J., Gutiérrez, R.), 4 Sep 2007, 1 ♂ [MUSM-LEP-100477], (MUSM); Río Urubamba, Timpia, [12°5'31"S,72°49'12"W], 408 m, (Cerdeña, J., Farfán, J., Gutiérrez, R.), 25 Aug 2007, 1 ♂ [MUSM-LEP-100478], (MUSM); *Huánuco*: ‘Huánaco’ [= Huánuco], 1900–1980 m, (Kassarov, L.), Apr 1992, 1 ♂ [FLMNH-MGCL-1036224], (FLMNH); 13 km S Tingo María, Tambillo Chico Canyon, [9°8'S,75°57'W], (Weisner, K. J.), 24 Jun 1982, 1 ♂ [FLMNH-MGCL-262959], (FLMNH); 15 km N Tingo María on Río Huallaga, (Rojas, M.), 15-22 Aug 1981, 1 ♂ [FLMNH-MGCL-262956], 1 ♂ [FLMNH-MGCL-262957], (FLMNH); 8 km S Tingo María, Cueva de Las Pavas, [9°24'S,75°58'W], 900 m, (DZUP), (Harris, L. & H.), 21 Jun 1985, 1 ♂ [FLMNH-MGCL-262950], (FLMNH); Cordillera del Sira, [9°25'S,74°45'W], 750 m, (Exp. Universidad Viena), Sep 1987–Aug 1988, 1 ♂ [MUSM-LEP-100465], 1 ♂ [MUSM-LEP-100466], 1 ♂ [MUSM-LEP-100467], 1 ♂ [MUSM-LEP-100468], (MUSM); Lower Ucayali, Río Pachitea, [8°46'S,74°32'W], 150 m, (Tessmann), 3 ♂, (MNHU); Tingo María, [9°18'S,76°0'W], 800 m, May 1987, 1 ♀ [FLMNH-MGCL-262955], (FLMNH), (Kassarov, L.), Sept 1992, 1 ♀ [FLMNH-MGCL-1036234], (FLMNH), (Rosier, P.), Jun 1979, 1 ♂ [FLMNH-MGCL-262958], (FLMNH); vicinity of Tingo María, (Rosier, P.), May 1979, 1 ♂ [FLMNH-MGCL-263365], (FLMNH); *Junín*: Cerro Conchapen, [10°49'S,75°11'W], 1000 m, (Hocking, P.), 5 May 1969, 1 ♂ [MUSM-LEP-100474], (MUSM); Cerro Conchapen, [10°49'S,75°11'W], 1200 m, (Hocking, P.), 5 May 1969, 1 ♂ [MUSM-LEP-100475], (MUSM); Chanchamayo, [11°4'S,75°19'W], 1000-1400 m, Oct 1960, 1 ♀, (ZSM), (König, F.), May 1959, 1 ♀, (ZSM), (Schuncke, O.), 1892, 1 ♂ [BMNH(E)#1420172], 1 ♂ [BMNH(E)#1420173], 1 ♂ [BMNH(E)#1420174], (NHMUK), 1912, 1 ♂ [BMNH(E)#1420168], 1 ♂ [BMNH(E)#1420169], 1 ♂ [BMNH(E)#1420170], 1 ♂ [BMNH(E)#1420171], 1 ♂ [BMNH(E)#1498932], 1 ♂ [BMNH(E)#1498933], 1 ♂ [BMNH(E)#1498934], 1 ♂ [BMNH(E)#1498935], (NHMUK), (Tessmann), 1 ♀, (MNHU); Chanchamayo, [11°4'S,75°19'W], 750 m, Mar 1962, 1 ♂, (ZSM); Chanchamayo, Río Ulcumayo valley NW San Ramón, trail up river, [10°59'1"S,75°26'28"W], 1250–1370 m, (Nakamura, I.), 12 Dec 2008, (ICNA), 4 Dec 2008, (ICNA), 5 Dec 2008, (ICNA), Dec 2008, (ICNA); Chanchamayo, Río Ulcumayo, Pampa Hermosa, [11°25'S,74°46'W], 1300-1400 m, (Medina, M.), 10 Jun 2008, 1 ♂ [MUSM-LEP-100476], (MUSM); La Merced, [11°3'S,75°19'W], 762 m, (Watkins, Tomlinson), May, Jun 1903, 1 ♀ [BMNH(E)#1420185], (NHMUK); Pampa Hermosa Lodge, [10°59'15"S,75°25'25"W], 1560 m, (Smith, J. A.), 2–12 Dec 2008, 2 ♂, (BME); Provincia de Satipo, Alto Cheni, 800 m, 1–15 May 1975, 1 ♂ [FLMNH-MGCL-262947], (FLMNH); Río Perené, [11°9'S,74°18'W], 610–1220 m, 1 ♂ [BMNH(E)#1498931], (NHMUK), (Martin, P.), 1 ♂, 2 ♀, (CMNH); Río Perené, [11°9'S,74°18'W], 700 m, (Schunke, J. M.), 20 Sep 1979, 1 ♀ [FLMNH-MGCL-262952], (FLMNH); San Ramón, [11°8'S,75°21'W], 915 m, (Watkins, Tomlinson), Aug 1903, 1 ♂ [BMNH(E)#1420176], (NHMUK); Satipo, [11°15'S,74°38'W], 800 m, (Schunke, J. M.), 5 May 1981, 1 ♂ [FLMNH-MGCL-262946], (FLMNH), 6 May 1981, 1 ♀ [FLMNH-MGCL-262951], (FLMNH); upper Río Toro, [11°3'S,75°19'W], 3000 m, (Simmons), Aug, Sep 1901, 1 ♀ [BMNH(E)#1498928], (NHMUK); *Loreto*: ‘Ampyam’ [= Río Ampiyacu], [3°19'S,71°51'W], 120 m, Jun-Aug 1918, 1 ♂ [BMNH(E)#1498936], (NHMUK); ‘Cachiacu’ [= Río Cachiyacu], [5°50'S,76°33'W], 200 m, (Stuart), 1 ♂ [BMNH(E)#1498920], 1 ♂ [BMNH(E)#1498921], (NHMUK); 80 km E Iquitos, Yanamono, [3°27'S,72°51'W], 120 m, (Lamas, G., Mallet, J.), 27 Jul 1984, 1 ♀ [MUSM-LEP-100542], (MUSM); 9 km S La Vista, [4°40'52"S,77°26'7"W], 250 m, (Lamas, G.), 11 Feb 1978, 1 ♂ [MUSM-LEP-100481], (MUSM); Castaña, [0°48'S,75°14'W], 150 m, (Lamas, G.), 19 Oct 1993, 1 ♂ [MUSM-LEP-100488], (MUSM), 24 Oct 1993, 1 ♂ [MUSM-LEP-100485], 1 ♀ [MUSM-LEP-100546], (MUSM), 26 Oct 1993, 1 ♂ [MUSM-LEP-100484], (MUSM), (Robbins, R. K.), 20 Oct 1993, 1 ♂ [MUSM-LEP-100486], 1 ♂ [MUSM-LEP-100487], 1 ♂ [MUSM-LEP-100489], (MUSM), 29 Oct 1993, 1 ♀ [MUSM-LEP-100545], (MUSM); Explornapo-ACEER, [3°14'S,72°55'W], 140 m, (Caldas, A.), 5 Sep 1995, 1 ♀ [MUSM-LEP-100547], (MUSM), 6 Sep 1995, 1 ♂ [MUSM-LEP-100495], (MUSM), 7 Sep 1995, 1 ♂ [MUSM-LEP-100492], 1 ♂ [MUSM-LEP-100497], (MUSM), (Grados, J.), 11 Sep 1995, 1 ♂ [MUSM-LEP-100491], (MUSM), 5 Sep 1995, 1 ♂ [MUSM-LEP-100493], (MUSM), (Harvey, D. J.), 10 Sep 1995, 1 ♀ [MUSM-LEP-100549], (MUSM), 22 Sep 1995, 1 ♂ [MUSM-LEP-100496], (MUSM), 5 Sep 1995, 1 ♀ [MUSM-LEP-100550], (MUSM), (Lamas, G.), 21 Sep 1995, 1 ♂ [MUSM-LEP-100490], (MUSM), 5 Sep 1995, 1 ♂ [MUSM-LEP-100494], (MUSM), 6 Sep 1995, 1 ♂ [MUSM-LEP-100498], (MUSM), (Robbins, R. K.), 16 Sep 1995, 1 ♂ [MUSM-LEP-100499], (MUSM), 9 Sep 1995, 1 ♀ [MUSM-LEP-100548], (MUSM); Iquitos, [3°45'S,73°15'W], 100 m, Feb 1989, 1 ♂ [FLMNH-MGCL-262949], (FLMNH), Jul 1988, 1 ♀ [FLMNH-MGCL-262953], (FLMNH), (Callegari, M.), Feb 2004, 1 ♀ [MUSM-LEP-100565], (MUSM); Iquitos, San Roque, [3°48'S,73°20'W], 1 ♂ [FLMNH-MGCL-263361], (FLMNH); Jenaro Herrera, [4°55'S,73°40'W], 125 m, (Lamas, G.), 12 Aug 2013, 1 ♂ [MUSM-LEP-100530], (MUSM), 16 Aug 2013, 1 ♂ [MUSM-LEP-100529], 1 ♀ [MUSM-LEP-100563], (MUSM), 6 Aug 2013, 1 ♀ [MUSM-LEP-100564], (MUSM); Lower Río Ucayali, Río Pacaya, Aug-Sep 1912, 1 ♂ [BMNH(E)#1420177], 1 ♂ [BMNH(E)#1420178], (NHMUK); Nauta, [4°30'S,73°35'W], 120 m, (Lequerica, H.), 25 Sep 1990, 1 ♂ [MUSM-LEP-100482], (MUSM); near Yanayacu River mouth, Ayzana Village, (Eiler, D. L.), 27 Jul 1989, 1 ♀ [FLMNH-MGCL-262954], (FLMNH); nr. Iquitos, Explornapo Camp, [3°15'S,72°55'W], 120 m, (Brown, H. B.), 9 Feb 1982, 1 ♂ [FLMNH-MGCL-1036215], (FLMNH); Pebas, [3°19'S,71°51'W], 120 m, 1 ♂ [BMNH(E)#1420126], 1 ♂ [BMNH(E)#1420180], (NHMUK), Jun 1992, 1 ♂ [FLMNH-MGCL-262948], (FLMNH), (Hahnel), 1 ♂, (MNHU), (Mathan, M. de), 1880, 1 ♂ [BMNH(E)#1420125], (NHMUK), Dec 1906, 1 ♂ [BMNH(E)#1498917], 1 ♀ [BMNH(E)#1498918], (NHMUK), Jan 1907, 1 ♂ [BMNH(E)#1498919], (NHMUK), Nov 1906, 1 ♂ [BMNH(E)#1498912], 1 ♂ [BMNH(E)#1498913], 1 ♂ [BMNH(E)#1498914], 1 ♂ [BMNH(E)#1498915], 1 ♀ [BMNH(E)#1498916], (NHMUK); Río Aguas Negras, [0°31'24"S,75°15'24"W], 150 m, (Lamas, G.), 6 Mar 1994, 1 ♀ [MUSM-LEP-100544], (MUSM), (Robbins, R. K.), 2 Mar 1994, 1 ♂ [MUSM-LEP-100483], (MUSM); Río Maquía, Arica, [6°26'S,74°46'W], 110 m, (Peña, C.), 17 Jul 2009, 1 ♀ [MUSM-LEP-100555], (MUSM); Río Nanay, Mishana, Estación Biológica Callicebus, [3°54'S,73°29'W], 150 m, (Lamas, G.), 16 Jan 1980, 1 ♂ [MUSM-LEP-100480], (MUSM); Río Tigre, San Jacinto, [2°19'S,75°52'W], 200 m, (Debinski, D.), 18 Apr 1993, 1 ♀ [MUSM-LEP-100543], (MUSM); Yurimaguas, [5°54'S,76°6'W], 120 m, (Hahnel), 1 ♀, (MNHU), (Mathan, M. de), Jun–Aug 1885, 1 ♂ [BMNH(E)#1420175], (NHMUK); Zona Reservada Allpahuayo-Mishana, [3°57'30"S,73°25'30"W], 170 m, (Campos, L.), 27 Nov 2001, 1 ♂ [MUSM-LEP-100504; varillal seco], (MUSM), 28 Feb 2002, 1 ♀ [MUSM-LEP-100551; Yarinal], (MUSM), (Ramírez, J. J.), 18 Feb 2002, 1 ♀ [MUSM-LEP-100552; Yarinal], (MUSM), 19 Nov 2001, 1 ♂ [MUSM-LEP-100502; varillal seco], (MUSM), 20 Nov 2001, 1 ♂ [MUSM-LEP-100501; varillal seco], (MUSM), 21 Feb 2002, 1 ♂ [MUSM-LEP-100503; Yarinal], (MUSM), 21 Nov 2001, 1 ♀ [MUSM-LEP-100553; varillal seco], (MUSM), 23 Feb 2002, 1 ♀ [MUSM-LEP-100554; Yarinal], (MUSM), 25 Jul 2001, 1 ♂ [MUSM-LEP-100500; varillal seco], (MUSM); *Madre de Dios*: 0–2 km W Puerto Maldonado, [12°36'12"S,69°12'32"W], 250 m, (Miller, L. D.), 14 Aug 1981, 1 ♂ [FLMNH-MGCL-263367], (FLMNH); 12 km N Tambopata, Comunidad Infierno, [12°43'56"S,69°13'44"W], 200 m, (Harris, L. & H.), 18 Jun 1985, 1 ♂ [FLMNH-MGCL-264647], (FLMNH); 15 km NE Puerto Maldonado, [12°29'52"S,69°8'41"W], 200 m, (Lamas, G.), 19 Jun 1989, 1 ♂ [MUSM-LEP-100511], (MUSM), 9 Jun 1989, 1 ♂ [MUSM-LEP-100510], (MUSM), (Medina, M.), 11 Feb 1990, 1 ♂ [MUSM-LEP-100512], (MUSM); Boca Río La Torre, [12°50'S,69°17'W], 300 m, (Lamas, G.), 20 Feb 1982, 1 ♂ [MUSM-LEP-100508], (MUSM), 22 Oct 1982, 1 ♂ [MUSM-LEP-100509], (MUSM), 24 Oct 1983, 1 ♂ [MUSM-LEP-100506], (MUSM), 26 Jul 1980, 1 ♀ [MUSM-LEP-100557], (MUSM), 4 Mar 1983, 1 ♂ [MUSM-LEP-100507], (MUSM), 7 Oct 1983, 1 ♀ [MUSM-LEP-100556], (MUSM); Cerro Pantiacolla, E slope, 4–5 km ENE Shintuya, [12°38'S,71°16'W], 1000 m, (Douglass, J. F.), 19 Jul 1980, 1 ♂ [FLMNH-MGCL-263346; 1159], (FLMNH); Cerro Pantiacolla, E slope, 4-5 km ENE Shintuya, [12°38'S,71°16'W], 960–1120 m, (Douglass, J. F.), 23 Jul 1980, 1 ♂ [FLMNH-MGCL-263366; 1244], (FLMNH); Madre de Dios, [12°16'S,70°55'W], 491 m, (Brock, J. P.), 15 May 2012, 1 ♂ [FLMNH-MGCL-1036216], (FLMNH); Parque Manu, Pakitza, [11°55'48"S,71°15'18"W], 340 m, (Clarke, N. L.), 21 Apr 1991, 1 ♂ [MUSM-LEP-100518], (MUSM); Parque Manu, Pakitza, [11°55'48"S,71°15'18"W], 340–400 m, (DZUP); Parque Manu, Pakitza, [11°55'48"S,71°15'18"W], 400 m, (Lamas, G.), 11 Oct 1990, 1 ♂ [MUSM-LEP-100515], (MUSM), 8-14 Sep 1989, 1 ♂ [MUSM-LEP-100513], (MUSM), (Lozada, P.), 14 Feb 1992, 1 ♂ [MUSM-LEP-100514], (MUSM), (Rowe, W.), 13 Nov 1990, 1 ♂ [MUSM-LEP-100516], (MUSM), 3 Nov 1990, 1 ♂ [MUSM-LEP-100517], (MUSM); Parque Nacional Manu, Cocha Cashu, [11°53'S,71°25'W], 350 m, (Lilleengen, P.), (collection unknown) (Lilleengen, P., pers. comm. (email to KRW 29 Sep 2016)); Refugio Juliaca, [12°57'S,68°53'W], 220 m, (Lamas, G.), 18 Jun 1992, 1 ♀ [MUSM-LEP-100559; bosque], (MUSM); Reserva de Tambopata, [12°50'S,69°17'W], 250 m, (DZUP); Río Madre de Dios, Albergue Amazonia, [12°52'S,71°23'W], 500 m, (Brock, J.), 29 Sep 2011, 1 ♂ [MUSM-LEP-100526], (MUSM), (Lamas, G.), 27 Sep 2011, 1 ♀ [MUSM-LEP-100561], (MUSM); 'Sheringayoc bis Pto. Maldonado', (Koepcke, H:W.), 16–18 May 1949, 1 ♂, (ZSM); *Pasco*: Chuchurras, [10°9'S,75°14'W], 300 m, (Martin, P.), 3 ♂, 1 ♀, (CMNH); near Eneñas, 1400 m, (Hocking, P.), 9 Oct 1967, 1 ♂ [MUSM-LEP-100469], 1 ♂ [MUSM-LEP-100470], (MUSM); P. N. Yanachaga Chemillén, Pampa Pescado, [10°22'33"S,75°14'36"W], 400-450 m, (Carbonel, S.), 18 Sep 2007, 1 ♀ [MUSM-LEP-100539], (MUSM), (Grados, J., Carbonel, S.), 18 Sep 2007, 1 ♀ [MUSM-LEP-100541], (MUSM); P.N. Yanachaga-Chemillén, Huampal, [10°11'S,75°34'W], 1050 m, (Peña, C.), 9 Feb 2003, 1 ♂ [MUSM-LEP-100473], (MUSM); Parque Nacional Yanachaga – Chemillén, Paujil, [10°20'S,75°16'W], 375 m, (Carbonel, S., Calderón C.), 22 May 2008, 1 ♂ [MUSM-LEP-100471], (MUSM), (Grados, J., Calderón, C., Carbonel S.), 22 May 2008, 1 ♀ [MUSM-LEP-100540], (MUSM); Pozuzo, [10°7'S,75°32'W], 800 m, 1 ♂ [FLMNH-MGCL-263362], (FLMNH); Río Palcazu, Río Chuchurras, [10°7'S,75°10'W], 320 m, 1 ♂ [FLMNH-MGCL-263353], 1 ♂ [FLMNH-MGCL-263360], 1 ♂ [FLMNH-MGCL-263368], 1 ♀ [FLMNH-MGCL-263376], (FLMNH); Río Palcazu, Río Chuchurras, [10°9'S,75°14'W], 320 m, (Hoffmanns, W.), 1 ♂ [BMNH(E)#1498907], 1 ♂ [BMNH(E)#1498908], 1 ♂ [BMNH(E)#1498909], 1 ♂ [BMNH(E)#1498910], 1 ♂ [BMNH(E)#1498911], (NHMUK); San Juan de Cacazú, [10°40'S,75°7'W], 1600 m, (Peña, C.), 21 Oct 2002, 1 ♂ [MUSM-LEP-100472], (MUSM); *Pasco*: Río Tambopata, Z. R. Tambopata-Candamo, [13°22'S,69°34'W], 270 m, (Cambridge University Amazon Expedition), 28 Jul 1995, 1 ♀ [BMNH(E)#1420186], (NHMUK), 8 Aug 1995, 1 ♂ [BMNH(E)#1420179], (NHMUK); Río Tambopata, Z. R. Tambopata-Candamo, [13°22'S,69°34'W], 300 m, (Lamas, G.), 25 Oct 1990, 1 ♀ [MUSM-LEP-100558], (MUSM); Yahuarmayo, 366 m, (Watkins, H. & C.), Feb–Mar 1912, 1 ♂ [BMNH(E)#1498929], 1 ♂ [BMNH(E)#1498930], (NHMUK); *San Martín*: 5 km S Moyobamba, nr. Baños Termales, [6°4'31"S,76°58'6"W], 970 m, (Nakamura, I.), 17 Oct 2012, (ICNA); 5 km S Moyobamba, nr. Baños Termales, [6°4'31"S,76°58'6"W], 970-1120 m, (Nakamura, I.), 14 Oct 2012, 1 ♀, (ICNA); Moyobamba rd. nr. km 487, N side of Calzada, 830 m, (Nakamura, I.), 16 Oct 2012, (ICNA); Moyobamba, Rumipata, (Elias, M.), 26 Oct 2015, 1 ♂ [FLMNH-MGCL-209944], (FLMNH); Río Serranoyacu, [5°40'S,77°40'W], 1200 m, (Lamas, G.), 13 Mar 1985, 1 ♂ [MUSM-LEP-100463], 1 ♂ [MUSM-LEP-100464], (MUSM); Rumi Jepelacio, Ruta del Agua, (Elias, M.), 26 Oct 2015, 1 ♂ [FLMNH-MGCL-209949], (FLMNH), 27 Oct 2015, 1 ♀ [FLMNH-MGCL-209937], (FLMNH); *Ucayali*: Pucallpa, [8°23'S,74°37'W], 150 m, (Schunke, J. M.), 4 Dec 1974, 1 ♂ [MUSM-LEP-100505], (MUSM); Pucallpa, [8°23'S,74°37'W], 180-182 m, (Jae, R.), 22 Jul 1964, 1 ♂ [FLMNH-MGCL-263364], (FLMNH), 26 May 1962, 1 ♂ [FLMNH-MGCL-263352], (FLMNH); Río Aguaytía, Previsto, [9°3'S,75°38'W], 420-500 m, (Knudson, E. C.), Oct 2006, 1 ♂ [FLMNH-MGCL-1036225], (FLMNH); *Not located*: ‘Río Huallaga’, [5°7'S,75°37'W], 300 m, (Heppner, J. B., Lamas, G.), 16 Oct 2012, 1 ♂ [FLMNH-MGCL-1036214], (FLMNH); ‘N Peru’, (Krause), 1 ♂ [BMNH(E)#1420182], (NHMUK); no data, 1 ♂ [BMNH(E)#1420183], (NHMUK); Siberia, (DZUP). **Suriname**: *Brokopondo*: Berg-en-Dal, [5°9'N,55°4'W], (Ellacombe, G. W.), Apr 1892, 1 ♂ [BMNH(E)#1498875], 1 ♂ [BMNH(E)#1498876], (NHMUK); Brownsberg, rainforest km 6-12, [4°56'36"N,55°10'15"W], (Pellmyr, O.), 30 Jan 1982, 1 ♂ [NT *arnaca*], (USNM); Saramacca River, [5°31'N,54°2'W], May 1893, 1 ♂ [BMNH(E)#1498874], (NHMUK); *Marowijne*: Cottica River, Moengo, [5°37'33"N,54°24'29"W], May 1927, 2 ♂, (CMNH); *Para*: Bersaba, [5°32'N,55°3'W], (Michael), 1898-1899, 1 ♂, (MNHU); *Paramaribo*: No specific locality, (Ellacombe, G. W.), Feb 1892, 1 ♂ [BMNH(E)#1420091], (NHMUK); *Sipaliwini*: Alalapadu, 300 m, (Simon, M. J.), Mar 2002, 1 ♂ [FLMNH-MGCL-262966], (FLMNH); *Not located*: ‘Surinam’, 1 ♂ [BMNH(E)#1420092], 1 ♂ [BMNH(E)#1420093], 1 ♂ [BMNH(E)#1420094], 1 ♂ [BMNH(E)#1420095], 1 ♀ [BMNH(E)#1420096], 1 ♀ [BMNH(E)#1420097], 1 ♀ [BMNH(E)#1420098], (NHMUK), 2 ♂, 2 ♀, (MNHU). **Trinidad & Tobago**: *Diego Martin*: Fort George, [10°42'N,61°32'W], Sep 1891, 1 ♂ [BMNH(E)#1498885], 1 ♂ [BMNH(E)#1498886], (NHMUK); Mt. Catherine upper trail, (Preston, J. & F.), 29 Apr 1982, 1 ♀ [FLMNH-MGCL-257158], (FLMNH); *Port of Spain*: Port of Spain, [10°40'N,61°31'W], 17–23 Aug 1965, 1 ♀ [FLMNH-MGCL-263057], (FLMNH); *San Juan-Laventille*: Fondes Amandes, 91 m, 16 Apr 1922, 1 ♂ [FLMNH-MGCL-263109], (FLMNH), 16 Apr 1922, 1 ♀ [FLMNH-MGCL-263129], (FLMNH); Maraval, [10°43'N,61°31'W], Jul 1891, 1 ♂ [BMNH(E)#1498860], 1 ♂ [BMNH(E)#1498861], (NHMUK); *Siparia*: Parrylands, [10°11'N,61°37'W], 19 Aug 1974, 1 ♂ [FLMNH-MGCL-257157], (FLMNH); *Tunapuna-Piarco*: 6 mi. N Arima, [10°42'12"N,61°17'28"W], 300 m, (Pliske, T. E.), 30 Jul 1962, 1 ♂ [FLMNH-MGCL-263116], (FLMNH), 5 Jul 1962, 1 ♂ [FLMNH-MGCL-263111], (FLMNH), 6 Aug 1962, 1 ♂ [FLMNH-MGCL-263110], (FLMNH); 7.5 mi N Arima, Asa Wright Nature Center, [10°43'3"N,61°17'55"W], 488 m, (Klein, T. W.), 3 Sep 2000, 1 ♂ [FLMNH-MGCL-257155], (FLMNH); Arima rd., [10°42'36"N,61°17'32"W], (S. K.), 1 ♀ [FLMNH-MGCL-263134], (FLMNH); Maracas Valley, [10°42'37"N,61°24'54"W], 100 m, (Birch, F.), Aug 1905, 1 ♂ [BMNH(E)#1498869], (NHMUK); *Not located*: ‘Trinidad’, 1 ♂ [BMNH(E)#1420079], 1 ♂ [BMNH(E)#1420080], 1 ♂ [BMNH(E)#1498866], 1 ♂ [BMNH(E)#1498867], 1 ♂ [BMNH(E)#1498868], (NHMUK), 1 ♂ [FLMNH-MGCL-263113], (FLMNH), (Fountaine, M.), Dec 1911, 1 ♂ [BMNH(E)#1420081], (NHMUK), (Kaye, W.J.), 1898, 1 ♂ [FLMNH-MGCL-295862], (FLMNH), (Simon, M. J.), 16 Apr 1922, 1 ♂ [FLMNH-MGCL-263104], (FLMNH); Port of Spain, (Birch, F.), 1 ♂ [BMNH(E)#1498865], (NHMUK); Sangre Grande, Sans Souci Estate, (Church, C.), 1 ♂ [FLMNH-MGCL-257154], (FLMNH); St. George, Sep 1891, 1 ♂ [BMNH(E)#1498884], (NHMUK), (Ellacombe, G. W.), Dec 1891, 1 ♂ [BMNH(E)#1498877], 1 ♂ [BMNH(E)#1498878], 1 ♂ [BMNH(E)#1498879], 1 ♂ [BMNH(E)#1498880], 1 ♂ [BMNH(E)#1498881], 1 ♂ [BMNH(E)#1498882], 1 ♂ [BMNH(E)#1498883], (NHMUK); 'St. George', (Ellacombe, G. W.), 1 ♂ [BMNH(E)#1420076], 1 ♂ [BMNH(E)#1420077], 1 ♀ [BMNH(E)#1420078], (NHMUK); Stanway Parris River, Feb 1921, 1 ♂ [BMNH(E)#1498862], 1 ♂ [BMNH(E)#1498863], 1 ♂ [BMNH(E)#1498864], (NHMUK). **Venezuela**: *Amazonas*: Caño Wiraki, 400 m, (Lichy, R.), 30 Apr 1950, 1 ♂ [FLMNH-MGCL-263108], (FLMNH); *Bolívar*: 80 km S El Dorado, [6°11'8"N,61°24'36"W], 900 m, (O'Shea, G.), 28 Jun 1984, 1 ♂ [FLMNH-MGCL-264663; Sta.102], 1 ♂ [FLMNH-MGCL-264664; Sta.102], 1 ♂ [FLMNH-MGCL-264665; Sta.102], 1 ♀ [FLMNH-MGCL-264668; Sta.102], (FLMNH); 85 km S El Dorado on mining road near Las Claritas Hotel, (Denno, R. F.), 3 Nov 1982, 1 ♂ [FLMNH-MGCL-262944], (FLMNH), 5 Nov 1982, 1 ♂ [FLMNH-MGCL-262942], 1 ♂ [FLMNH-MGCL-262943], (FLMNH); El Dorado, km 82, [6°10'54"N,61°25'6"W], (Harris, L. & H.), 27 Jun 1984, 1 ♂ [FLMNH-MGCL-262945], (FLMNH); *Mérida*: Azulita to Mirabal, 1460 m, (Huber, H.), 10 Apr 1971, 1 ♂, 1 ♀, (ZSM); between Masa Bolívar and La Victoria, 800 m, (Huber, H.), 23 May 1971, 1 ♀, (ZSM); 'La Victoria to Estanquas', [8°26'29"N,71°33'56"W], 560 m, (Huber, H.), 19 Apr 1971, 3 ♂, (ZSM); Santa Cruz de Mora, 620 m, (Huber, H.), 30 Jan 1971, 1 ♀, (ZSM); *Monagas*: near San Francisco stream, 2 km E of Rt. 1 on Caripe rd., 1000 m, (Hedbor, J. D. & E. R), 2 Feb 1994, 1 ♂ [FLMNH-MGCL-264682; daytime bait fruit trap], (FLMNH); *Táchira*: Chimborazo, 10 May 1937, 1 ♂ [FLMNH-MGCL-263107], (FLMNH); La Fría, [8°13'N,72°15'W], 130 m, (Jenkins, D. W. & J.), 25 Apr 1982, 1 ♀ [FLMNH-MGCL-264685], (FLMNH); Palmira, San Cristobal, [7°46'N,72°14'W], 1000 m, (Hocking, P.), 27 Dec 1979, 1 ♂ [MUSM-LEP-100437], (MUSM); Palmira, San Cristobal, [7°46'N,72°14'W], 1300 m, (Hocking, P.), 27 Dec 1979, 1 ♂ [MUSM-LEP-100438], (MUSM). **Country unknown**: *Not located*: 'Amaz[on]', 1 ♂ [BMNH(E)#1498941], (NHMUK), (Mathan, M. de), Apr 1905, 1 ♂ [BMNH(E)#1498942], (NHMUK); 'Amazon', 1 ♂ [BMNH(E)#1420199], (NHMUK), 1 ♂, (CMNH); 'Amazonas', (Fassl, A. H.), 21 ♂, 2 ♀, (ZSM); no data, 1 ♂ [BMNH(E)#1420192], 1 ♂ [BMNH(E)#1420193], 1 ♂ [BMNH(E)#1420200], 1 ♂ [BMNH(E)#1420203], 1 ♂ [BMNH(E)#1498943], 1 ♂ [BMNH(E)#1498944], 1 ♂ [BMNH(E)#1498945], 1 ♂ [BMNH(E)#1498946], 1 ♂ [BMNH(E)#1498947], 1 ♂ [BMNH(E)#1498948], 1 ♂ [BMNH(E)#1498949], 1 ♂ [BMNH(E)#1498950], 1 ♂ [BMNH(E)#1498954], 1 ♂ [BMNH(E)#1498958], 1 ♂ [BMNH(E)#1498959], 1 ♂ [BMNH(E)#1498960], 1 ♂ [BMNH(E)#1498964], 1 ♂ [BMNH(E)#1670215], 1 ♀ [BMNH(E)#1498952], 1 ♀ [BMNH(E)#1498953], (NHMUK), 1 ♀ [FLMNH-MGCL-263135], 1 ♀ [FLMNH-MGCL-263379], (FLMNH), 1 ♀, (MNHU), 2 ♂, (ZSM), 1 Jul, 1 ♂ [BMNH(E)#1498963], (NHMUK), 13 Aug 1993, 1 ♂ [FLMNH-MGCL-263052], (FLMNH), 23 Jun, 1 ♂ [BMNH(E)#1498957], (NHMUK), 5 Sep, 1 ♂ [BMNH(E)#1498956], (NHMUK), 7 Aug, 1 ♂ [BMNH(E)#1498955], (NHMUK), 1 ♀ ['Pap. arnaca, Fabr. pag 75 No 331'], 21 Mar 1991, 1 ♀ [FLMNH-MGCL-263056], (FLMNH), (Mckenzie), 29 Jul 1969, 1 ♂ [BMNH(E)#1498961], (NHMUK), 30 Jul 1969, 1 ♂ [BMNH(E)#1498962], (NHMUK); 'South America', 1 ♀ [BMNH(E)#1420198], (NHMUK); 'upper Amazon', 1 ♀, (CMNH).

**Other records**: **Bolivia**: *El Beni*: Bosque Chimanes, Jamanchi, [15°23'42"S,67°15'20"W], (Guerra, F., Gironda, W.), 7-15 Nov 1993, 1 ♂ [CBF-927], (CBF) (DABD of CBF). **Colombia**: *Caquetá*: Belén de los Andaquies, La Cerinda, [1°37'3"N,75°51'57"W], 680 m, (Sañudo, C.), 4 Oct 07, 1 ♂/♀ [MEFLG-14974; jama], (MEFLG); Belén de los Andaquies, Reserva Páez-La Esperanza, [1°32'16"N,75°57'11"W], 1048 m, (Sañudo, C.), 3 Dec 2007, 1 ♂/♀ [MEFLG-15095; jama], (MEFLG); El Quince, Parcela La Simbra, [0°48'11"N,75°11'58"W], 204 m, (Sañudo, C., Muñoz, F.), 4 Sep 2007, 1 ♂/♀, (MEFLG) (Claudia Sañudo, pers. comm.); La Cerinda, camino Casa Ramiro Tascon, [1°37'3"N,75°51'57"W], 680 m, (Sañudo, C., Muñoz, F.), 6 Oct 2007, 1 ♂/♀ (HERENCIA) (Claudia Sañudo, pers. comm.); La Cerinda, Laguna camino Andaqui, [1°37'18"N,75°50'4"W], 894 m, (Sañudo, C., Muñoz, F.), 3 Oct 2007, 1 ♂/♀ (MUA) (Claudia Sañudo, pers. comm.); La Esperanza-PNNAFIW, camino Casa Celio Usme, [1°32'15"N,75°57'11"W], 1036 m, (Sañudo, C.), 3 Dec 2007, 1 ♂/♀ (HERENCIA) (Claudia Sañudo, pers. comm.); *Meta*: Villavicencio, Bavaria, [4°10'44"N,73°38'58"W], 570 m, (Marín, M. A.), 13-Oct-06, 1 ♂/♀ [MEFLG-15498; jama], 1 ♂/♀ [MEFLG-15499; jama], 1 ♂/♀ [MEFLG-15500; jama], 1 ♂/♀ [MEFLG-15501; jama], 1 ♂/♀ [MEFLG-15502; jama], (MEFLG). **Ecuador**: *Morona-Santiago*: Gualaquiza, [3°25'S,78°36'W], 1500 m, (Piñas, F.), 25 Jul 1995, 1 ♂/♀ [FDPR-18183], 1 ♂/♀ [FDPR-18190], (FRPI) (DABD of FRPI); Indanza, [3°3'30"S,78°28'40"W], 1500 m, (Piñas, F.), 29 Apr 1995, 1 ♂/♀ [FDPR-16682], 1 ♂/♀ [FDPR-18184], 1 ♂/♀ [FDPR-18214], (FRPI) (DABD of FRPI); km 40 Méndez-Santiago rd., [2°54'38"S,78°14'51"W], 750 m, (Willmott, K. R.), 2 Nov 1996, (W&H); Macas, Loma Kilamo, [2°18'18"S,78°8'42"W], 1470 m, (Willmott, K. R.), 7 Dec 2003, (W&H); nr. Gualaquiza, Bomboiza, [3°25'36"S,78°31'W], 800-850 m, (Hall, J. P. W., Willmott, K. R.), 26-29 Jul 1993, (W&H), 26-29 May 1994, (W&H), 5,6 Nov 1996, (W&H); San Juan Bosco, [3°5'55"S,78°33'9"W], 1500 m, (Estévez, G.), 1 Aug 1992, 1 ♂/♀ [MECN-TABDP-19014], (INABIO) (DABD of INABIO); Tayuza-Yakunk trail, [2°44'26"S,78°12'14"W], 850 m, (Willmott, K. R.), 1 Dec 2003, (W&H); Yakunk-Cutucú trail, lower ridge, [2°45'40"S,78°9'40"W], 1340 m, (Willmott, K. R.), 3 Dec 2003, (W&H); Yakunk-Cutucú trail, river camp, [2°45'7"S,78°10'55"W], 1000 m, (Willmott, K. R.), 2 Dec 2003, (W&H); *Napo*: km 14 Tena-Puyo rd., Apuya, [1°6'18"S,77°46'42"W], 600 m, (Piñas, F.), 12 Oct 2006, 1 ♂/♀ [FDPR-16708], 1 ♂/♀ [FDPR-18145], (FRPI), 13 Dec 2006, 1 ♂/♀ [FDPR-16676], (FRPI), 13 Jul 2005, 1 ♂/♀ [FDPR-16667], 1 ♂/♀ [FDPR-18178], (FRPI), 19 Jul 2006, 1 ♂/♀ [FDPR-16669], 1 ♂/♀ [FDPR-16670], 1 ♂/♀ [FDPR-16671], 1 ♂/♀ [FDPR-16675], (FRPI), 20 Oct 2006, 1 ♂/♀ [FDPR-16697], 1 ♂/♀ [FDPR-18117], (FRPI), 21 Nov 2006, 1 ♂/♀ [FDPR-18195], 1 ♂/♀ [FDPR-18205], (FRPI), 25 Jan 2002, 1 ♂/♀ [FDPR-16704], 1 ♂/♀ [FDPR-18106], 1 ♂/♀ [FDPR-18109], (FRPI), 26 Dec 2006, 1 ♂/♀ [FDPR-16649], 1 ♂/♀ [FDPR-16685], (FRPI), 27 Sep 2006, 1 ♂/♀ [FDPR-16694], 1 ♂/♀ [FDPR-16696], 1 ♂/♀ [FDPR-16698], (FRPI), 29 Sep 2006, 1 ♂/♀ [FDPR-16699], (FRPI), 7 Nov 2006, 1 ♂/♀ [FDPR-18156], 1 ♂/♀ [FDPR-18166], 1 ♂/♀ [FDPR-18175], 1 ♂/♀ [FDPR-18185], (FRPI), 9 Jan 2007, 1 ♂/♀ [FDPR-18101], 1 ♂/♀ [FDPR-18102], (FRPI), (Willmott, K. R., Hall, J. P. W.), 04 May 1994, (W&H), 10 Sep 1996, (W&H), 12-14,24 Feb 1995, (W&H), 28 Aug 1997, (W&H), 6,10,13,19,21,28 Oct 1996, (W&H), Dec, (W&H); km 49 Tena-Loreto rd., [0°42'51"S,77°44'26"W], 1300 m, (Willmott, K. R., Hall, J. P. W.), Mar, (W&H), Oct, (W&H); Puyo-Tena rd., Santa Clara, [1°15'30"S,77°53'W], 700 m, (Piñas, F.), 11 Jan 2002, 1 ♂/♀ [FDPR-18103], 1 ♂/♀ [FDPR-18199], (FRPI), 7 Dec 2001, 1 ♂/♀ [FDPR-16690], (FRPI) (DABD of FRPI); Río Jatunyacu, Pimpilala, [1°4'31"S,77°56'13"W], 600-650 m, (Willmott, K. R., Hall, J. P. W.), 15,16 Feb 1995, (W&H), Sep, (W&H); Río Misahuallí, Misahuallí, [1°1'36"S,77°40'W], 500 m, (Piñas, F.), 2 Feb 2002, 1 ♂/♀ [FDPR-16641], 1 ♂/♀ [FDPR-18197], (FRPI), 4 Feb 2003, 1 ♂/♀ [FDPR-16637], 1 ♂/♀ [FDPR-18115], 1 ♂/♀ [FDPR-18160], 1 ♂/♀ [FDPR-18165], (FRPI) (DABD of FRPI); Río Napo, Puerto Napo, [1°3'S,77°47'W], 500 m, (Piñas, F.), 14 Jul 2005, 1 ♂/♀ [FDPR-18182], 1 ♂/♀ [FDPR-18187], 1 ♂/♀ [FDPR-18188], (FRPI), 17 May 2006, 1 ♂/♀ [FDPR-18146], (FRPI), 18 Jul 2006, 1 ♂/♀ [FDPR-16674], (FRPI), 28 May 2004, 1 ♂/♀ [FDPR-16652], 1 ♂/♀ [FDPR-16653], 1 ♂/♀ [FDPR-16654], 1 ♂/♀ [FDPR-16655], 1 ♂/♀ [FDPR-16656], 1 ♂/♀ [FDPR-16663], 1 ♂/♀ [FDPR-16664], 1 ♂/♀ [FDPR-16665], 1 ♂/♀ [FDPR-16666], (FRPI), 29 Aug 2003, 1 ♂/♀ [FDPR-16657], (FRPI), 4 Jul 2003, 1 ♂/♀ [FDPR-18167], (FRPI), 4 Nov 2003, 1 ♂/♀ [FDPR-18111], 1 ♂/♀ [FDPR-18116], 1 ♂/♀ [FDPR-18126], 1 ♂/♀ [FDPR-18131], (FRPI) (DABD of FRPI); Río Napo, Puerto Napo, [1°3'S,77°47'W], 600 m, (Piñas, F.), 15 Sep 2005, 1 ♂/♀ [FDPR-16650], 1 ♂/♀ [FDPR-16659], 1 ♂/♀ [FDPR-16660], 1 ♂/♀ [FDPR-16661], 1 ♂/♀ [FDPR-16662], (FRPI) (DABD of FRPI); Río Napo, Puerto Napo-Ahuano rd., Chichicorrumi, [1°4'11"S,77°37'45"W], 450 m, (Willmott, K. R., Hall, J. P. W.), 4 Dec 1997, Aug, Jul, Sep, (W&H); Río Napo, Puerto Napo-Ahuano rd., Jatun Sacha, [1°3'S,77°35'9"W], 450 m, (Murray, D.), 8 Aug 1990, 1 ♂/♀ [MECN-TABDP-19015], (INABIO) (DABD of INABIO); Río Pano, [1°0'48"S,77°51'45"W], 600 m, (Piñas, F.), 13 Jun 2006, 1 ♂/♀ [FDPR-16672], 1 ♂/♀ [FDPR-16673], (FRPI) (DABD of FRPI); Río Sinde, km 12 Tena-Puyo rd., Finca San Carlo, [1°5'18"S,77°47'24"W], 600 m, (Willmott, K. R., Hall, J. P. W.), 19 Aug 1997, (W&H), 20-22 Feb 1995, (W&H), 5 Dec 1997, (W&H), Sep, (W&H); Río Urcusiqui, Baeza-Tena rd., Sarayacu, [0°40'30"S,77°49'12"W], 1300-1400 m, (Willmott, K. R., Hall, J. P. W.), 3 Mar 1995, (W&H); San Rafael, [0°6'15"S,77°35'12"W], 1150 m, (Piñas, F.), 17 Apr 2000, 1 ♂/♀ [FDPR-16668], (FRPI) (DABD of FRPI); San Rafael, [0°6'15"S,77°35'12"W], 1550 m, (Oña, P.), 27 May 2000, 1 ♂/♀ [FDPR-18209], (FRPI), (Piñas, F.), 18 Jun 1998, 1 ♂/♀ [FDPR-16643], 1 ♂/♀ [FDPR-18173], 1 ♂/♀ [FDPR-18193], (FRPI), 7 Sep 1999, 1 ♂/♀ [FDPR-18138], 1 ♂/♀ [FDPR-18139], 1 ♂/♀ [FDPR-18198], (FRPI) (DABD of FRPI); Serena, [1°5'25"S,77°55'33"W], 600 m, (Piñas, F.), 11 Oct 2006, 1 ♂/♀ [FDPR-16709], (FRPI) (DABD of FRPI); Tena-Loreto rd., Río Hollín, [0°41'48"S,77°43'39"W], 800 m, (Piñas, F.), 1 Sep 2000, 1 ♂/♀ [FDPR-18158], 1 ♂/♀ [FDPR-18211], (FRPI) (DABD of FRPI); Tena-Puyo rd., El Capricho, [1°11'14"S,77°49'53"W], 800 m, (Willmott, K. R.), 26 Oct 1996, (W&H); *Orellana*: Coca-Tiguino rd., Río Tiputini, [0°44'3"S,76°53'31"W], 300 m, (Willmott, K. R., Hall, J. P. W.), 20 Sep 1996, (W&H), 30 Jun 1994, (W&H); km 21 Coca-Loreto rd., [0°29'31"S,77°8'19"W], 300 m, (Willmott, K. R., Hall, J. P. W.), 08 Mar 1995, (W&H); Lagunas de Cuyabeno, La Ormiga, [0°1'S,76°11'W], 250 m, (Willmott, K. R.), 18 Sep 1996, (W&H); Reserva Biológica del Río Bigal, [0°31'17"S,77°25'10"W], 851 m, (Violette), 23 Jul 2010, 1 ♂/♀ [N82], (FLMNH) (CULEPEX Expedition, 2010); Reserva Biológica del Río Bigal, [0°31'24"S,77°25'9"W], 891 m, (Violette), 22 Jul 2010, 1 ♂/♀ [N28], (FLMNH) (CULEPEX Expedition, 2010); Reserva Biológica del Río Bigal, [0°31'28"S,77°25'4"W], 922 m, (Violette), 22 Jul 2010, 1 ♂/♀ [N24], (FLMNH) (CULEPEX Expedition, 2010); Reserva Biológica del Río Bigal, [0°31'32"S,77°25'3"W], 922 m, (Radford, J.), 22 Jul 2010, 1 ♂/♀ [N46], (FLMNH) (CULEPEX Expedition, 2010); Reserva Biológica del Río Bigal, 'the Hooch', [0°31'25"S,77°25'7"W], 892 m, (Hartley, E.), 22 Jul 2010, 1 ♂/♀ [N31], (FLMNH) (CULEPEX Expedition, 2010); Río Manduro, Río Napo, Yarina, [0°28'14"S,76°50'W], 300 m, (Willmott, K. R.), 17 Nov 2003, (W&H), 23-24 Jul 1998, (W&H); Río Napo, Boca del Río Añangu, [0°31'43"S,76°23'41"W], 220–300 m, 26 Oct 2005, (W&H); Río Napo, Pañacocha, [0°26'11"S,76°4'W], 250 m, 15,16,20 Oct 1997, (W&H); Río Napo, Río Yuturi, lodge trail, [0°32'53"S,76°2'W], 250 m, (Willmott, K. R.), 18 Nov 2003, (W&H), 19 Nov 2003, (W&H); Río Napo, Río Yuturi, Río Manduro trail, [0°33'29"S,76°2'39"W], 250 m, (Willmott, K. R.), 20 Nov 2003, (W&H); Río Napo, Sacha Lodge, [0°28'14"S,76°27'33''w], 200 m, (Hall, J. P. W.), 17,18 Feb 2001, (W&H); Río Tiguino, Tiguino, [1°6'35"S,76°56'45"W], 350 m, (Willmott, K. R.), 23, 24 Oct 1997, (W&H); Río Tiputini, Estación Científica Yasuní, parcela 50 Ha, [0°40'55"S,76°24'1"W], 250–300 m, (Mena, S.), 2017, 3 ♂, (PUCE) (Checa, M. (pers. comm. Oct 2017, email to KRW with photograph)); Río Tiputini, Tiputini Biodiversity Station, [0°42'12"S,76°0'30"W], 200 m, (Hall, J. P. W.), 10-15 Aug 2002, (FLMNH) (W&H); Río Tiputini, vía Auca, Estación Científica Yasuní, [0°40'27"S,76°23'49"W], 220–250 m, (Willmott, K. R., Hall, J. P. W.), 16–18 Aug 1999, (W&H); Yasuní, Estación Científica, [0°40'17"S,77°24'2"W], 250 m, (Piñas, F.), 29 Sep 1995, 1 ♂/♀ [FDPR-18133], (FRPI) (DABD of FRPI); *Pastaza*: ENE of Mera, Río Puyo, [1°25'42"S,78°2'48"W], 1300 m, (Willmott, K. R., Hall, J. P. W.), 3 Oct 1996, (W&H); km 25 Puyo-Tena rd., [1°19'42"S,77°56'W], 900–1000 m, (Willmott, K. R., Hall, J. P. W.), 7 Dec 1996, (W&H); km 35 Puyo-Tena rd., [1°16'48"S,77°51'48"W], 1000 m, (Willmott, K. R.), 4 Oct 1996, (W&H); Mera, [1°28'S,78°6'W], 1100 m, (Piñas, F.), 20 Oct 2001, 1 ♂/♀ [FDPR-18141], (FRPI), 31 Aug 2001, 1 ♂/♀ [FDPR-16691], (FRPI) (DABD of FRPI); Puyo, [1°28'S,77°59'W], 1000 m, (Piñas, F.), 12 Oct 2001, 1 ♂/♀ [FDPR-18122], 1 ♂/♀ [FDPR-18135], (FRPI), 21 Sep 2001, 1 ♂/♀ [FDPR-16644], (FRPI), 29 Dec 2001, 1 ♂/♀ [FDPR-18123], 1 ♂/♀ [FDPR-18124], 1 ♂/♀ [FDPR-18125], (FRPI), 31 Aug 2001, 1 ♂/♀ [FDPR-18142], 1 ♂/♀ [FDPR-18143], (FRPI), 8 Mar 2002, 1 ♂/♀ [FDPR-16681], 1 ♂/♀ [FDPR-16688], 1 ♂/♀ [FDPR-18104], 1 ♂/♀ [FDPR-18134], 1 ♂/♀ [FDPR-18210], (FRPI), 9 Aug 2001, 1 ♂/♀ [FDPR-16677], 1 ♂/♀ [FDPR-16684], 1 ♂/♀ [FDPR-18128], 1 ♂/♀ [FDPR-18132], 1 ♂/♀ [FDPR-18136], 1 ♂/♀ [FDPR-18147], 1 ♂/♀ [FDPR-18177], 1 ♂/♀ [FDPR-18194], 1 ♂/♀ [FDPR-18196], (FRPI) (DABD of FRPI); Puyo-Macas rd., Pitirishca, [1°48'18"S,77°49'15"W], 1000 m, (Willmott, K. R., Hall, J. P. W.), 26 Jul 1998, (W&H); Río Pastaza, Kapawi village, [2°32'16"S,76°50'10"W], (Willmott, K. R., Hall, J. P. W.), 23 Jul 2009, (W&H); Río Pindo Grande, Shell, [1°29'40"S,78°3'40"W], 1050 m, (Piñas, F.), 25 Aug 2001, 1 ♂/♀ [FDPR-16705], 1 ♂/♀ [FDPR-18180], (FRPI) (DABD of FRPI); Río Pindo Grande, Shell, [1°29'40"S,78°3'40"W], 1150 m, (Piñas, F.), 25 Aug 2001, 1 ♂/♀ [FDPR-18171], (FRPI) (DABD of FRPI); San Ramón, 900 m, (Piñas, F.), 16 Nov 2001, 1 ♂/♀ [FDPR-16658], 1 ♂/♀ [FDPR-16700], 1 ♂/♀ [FDPR-16702], 1 ♂/♀ [FDPR-18113], 1 ♂/♀ [FDPR-18114], 1 ♂/♀ [FDPR-18118], 1 ♂/♀ [FDPR-18121], 1 ♂/♀ [FDPR-18204], (FRPI), 30 Nov 2001, 1 ♂/♀ [FDPR-16647], 1 ♂/♀ [FDPR-16648], 1 ♂/♀ [FDPR-16679], 1 ♂/♀ [FDPR-16703], 1 ♂/♀ [FDPR-18105], 1 ♂/♀ [FDPR-18107], 1 ♂/♀ [FDPR-18108], 1 ♂/♀ [FDPR-18110], 1 ♂/♀ [FDPR-18112], 1 ♂/♀ [FDPR-18119], 1 ♂/♀ [FDPR-18120], 1 ♂/♀ [FDPR-18127], 1 ♂/♀ [FDPR-18129], (FRPI) (DABD of FRPI); Villano, [1°30'S,77°29'W], 450 m, (Naranjo, J.), 10 Jul 1996, 1 ♂/♀ [FDPR-18140], (FRPI) (DABD of FRPI); *Sucumbíos*: Baeza-Lago Agrio rd., Cascada de San Rafael, [0°5'48"S,77°34'54"W], 1150 m, (Oña, P.), 17 Apr 2000, 1 ♂/♀ [FDPR-18161], 1 ♂/♀ [FDPR-18162], (FRPI) (DABD of FRPI); Baeza-Lago Agrio rd., Cascada de San Rafael, [0°5'48"S,77°34'54"W], 1200 m, (Willmott, K. R., Hall, J. P. W.), 24 Aug 1999, (W&H); Cerro Lumbaqui Norte, [0°1'42"N,77°19'W], 800-950 m, (Willmott, K. R., Hall, J. P. W.), 24, 26, 27 Feb 2001, (W&H), Aug, (W&H); Cerro Lumbaqui Sur, [0°0'18"N,77°19'24"W], 1050 m, (Willmott, K. R., Hall, J. P. W.), 26 Feb 2001, (W&H); km 10.5 Lumbaqui–Baeza rd., [0°0'15"S,77°25'W], 700–850 m, (Willmott, K. R., Hall, J. P. W.), 22 Feb 2001, (W&H); km 15 Lumbaqui-Pto. Libre rd., La Amarilla, [0°5'38"N,77°22'55"W], 600 m, (Willmott, K. R., Hall, J. P. W.), 27 Feb 2001, (W&H); km 7 Lumbaqui–Baeza rd., [0°1'48"N,77°22'41"W], 650 m, (Willmott, K. R., Hall, J. P. W.), 23 Feb 2001, (W&H); *Zamora-Chinchipe*: c. 3 km W Guayguayme Alto, ridge above San Luís, [3°55'14"S,78°54'49"W], 1470 m, (Willmott, K. R., J. I. R., J. C. R.), 23 Jun 2013, (W&H); km 13 Los Encuentros-Zarza, [3°48'33"S,78°36'20"W], (Willmott, K. R., Hall, J. P. W.), 8 Aug 2009, (W&H); km 18 Zumba–Los Sungas rd., Quebrada Huanchunangui, [4°55'11"S,79°9'54"W], 1100 m, (Willmott, K. R.), 11 Oct 2010, 1 ♂/♀, (W&H); Loma El Cuello, Zamora, [4°4'5"S,78°56'23"W], 1210 m, (Willmott, K. R., Hall, J. P. W.), 8 May 2008, (W&H); nr. Zamora, Río Bombuscaro, [4°6'48"S,78°57'54"W], 1146–1200 m, (Hooley, T. A.), Nov 1994, 1 ♂/♀ [MECN-TABDP-18792], 1 ♂/♀ [MECN-TABDP-18793], 1 ♂/♀ [MECN-TABDP-18797], (INABIO) (DABD of INABIO); P. N. Podocarpus, Higuerones, [4°6'S,78°57'W], 1146-1200 m, (Hernando, R.), 6 May 1998, 1 ♂/♀ [MECN-TABDP-18790], 1 ♂/♀ [MECN-TABDP-18794], (INABIO) (DABD of INABIO); ridge to west of Romerillos, [4°13'17"S,78°56'45"W], 1146–1600 m, (Hooley, T. A.), Nov 1994, 1 ♂/♀ [MECN-TABDP-18795], 1 ♂/♀ [MECN-TABDP-18796], (INABIO) (DABD of INABIO); Río Nangaritza, Shaime, [4°20'S,78°40'W], 1146-1000 m, (Hooley, T. A.), Nov 1994, 1 ♂/♀ [MECN-TABDP-18791], (INABIO) (DABD of INABIO); Río Palanda, km 55 Zumba–Loja rd., [4°37'53"S,79°8'16"W], 1100 m, (Willmott, K. R., Hall, J. P. W.), 20 Jul 1993, (W&H); Zamora, ridge to west, [4°4'30"S,78°58'7"W], 1400–1450 m, (Willmott, K. R.), 18, 20 May 2000, (W&H); Zamora-Yantzaza rd., Namírez Bajo, [3°58'6"S,78°49'48"W], 1050 m, (Willmott, K. R., Hall, J. P. W.), 21 May 2000, (W&H); *Not located*: 'Limón', (Piñas, F.), 26 Jul 1995, 1 ♂/♀ [FDPR-16646], (FRPI). **Peru**: *Ucayali*: Pucallpa, [8°23'S,74°37'W], 150 m, (Schunke, J. M.), 4 Dec 1974, 1 ♂ [MUSM-ENT-1206], (MUSM) (Darwin Butterfly Database of MUSM).

***
Amiga arnaca indianacristoi
***

**Specimens examined (16** ♂, **4** ♀) : **Venezuela**: *Aragua*: Rancho Grande Biological Station, [10°20'58"N,67°41'3"W], 1100 m, (Heppner, J. B.), Jul 1981, 1 ♀ [FLMNH-MGCL-1036240], (FLMNH); km 22 Maracay–Ocumaré rd., Rancho Grande, 1100–1200 m, (Lichy, R.), 2 Aug 1943, 1 ♂ [FLMNH-MGCL-262940], (FLMNH); Maracay, Pozo del Diablo, [10°17'N,67°37'W], 420–440 m, (Miller, L. D.), 22 Jul 1981, 1 ♂ [FLMNH-MGCL-264654], 1 ♂ [FLMNH-MGCL-264656], (FLMNH), 27 Jul 1981, 1 ♂ [FLMNH-MGCL-264655], (FLMNH); Maracay–Ocumare hwy., 1100–1200 m, (Lichy, R.), 4 Sept 1942, 1 ♀ [FLMNH-MGCL-1036235], (FLMNH); *Carabobo*: nr. Puerto Cabello, San Esteban, [10°26'N,68°1'W], (Hahnel de Sagan), 1^er^ trimestre 1877, 1 ♀ [BMNH(E)#1420075], (NHMUK), Jun, Jul 1877, 1 ♂ [BMNH(E)#1420068], 1 ♂ [BMNH(E)#1420069], (NHMUK); W of Las Trincheras, moist forest, Hacienda María Teresa, 400 m, (Miller, L. D.), 28 Jul 1981, 1 ♂ [FLMNH-MGCL-264660], (FLMNH); Yuma, [10°6'N,67°42'W], 550 m, (Miller, L. D.), 16 Dec 1981, 1 ♂ [FLMNH-MGCL-1036241], 1 ♀ [FLMNH-MGCL-1036239], (FLMNH); *Cojedes*: El Baul, Hato Pinero, (Brenner, J.), Feb 1968, 1 ♂ [FLMNH-MGCL-1036242], (FLMNH); *Distrito Federal*: Caracas, [10°30'N,66°55'W], 1200 m, (Forster, W.), 29 Jun 1954, 1 ♂, (ZSM); *Miranda*: 10 miles S of Caracas, [10°23'46"N,66°53'26"W], 1311 m, 30 Mar 1970, 1 ♂ [woods], (USNM); [Caracas], Macizo Naiguatá, 720-800 m, (Lichy, R.), 6 Dec 1942, 1 ♂ [FLMNH-MGCL-262941], (FLMNH); Massif du Naiguatá, (Lichy, R.), 5 Jul 1945, 1 ♂ [FLMNH-MGCL-264683], (FLMNH); mist forest 20 km SE Caracas, [10°21'24"N,66°46'18"W], 500-750 m, (Miller, L. D. & J. Y., Dukes, D.), 1 Sep 1990, 1 ♂ [FLMNH-MGCL-264657], 1 ♂ [FLMNH-MGCL-264659], (FLMNH); Río Chacaito, [10°25'N,66°55'W], 980–1080 m, (Lichy, R.), 12 Sep 1936, 1 ♂ [FLMNH-MGCL-264658], (FLMNH).

***
Amiga arnaca adela
***

**Specimens examined (244** ♂, **104** ♀) : **Colombia**: *Antioquia*: Cerca 13, (Marín, M. A.), 18 Sep 2011, 1 ♂, (ZUEC); Helechal Puerto Berrio, Fazenda Berlin, [6°31'N,74°26'29"W], 115 m, (Marín, M. A.), 10 Jan 2008, 1 ♂, (ZUEC); Valdivia, [7°9'N,75°27'W], (Pratt), Apr–Jun 1921, 1 ♂ [BMNH(E)#1420039], 1 ♂ [BMNH(E)#1420040], (NHMUK); *Boyacá*: Muzo, [5°32'N,74°6'W], 400-800 m, 1 ♂, (ZSM); *Chocó*: Acandi, Playa Aguacate, (Marín, M. A.), 5 Feb 2012, 1 ♂, (ZUEC), 8 Feb 2011, 1 ♂, (ZUEC); Condoto, [5°5'48"N,76°39'1"W], 46 m, (Palmer, M. G.), 1 ♀ [BMNH(E)#1420043], 1 ♀ [BMNH(E)#1420044], (NHMUK); Río San Juan, [5°13'N,76°39'W], (Trötsch), 1 ♂, 1 ♀, (MNHU); Río Tamaná, Río San Juan, Juntas, [4°59'N,76°24'W], 122 m, (Palmer, M. G.), Feb 1909, 1 ♂ [BMNH(E)#1420036], (NHMUK); *El César*: Manaure, (Simmons), 1 ♂ [BMNH(E)#1420041], (NHMUK); *Huila*: Río Hacha, [3°27'19"N,74°45'31"W], 2744 m, (Brown), Mar 1898, 1 ♂ [BMNH(E)#1498828], (NHMUK); *Meta*: San Juan, Acacias, (Trötsch), 1 ♂ [BMNH(E)#1420031], 1 ♂ [BMNH(E)#1420032], 1 ♀ [BMNH(E)#1420033], (NHMUK); *Nariño*: [Río] Yaculá, [1°29'N,78°5'W], (Hopp, W.), Jul 1921, 1 ♀, (ZSM); *Tolima*: Río Chili, [4°7'11"N,75°15'34"W], 700-800 m, 1 ♂ [BMNH(E)#1420038], (NHMUK); *Valle del Cauca*: alto Río Calima, Jan 1986, 1 ♀ [FLMNH-MGCL-257066], (FLMNH); Anchicayá, [3°34'27"N,76°46'W], 305 m, (Sullivan, J. B.), 23 Jan 1988, 1 ♂ [FLMNH-MGCL-257053], 1 ♂ [FLMNH-MGCL-257055], (FLMNH), 24 Jan 1988, 1 ♂ [FLMNH-MGCL-257051], 1 ♂ [FLMNH-MGCL-257052], 1 ♀ [FLMNH-MGCL-257058], (FLMNH); Anchicayá, [3°34'27"N,76°46'W], 600 m, (Sullivan, J. B.), 20–24 Jan 1992, 1 ♂ [FLMNH-MGCL-257056], (FLMNH); Cauca, Juntas, [3°46'27"N,76°44'41"W], (Mathan, M. de), 1897-1898, 1 ♂ [BMNH(E)#1420034], 1 ♂ [BMNH(E)#1420035], (NHMUK); Dagua, [3°40'N,76°42'W], 300 m, (Steinhauser, S. & L.), 29 Sept 1973, 1 ♂ [FLMNH-MGCL-1036217], (FLMNH); Estación Agroforestal Bajo Calima, (Sullivan, J. B.), 21 Jan 1988, 1 ♂ [FLMNH-MGCL-257057], (FLMNH); Jct of Buenaventura Old Rd and La Virgen, (Sullivan, J. B.), 3 Feb 1987, 1 ♂ [FLMNH-MGCL-257059], 1 ♀ [FLMNH-MGCL-257065], (FLMNH); Río Calima nr Tambo, Quebrada La Brea, 30 m, (Steinhauser, S. R. & L. M.), 12 Feb 1974, 1 ♀ [FLMNH-MGCL-263131], (FLMNH); Río Dagua, [3°37'N,76°40'W], 450–1600 m, (Rosenberg, W. F. H.), 1 ♂ [BMNH(E)#1498824], 1 ♂ [BMNH(E)#1498825], 1 ♂ [BMNH(E)#1498826], 1 ♂ [BMNH(E)#1498827], (NHMUK); *Not located*: Seville Island, (Batty, J. H.), 20–23 Jan 1902, 1 ♀ [BMNH(E)#1498821], 1 ♀ [BMNH(E)#1498822], (NHMUK). **Costa Rica**: *Alajuela*: 6–8 km W Atenas, [9°57'49"N,84°26'8"W], (Austin, G. T.), 22 Sep 1986, 1 ♂, (BME); Esperanza, [10°21'53"N,84°32'40"W], 200 m, Feb, 1 ♀ [BMNH(E)#1498791], (NHMUK); San Mateo, [9°57'N,84°32'W], 315–635 m, Apr, 1 ♂ [BMNH(E)#1498784], (NHMUK); *Cartago*: Turrialba, [9°53'N,83°38'W], (DZUP); rt 10, 21.8 km E Catie, (Olson, E. C.), 26 Jan 1982, 1 ♂ [FLMNH-MGCL-257046], (FLMNH); Volcán Irazú, [9°57'28"N,83°57'14"W], 1829–2134 m, (Rogers, H.), 1 ♂ [BMNH(E)#1420005], (NHMUK); *Guanacaste*: Cañas, [10°25'N,85°7'W], 137 m, (O'Shea, G.), 10 Aug 1986, 1 ♀ [FLMNH-MGCL-264667; Sta. 133 'dark part for[est]'], (FLMNH); *Heredia*: 3 km S Puerto Viejo de Sarapiquí, OTS La Selva Station, [10°25'52"N,84°0'22"W], 20–120 m, (Denno, R. F.), 23 Jun 1979, 1 ♀ [FLMNH-MGCL-1036231], 1 ♀ [FLMNH-MGCL-1036232], (FLMNH); 3.8 km N Santa Clara, (Austin, G. & A.), 3 Sep 1987, 1 ♀ [FLMNH-MGCL-257026], (FLMNH); near Chilamate, [10°26'N,84°4'W], 37 m, (Majerus, M.), 6 Jan 2005, 1 ♀ [BMNH(E)#1420012], 1 ♀ [BMNH(E)#1420030], (NHMUK); Chilamate, 10 km W Pto Viejo de Sarapiquí, Finca Selva Verde, [10°27'2"N,84°4'8"W], 37 m, (Majerus, M.), 6 Jan 2005, 1 ♂ [BMNH(E)#1420006], (NHMUK); Chilamate, W. Puerto Viejo de Sarapiquí, ruta 9, Finca Selva Verde, [10°27'2"N,84°4'8"W], 37 m, (Harris, L. & C.), 15 Feb 1993, 1 ♂ [FLMNH-MGCL-257023], 1 ♀ [FLMNH-MGCL-257025], (FLMNH); Río Peje, Magsasay, 150 m, (Hesterberg, R.), 13 Aug 1981, 1 ♂ [FLMNH-MGCL-257024], (FLMNH); Selva Verde Lodge, [10°27'2"N,84°4'8"W], 80 m, (Flaspohler), 12 Nov 1997, 1 ♀ [FLMNH-MGCL-1036238], (FLMNH); *Limón*: Guápiles, [10°13'N,83°46'W], May, 1 ♂ [BMNH(E)#1498786], (NHMUK); hill SE of Puerto Viejo, [9°38'46"N,82°44'47"W], 100 m, (Nakamura, I.), 16 Sep 2004, (ICNA); Río Estrella, [9°47'15"N,82°54'52"W], 1 ♂ [BMNH(E)#1670213], 1 ♂ [BMNH(E)#1670214], (NHMUK); *Puntarenas*: 5 km S San Vito de Java, Finca Las Cruces, [8°47'7"N,82°58'1"W], 1150 m, (Austin, G. & A.), 10 Sep 1987, 1 ♂ [FLMNH-MGCL-257029], 1 ♂ [FLMNH-MGCL-257031], (FLMNH), 29 Sep 1986, 1 ♂ [FLMNH-MGCL-257032], (FLMNH), 30 Sep 1986, 1 ♂ [FLMNH-MGCL-257027], 1 ♂ [FLMNH-MGCL-257028], 1 ♂ [FLMNH-MGCL-257030], 1 ♀ [FLMNH-MGCL-257034], 1 ♀ [FLMNH-MGCL-257035], (FLMNH), (Smith, J. A.), 29 Sep–2 Oct 1986, 6 ♂, (BME); Brujo de Buenos Aires, (Austin, G. & A.), 27 Sep 1986, 1 ♂ [FLMNH-MGCL-257038], 1 ♂ [FLMNH-MGCL-257039], 1 ♀ [FLMNH-MGCL-257041], 1 ♀ [FLMNH-MGCL-257042], 1 ♀ [FLMNH-MGCL-257043], (FLMNH); Corcovado, [8°32'N,83°35'W], (Kareofelas, G., Witham, C. W.), Aug 1983, 1 ♂, (BME); Las Alturas [Field Station], [8°57'N,82°50'W], 1500 m, (Hesterberg, R.), 15 Jun 1979, 1 ♂ [FLMNH-MGCL-257037], (FLMNH); Llorona, (DeVries, P. J.), 29 Jan 1977, 1 ♂ [FLMNH-MGCL-263073], (FLMNH); Osa Peninsula, 2.5 mi SW Rincón, [8°42'26"N,83°29'13"W], 15 m, (OTS Adv. Zoo. Course), 21–28 Feb 1967, 1 ♂ [FLMNH-MGCL-264661], (FLMNH); Osa Peninsula, Rincón, ridge N of airport, (Valerio, C. E.), 5 Aug 1970, 1 ♀ [FLMNH-MGCL-257049; primary forest], (FLMNH); Osa Peninsula, Sirena, [8°28'N,83°34'W], 110 m, (Denno, R. F.), 13 Aug 1979, 1 ♂ [FLMNH-MGCL-1036220], (FLMNH), (DeVries, P. J.), 3 Feb 1977, 1 ♂ [FLMNH-MGCL-263060], (FLMNH); Refugio Golfito, Las Torres road, 300–450 m, (Nakamura, I. & M.), 4 Sep 2004, (ICNA), (Nakamura, I.), 5 Sep 2004, (ICNA); Rincón, Península de Osa, (Fratello, S.), Feb 2000, 1 ♂ [FLMNH-MGCL-257040], (FLMNH), (Valerio, C. E.), 14 Aug 1970, 1 ♂ [FLMNH-MGCL-257044], 1 ♂ [FLMNH-MGCL-257045], 1 ♀ [FLMNH-MGCL-257048], (FLMNH); San Vito [de Java], [8°49'N,82°58'W], 1150 m, (Gard, O.), 28 Dec 1974, 1 ♀ [FLMNH-MGCL-257050], (FLMNH), (May, M. L.), 28 Jul 1970, 1 ♂ [FLMNH-MGCL-257033], (FLMNH), (Taylor, T.), 14 Jun 1971, 1 ♂ [FLMNH-MGCL-257036], (FLMNH); *San José*: Carillo, [10°9'35"N,83°57'42"W], Oct, 1 ♀ [BMNH(E)#1498792], (NHMUK); El Rodeo, University for Peace Reserve, [9°54'42"N,84°16'54"W], 1000 m, (Nakamura, I., Posla, M.), 5 Sep 2008, (ICNA); mountain 2 mi S Piedades, (Flaspohler), 3 Dec 1996, 1 ♀ [FLMNH-MGCL-1036233], (FLMNH); *Not located*: ‘Costa Rica’, 1 ♂ [BMNH(E)#1420008], 1 ♂ [BMNH(E)#1420009], 1 ♂ [BMNH(E)#1420007], 1 ♂ [BMNH(E)#1420010], 1 ♂ [BMNH(E)#1498785], 1 ♂ [BMNH(E)#1498790], 1 ♀ [BMNH(E)#1420011], (NHMUK), (Underwood), 1 ♂ [BMNH(E)#1498787], 1 ♂ [BMNH(E)#1498788], 1 ♂ [BMNH(E)#1498789], (NHMUK). (NHMUK). **Ecuador**: *Bolívar*: Balzapamba, [1°47'S,79°10'W], 17 Jun 1899, 1 ♀, (ZSM); Río Chanchan, Guayaquil–Riobamba railway, Chimbo, [2°12'S,79°8'W], 300 m, (Mathan, M. de), 1897, 1 ♂ [BMNH(E)#1420058], 1 ♂ [BMNH(E)#1420059], 305 m, (Rosenberg, W. F. H.), Aug 1897, 1 ♂ [BMNH(E)#1498844], 1 ♂ [BMNH(E)#1498845], 1 ♂ [BMNH(E)#1498846], 1 ♀ [BMNH(E)#1498847], (NHMUK); Río La Chima, tributary of Río de Las Juntas, La Chima, [1°55'35"S,79°18'24"W], 300 m, (Mathan, M. de), 1 Sep 1893, 1 ♀ [BMNH(E)#1420060], (NHMUK); *Cañar*: 'Río Angas, nr. Huigra, Angas' – (error), (Lafebre, R. de), Dec 1974, 1 ♂ [FLMNH-MGCL-263344], 1 ♂ [FLMNH-MGCL-263345], 1 ♂ [FLMNH-MGCL-263351], 1 ♂ [FLMNH-MGCL-263356], 1 ♂ [FLMNH-MGCL-263359], 1 ♀ [FLMNH-MGCL-263369], (FLMNH); *Carchi*: Lita, ridge east of Río Baboso, [0°53'15"N,78°26'18"W], 900 m, (Aldas, I.), 13 May 2015, 1 ♀ [FLMNH-MGCL-281216], (FLMNH); *Chimborazo*: Río Chanchan, Dos Puentes, [2°12'S,79°8'W], 518 m, (Coxey, W. J.), Jan 1929, 2 ♂, 1 ♀, (CMNH), Mar 1931, 3 ♂, (CMNH); *Esmeraldas*: Esmeraldas, [0°59'N,79°42'W], 0 m, (Lehmann), 1 ♂ [BMNH(E)#1420053], 1 ♀ [BMNH(E)#1420054], (NHMUK); Estación Científica Bilsa, [0°21'33"N,79°42'2"W], 600 m, (Young, A.), Jul 1999, (collection unknown) (Young, A., undergraduate research project University of Leeds); km 12.5 Lita–San Lorenzo rd., Río Chuchuví, [0°52'51"N,78°30'54"W], 750 m, (Aldas, I.), 12 May 2015, 1 ♂ [FLMNH-MGCL-281206], 1 ♂ [FLMNH-MGCL-281224], 1 ♂ [FLMNH-MGCL-281225], 1 ♂ [FLMNH-MGCL-281230], 1 ♂ [FLMNH-MGCL-281231], 1 ♂ [FLMNH-MGCL-281234], 1 ♂ [FLMNH-MGCL-281235], 1 ♂ [FLMNH-MGCL-281264], 1 ♂ [FLMNH-MGCL-281293], (FLMNH); km 12.5 Lita-San Lorenzo rd., Río Chuchuví, [0°52'51"N,78°30'54"W], 800–900 m, (Christie, J., McLoughlin, E., Ryan, F., Zakrisson, A.), 19–22 Jul 2002, (collection unknown) (Cambridge University Expedition, 2002); km 17 San Lorenzo–Ibarra rd., San Francisco ridge, [1°6'26"N,78°41'55"W], 200-250 m, (Willmott, K. R., J. I. R., J. C. R.), 6 Jul 2015, 1 ♂ [FLMNH-MGCL-209451], (FLMNH); km 40 Lita-San Lorenzo rd., El Durango, [1°2'27"N,78°38'4"W], 250–400 m, (Christie, J., McLoughlin, E., Ryan, F., Zakrisson, A.), 6–9 Aug 2002, (collection unknown) (Cambridge University Expedition, 2002); Lita-San Lorenzo railroad, Ventanas, [0°56'9"N,78°39'W], 450 m, (Christie, J., McLoughlin, E., Ryan, F., Zakrisson, A.), 14–20 Aug 2002, (collection unknown) (Cambridge University Expedition, 2002); Lita-San Lorenzo railway, Cachabé, [1°3'N,78°48'W], 150 m, (Rosenberg, W. F. H.), Jan 1897, 1 ♂ [BMNH(E)#1498922; 'low c.'], 1 ♂ [BMNH(E)#1498923; 'low c.'], 1 ♀ [BMNH(E)#1498924; 'low c.'], 1 ♀ [BMNH(E)#1498925; 'low c.'], 1 ♀ [BMNH(E)#1498926; 'low c.'], 1 ♀ [BMNH(E)#1498927; 'low c.'], (NHMUK); Lita–San Lorenzo rd., NE San Francisco, ridge N La Ceiba, [1°7'48"N,78°39'29"W], 250 m, (Aldaz, R.), Nov 2016, (INABIO); Río Santiago, Reserva de Tigrillo, lodge, [0°51'N,78°46'37"W], 100 m, (Willmott, K. R., J. I. R., J. C. R.), 11, 13 Jul 2016, 1 ♀ [FLMNH-MGCL-209996], (FLMNH), (INABIO); Río Santiago, Reserva de Tigrillo, Peñon del Santo trail, [0°51'3"N,78°46'40"W], 80 m, (Willmott, K. R., J. I. R., J. C. R.), 10, 13 Jul 2016, 1 ♂ [FLMNH-MGCL-209994], (FLMNH), (INABIO); Río Santiago, Reserva de Tigrillo, Pueblo Trail, [0°51'19"N,78°47'23"W], 100–130 m, (Aldaz, R.), 11,12 Jul 2016, (INABIO); W Maldonado–Selva Alegre rd., El Cerro, [0°58'22"N,78°55'19"W], 235 m, (Willmott, K. R., Hall, J. P. W.), 21,25 Jul 2011, 1 ♂ [FLMNH-MGCL-151127], 1 ♂ [FLMNH-MGCL-151128], (FLMNH), 4 ♂, (INABIO); *Guayas*: Guayaquil–Riobamba railway, Bucay, [2°11'S,79°6'W], 300 m, (Rhoads, S. N.), 1 ♂, (CMNH); Las Mercedes, c. 10 km S Naranjal, [2°45'56"S,79°40'11"W], 270 m, (Willmott, K. R., Hall, J. P. W.), 19 May 2008, 1 ♂ [FLMNH-MGCL-118492], (FLMNH); *Imbabura*: Río Mira, Paramba, [0°49'N,78°21'W], 1067 m, 1 ♀ [BMNH(E)#1420065], (NHMUK); Río Mira, Paramba, [0°49'N,78°21'W], 915 m, (Rosenberg, W. F. H.), Mar 1897, 1 ♂ [BMNH(E)#1498848], 1 ♂ [BMNH(E)#1498849], 1 ♂ [BMNH(E)#1498850], 1 ♂ [BMNH(E)#1498851], 1 ♀ [BMNH(E)#1498852], (NHMUK); *Manabí*: La Crespa, [0°52'S,79°52'W], 800 m, (Lafebre, R. de), May 1975, 1 ♀ [FLMNH-MGCL-257125], (FLMNH); *Pichincha*: [12 km E] Santo Domingo [de los Colorados], [Hotel] Tinalandia, [0°18'S,79°4'W], 19 Aug 1976, 1 ♂ [FLMNH-MGCL-257076], (FLMNH), 19 Aug 1976, 1 ♂ [FLMNH-MGCL-257080], 1 ♂ [FLMNH-MGCL-257081], 1 ♂ [FLMNH-MGCL-257082], (FLMNH); [12 km E] Santo Domingo [de los Colorados], [Hotel] Tinalandia, [0°18'S,79°4'W], 760 m, (Milner, P. F.), 1-5 Apr 1985, 1 ♂ [FLMNH-MGCL-257096], 1 ♂ [FLMNH-MGCL-257097], (FLMNH); [Alluriquín], Río Toachi, [0°19'6"S,78°57'13"W], 701 m, (Harris, L.& C.), 21-23 Jul 1986, 1 ♂ [FLMNH-MGCL-295865], (FLMNH); [Alluriquín], Río Toachi, [0°19'6"S,78°57'13"W], 800 m, (Brown, F. M.), Nov 1939, 1 ♂ [FLMNH-MGCL-263322], (FLMNH); 12 km E Santo Domingo de los Colorados, [Hotel] Tinalandia, [0°18'S,79°4'W], (DZUP), 5-12 May 1990, 1 ♂, (BME), (Harris, L. & C.), 25 Apr–1 May 1988, 1 ♀ [FLMNH-MGCL-264676], 1 ♀ [FLMNH-MGCL-264677], 1 ♀ [FLMNH-MGCL-264679], 1 ♀ [FLMNH-MGCL-264680], (FLMNH), 25 Apr-1 May 1988, 1 ♂ [FLMNH-MGCL-264669], (FLMNH), (Harris, L.), 25 Apr–1 May 1988, 1 ♂ [FLMNH-MGCL-1036228], (FLMNH), (Smith, J. A.), 6-11 May 1990, 4 ♂, (BME), 8–14 May 1996, 1 ♂, (BME); 12 km E Santo Domingo de los Colorados, [Hotel] Tinalandia, [0°18'S,79°4'W], 650 m, (Sullivan, J. B.), 1 Dec 1975, 1 ♀ [FLMNH-MGCL-257079], (FLMNH); 12 km E Santo Domingo de los Colorados, [Hotel] Tinalandia, [0°18'S,79°4'W], 671 m, (Emmel, T. C.), 12 May 1985, 1 ♂ [FLMNH-MGCL-257070], 1 ♂ [FLMNH-MGCL-257071], 1 ♂ [FLMNH-MGCL-257072], (FLMNH), (Nation, J. L.), 12 May 1985, 1 ♀ [FLMNH-MGCL-257077], (FLMNH); 12 km E Santo Domingo de los Colorados, [Hotel] Tinalandia, [0°18'S,79°4'W], 671-762 m, (Edwards, G. B.), 11–17 May 1986, 1 ♂ [FLMNH-MGCL-1036230], (FLMNH); 12 km E Santo Domingo de los Colorados, [Hotel] Tinalandia, [0°18'S,79°4'W], 700 m, (Covell, C. V.), 19 May 1985, 1 ♂ [FLMNH-MGCL-257074], 1 ♂ [FLMNH-MGCL-257075], (FLMNH), 21 May 1985, 1 ♂ [FLMNH-MGCL-257073], (FLMNH), 24 May 1985, 1 ♀ [FLMNH-MGCL-257078], (FLMNH); 12 km E Santo Domingo de los Colorados, [Hotel] Tinalandia, [0°18'S,79°4'W], 701 m, (Harris, L. & C.), 21–25 Jul 1986, 1 ♂ [FLMNH-MGCL-257090], 1 ♂ [FLMNH-MGCL-257091], 1 ♂ [FLMNH-MGCL-257092], 1 ♀ [FLMNH-MGCL-257093], (FLMNH); 12 km E Santo Domingo de los Colorados, [Hotel] Tinalandia, [0°18'S,79°4'W], 848 m, (Douglas, M. G.), 6-11 May 1990, 1 ♂ [FLMNH-MGCL-257109], 1 ♂ [FLMNH-MGCL-257110], 1 ♀ [FLMNH-MGCL-257112], (FLMNH); 12 km E Santo Domingo de los Colorados, [Hotel] Tinalandia, [0°18'S,79°4'W], 854 m, (Emmel, T. C.), 24 Jun 1973, 1 ♀ [FLMNH-MGCL-257101], (FLMNH); 12 km E Santo Domingo de los Colorados, Hotel Tinalandia, [0°18'S,79°4'W], 750-850 m, (Austin, G. & A.), 10 May 1988, 1 ♂ [FLMNH-MGCL-257107], (FLMNH), 13 May 1988, 1 ♂ [FLMNH-MGCL-257106], (FLMNH), 14 May 1988, 1 ♂ [FLMNH-MGCL-257098], 1 ♂ [FLMNH-MGCL-257099], 1 ♂ [FLMNH-MGCL-257100], 1 ♀ [FLMNH-MGCL-257102], (FLMNH), 8 May 1988, 1 ♂ [FLMNH-MGCL-257105], 1 ♂ [FLMNH-MGCL-257108], 1 ♀ [FLMNH-MGCL-257103], 1 ♀ [FLMNH-MGCL-257104], 1 ♀ [FLMNH-MGCL-257111], (FLMNH); 16 km E Santo Domingo, [0°19'S,79°0'W], 650 m, (Hyatt, J.), 1-4 Jul 1982, 1 ♂ [FLMNH-MGCL-257094], 1 ♂ [FLMNH-MGCL-257095], (FLMNH); 56 km N Quevedo, Estación Río Palenque, [0°36'12"S,79°18'36"W], 180 m, (Sullivan, J. B.), 2 Dec 1975, 1 ♂ [FLMNH-MGCL-257088], 1 ♂ [FLMNH-MGCL-257089], (FLMNH); Hotel Tinalandia, Río Tanti, [0°20'S,79°0'30"W], 750 m, (Hall, J. P. W., Willmott, K. R.), 7 Jan 1993, 1 ♀ [FLMNH-MGCL-257117], (FLMNH); km 20 Pacto-Guayabillas rd., [0°11'36"N,78°51'30"W], 900 m, (Busby, R. C.), 20 Jan 2015, 1 ♂ [FLMNH-MGCL-195498], (FLMNH), (Willmott, K. R., Hall, J. P. W.), 7,8 Aug 2011, 1 ♀ [FLMNH-MGCL-151129], (FLMNH), 2 ♂, (INABIO); 'Las Palmas' [=La Palma], [0°18'52"S,78°55'41"W], 900 m, (DZUP); nr. La Unión del Toachi, Otongachi Reserve, [0°19'15"S,78°56'6"W], 817 m, (Majerus, M.), 22 Feb 2007, 1 ♂ [BMNH(E)#1420045], 1 ♂ [BMNH(E)#1420046], 1 ♂ [BMNH(E)#1420047], 1 ♀ [BMNH(E)#1420048], 1 ♀ [BMNH(E)#1420049], (NHMUK); Pichincha, 1750 m, (Lafebre, R. de), Jun 1975, 1 ♂ [FLMNH-MGCL-1036226], (FLMNH); Reserva Mangaloma, [0°7'15"N,78°59'37"W], 700–815 m, (Willmott, K. R., J. I. R., J. C. R.), 10 Jul 2015, 1 ♂ [FLMNH-MGCL-209452], (FLMNH), 1 ♂, (INABIO); Río Alambi, Reserva Maquipucuna, [0°5'42"N,78°38'W], 1350 m, (Smith, N. J.), 2–3 Aug 1998, 1 ♂, (BME); Río Palenque, [0°36'12"S,79°18'36"W], 200 m, (Nicolay, S. S.), 21 Sep 1975, 1 ♂ [FLMNH-MGCL-263357], (FLMNH); Río Palenque, [0°36'12"S,79°18'36"W], 400 m, 10 Aug 1976, 1 ♂ [FLMNH-MGCL-257115], (FLMNH), 10 Aug 1976, 1 ♀ [FLMNH-MGCL-257118], (FLMNH), (Dodson, T.), 1975, 1 ♂ [FLMNH-MGCL-257120], (FLMNH), 1975, 1 ♂ [FLMNH-MGCL-257119], (FLMNH); Río Pilaton, Tandapi, [0°27'S,78°46'W], 1450 m, (Harris, L. & C.), 29 Apr 1988, 1 ♂ [FLMNH-MGCL-257137], (FLMNH); Río Toachi, Alluriquín, 800 m, (Brown, F. M.), Nov 1939, 1 ♀ [FLMNH-MGCL-263377], (FLMNH); San Pablo, [0°19'S,79°6'W], 1100 m, (Lafebre, R. de), Jun 1970, 1 ♀ [FLMNH-MGCL-263375], (FLMNH); Santo Domingo de los Colorados, [0°15'S,79°9'W], 200 m, (Goodfellow, W.), Oct 1898, 1 ♂ [BMNH(E)#1498853], (NHMUK); 'Santo Domingo de los Colorados', [0°19'S,78°59'W], 600 m, 8 Aug 1976, 1 ♂ [FLMNH-MGCL-257083], 1 ♂ [FLMNH-MGCL-257084], 1 ♂ [FLMNH-MGCL-257085], 1 ♂ [FLMNH-MGCL-257086], 1 ♀ [FLMNH-MGCL-257087], (FLMNH); 'Santo Domingo de los Colorados', [0°19'S,78°59'W], 610 m, (O'Shea, G.), 25 Jun 1983, 1 ♂ [FLMNH-MGCL-264670; Sta. 84], 1 ♂ [FLMNH-MGCL-264671; Sta. 80], 1 ♀ [FLMNH-MGCL-264678; Sta. 80], (FLMNH); Santo Domingo, [0°15'S,79°9'W], 350 m, (Jenkins, D. W.), 16 Nov 1975, 1 ♂ [FLMNH-MGCL-257116], (FLMNH); Taguaza, [0°18'S,79°2'W], 534 m, (Lafebre, R. de), Jun 1975, 1 ♂ [FLMNH-MGCL-264673], 1 ♂ [FLMNH-MGCL-264674], 1 ♂ [FLMNH-MGCL-264675], 1 ♂ [FLMNH-MGCL-264681], (FLMNH), 1 ♂ [FLMNH-MGCL-264672], 1 ♂ [FLMNH-MGCL-257114], (FLMNH), Jun 1975, 1 ♂ [FLMNH-MGCL-257113], 1 ♂ [FLMNH-MGCL-263051], (FLMNH); 'Toachi', [0°19'S,78°57'W], 600 m, (Velástegui, D.), 20 Jul 1968, 1 ♂ [MUSM-LEP-100440], (MUSM); *Not located*: ‘Ecuador’, 1 ♂, (MNHU). **Nicaragua**: *Atlántico Norte*: 185 miles above C. Gracias, San Ramón, [14°40'N,84°41'W], 114 m, (Palmer, M. G.), May 1905, 1 ♂ [BMNH(E)#1498783], (NHMUK); Río Wanks, (Palmer, M. G.), 1905, 1 ♂ [BMNH(E)#1498782; Wet Seas.[on]], (NHMUK); *Atlántico Sur*: Bluefields, [12°1'N,83°46'W], (Sullivan, J. B.), 10 Dec 1975, 1 ♀ [FLMNH-MGCL-257149], (FLMNH), 11 Dec 1975, 1 ♀ [FLMNH-MGCL-257150], (FLMNH); Nueva Guinea, [11°40'N,84°27'W], (Anderson, R. A.), 26 Aug 1976, 1 ♂ [FLMNH-MGCL-263072], (FLMNH), 26 Oct 1976, 1 ♀ [FLMNH-MGCL-257143], (FLMNH), 27 Oct 1976, 1 ♂ [FLMNH-MGCL-257139], 1 ♂ [FLMNH-MGCL-257142], 1 ♂ [FLMNH-MGCL-263066], 1 ♀ [FLMNH-MGCL-257148], 1 ♀ [FLMNH-MGCL-263091], (FLMNH), 28 Aug 1976, 1 ♂ [FLMNH-MGCL-263067], (FLMNH), 28 Oct 1976, 1 ♂ [FLMNH-MGCL-257141], 1 ♀ [FLMNH-MGCL-257145], 1 ♀ [FLMNH-MGCL-257146], 1 ♀ [FLMNH-MGCL-257147], (FLMNH), 29 Oct 1976, 1 ♂ [FLMNH-MGCL-257140], 1 ♀ [FLMNH-MGCL-257144], (FLMNH); *Chontales*: 'Chontales' [=Santo Domingo], [12°15'44"N,85°4'58"W], 500 m, (Belt, T.), 1 ♂ [BMNH(E)#1420001], 1 ♀ [BMNH(E)#1420002], 1 ♀ [BMNH(E)#1420003], (NHMUK), (Janson), 1 ♂ [BMNH(E)#1420000], (NHMUK); *Not located*: ‘Nicaragua’, 1 ♀ [BMNH(E)#1420004], (NHMUK). **Panama**: *Chiriquí*: Bugaba, [8°29'N,82°37'W], 250–475 m, (Champion, G. C.), 1 ♂ [BMNH(E)#1420015], 1 ♂ [BMNH(E)#1420019], 1 ♀ [BMNH(E)#1420026], (NHMUK); Bugaba, 240 m, 1 ♀ [BMNH(E)#1420028], (NHMUK); Bugaba, 244 m, (Watson), 1 ♂ [BMNH(E)#1498816], (NHMUK), (Watson, A.), 1 ♀ [BMNH(E)#1498823], (NHMUK); Chiriquí, [8°40'34"N,82°37'26"W], 2 ♂, (MNHU), (Ribbe), 1 ♂, 1 ♀, (MNHU); Chiriquí, [8°40'34"N,82°37'26"W], 760–1220 m, (Champion, G. C.), 1 ♂ [BMNH(E)#1420013], 1 ♂ [BMNH(E)#1420014], 1 ♂ [BMNH(E)#1420018], 1 ♀ [BMNH(E)#1420025], (NHMUK); David, [8°25'N,82°27'W], (Champion, G. C.), 1 ♂ [BMNH(E)#1420022], (NHMUK); Potrerillos, [8°48'N,82°30'W], 1100–1200 m, (Tr.[ötsch]), 1 ♂, (MNHU); *Coclé*: Cerro La Vieja Eco-Hotel, [8°40'2"N,80°12'4"W], 410 m, (Klein, T. W.), 5 Aug 2008, 1 ♂ [FLMNH-MGCL-257151], (FLMNH); El Valle, [8°36'N,80°8'W], 800–950 m, (Anderson, R. A.), 1 Oct 1984, 1 ♂ [FLMNH-MGCL-263083], (FLMNH); *Colón*: Gamboa, [9°7'13"N,79°41'59"W], 50 m, (Anderson, R. A.), 29 Jul 1985, 1 ♀ [FLMNH-MGCL-263090], (FLMNH); Gatún, [9°16'N,79°55'W], 110 m, (R. J. J.), 1 Jan 1946, 1 ♀ [FLMNH-MGCL-263094], (FLMNH), 3 Sep 1945, 1 ♂ [FLMNH-MGCL-263074], (FLMNH), (R. W.), 8 Sep 1945, 1 ♀ [FLMNH-MGCL-263126], (FLMNH); Lion Hill, [9°13'40"N,79°53'40"W], 80 m, (McLeannan), 1 ♂ [BMNH(E)#1420016], 1 ♂ [BMNH(E)#1420020], (NHMUK); Madden Forest, [9°12'45"N,79°36'57"W], 80 m, (Anderson, R. A.), 29 Sep 1976, 1 ♀ [FLMNH-MGCL-263127], (FLMNH); Matachin, [9°7'1"N,79°42'14"W], 30 m, (Thieme, O.), Jul 1877, 1 ♂ [BMNH(E)#1420023], 1 ♂ [BMNH(E)#1420024], (NHMUK); Piña, [9°16'50"N,80°2'49"W], 10 m, 1 Mar 1969, 1 ♂ [FLMNH-MGCL-263062], (FLMNH), (King, H. L.), 1 Apr 1970, 1 ♀ [FLMNH-MGCL-263089], (FLMNH), 1 Jul 1970, 1 ♂ [FLMNH-MGCL-263070], (FLMNH), 11 Mar 1971, 1 ♂ [FLMNH-MGCL-263054], (FLMNH), 15 Jun 1970, 1 ♂ [FLMNH-MGCL-263061], (FLMNH), 16 Jul 1970, 1 ♂ [FLMNH-MGCL-263071], (FLMNH), 18 Jul 1970, 1 ♀ [FLMNH-MGCL-263093], (FLMNH), 18 Jun 1970, 1 ♂ [FLMNH-MGCL-263065], 1 ♂ [FLMNH-MGCL-263084], 1 ♂ [FLMNH-MGCL-263085], (FLMNH), 2 Aug 1970, 1 ♂ [FLMNH-MGCL-263075], (FLMNH), 20 Jun 1970, 1 ♂ [FLMNH-MGCL-263063], 1 ♂ [FLMNH-MGCL-263064], 1 ♂ [FLMNH-MGCL-263076], 1 ♂ [FLMNH-MGCL-263077], 1 ♂ [FLMNH-MGCL-263086], (FLMNH), 20 Jun 1972, 1 ♂ [FLMNH-MGCL-263059], (FLMNH), 22 Jul 1970, 1 ♂ [FLMNH-MGCL-263080], (FLMNH), 29 Jul 1970, 1 ♀ [FLMNH-MGCL-263096], (FLMNH), 3 Aug 1970, 1 ♂ [FLMNH-MGCL-263068], (FLMNH), 5 Aug 1970, 1 ♂ [FLMNH-MGCL-263069], 1 ♀ [FLMNH-MGCL-263087], 1 ♀ [FLMNH-MGCL-263098], (FLMNH), 6 Jun 1970, 1 ♂ [FLMNH-MGCL-263081], (FLMNH), 7 Jul 1970, 1 ♂ [FLMNH-MGCL-263078], (FLMNH), 9 Jul 1970, 1 ♀ [FLMNH-MGCL-263092], (FLMNH), (King, H.L.), 9 Jul 1970, 1 ♂ [FLMNH-MGCL-295864], (FLMNH); Piña, [9°16'50"N,80°2'49"W], 100 m, (King, H. L.), 1 May 1971, 1 ♀ [FLMNH-MGCL-263095], (FLMNH); Piña, [9°16'50"N,80°2'49"W], 200 m, (King, H. L.), 11 Jul 1972, 1 ♀ [FLMNH-MGCL-263088], (FLMNH), 13 Jul 1972, 1 ♀ [FLMNH-MGCL-263097], (FLMNH), 25 Jun 1972, 1 ♂ [FLMNH-MGCL-263058], (FLMNH), 30 Aug 1972, 1 ♂ [FLMNH-MGCL-263082], (FLMNH); *Darién*: Majé Island, Lake Bayano, 'Gorgas Bayano Sta.' [=Gorgas Memorial Laboratory], (Jenkins, D. W.), 25 Dec 1975, 1 ♀ [FLMNH-MGCL-257152], (FLMNH); Veraguas, (Arcé), 1 ♂ [BMNH(E)#1420017], 1 ♂ [BMNH(E)#1420021], 1 ♀ [BMNH(E)#1420027], (NHMUK); *Panamá*: Cerro Campana, [8°41'N,79°55'W], 750-792 m, (Anderson, R. A.), 5 Dec 1978, 1 ♂ [FLMNH-MGCL-263079], (FLMNH), (King, H. L.), 11 Jan 1973, 1 ♀ [FLMNH-MGCL-263125], (FLMNH); near Gamboa, [9°7'13"N,79°41'59"W], 30-150 m, (Majerus, M.), 22 Sep 2001, 1 ♀ [BMNH(E)#1420029], (NHMUK); Summit, [9°3'53"N,79°38'49"W], 90 m, (Robbins, R. K.), 30 Nov 1978, 1 ♀ [FLMNH-MGCL-263124], (FLMNH); *Not located*: Isthmus of Panamá, Dec 1907, 1 ♂ [BMNH(E)#1498793], 1 ♂ [BMNH(E)#1498794], 1 ♂ [BMNH(E)#1498795], 1 ♂ [BMNH(E)#1498796], 1 ♂ [BMNH(E)#1498797], (NHMUK); ‘Panama’, 1 ♂ [BMNH(E)#1498798], (NHMUK).

**Other records**: **Colombia**: *Antioquia*: Caracoli, El Cascarón-Finca Pataratas, 1000 m, (Henao, E.), 2 Oct 2004, 1 ♂/♀ [MEFLG-15503; jama], 1 ♂/♀ [MEFLG-8488; jama], (MEFLG); Puerto Berrio, Berlin, [6°31'N,74°26'29"W], 115 m, (Alvarez, C. F.), 11 Jan 2008, 1 ♂/♀ [MEFLG-15507; jama, borde bosque], 1 ♂/♀ [MEFLG-15511; jama, borde bosque], 1 ♂/♀ [MEFLG-15516; jama, borde bosque], (MEFLG), 13 Jan 2008, 1 ♂/♀ [MEFLG-15517; jama, borde bosque], (MEFLG), 14 Jan 2008, 1 ♂/♀ [MEFLG-15508; jama], 1 ♂/♀ [MEFLG-15510; jama, borde bosque], (MEFLG), (Marín, M. A.), 11 Jan 2008, 1 ♂/♀ [MEFLG-15509; jama, interior bosque 150 m], (MEFLG), 13 Jan 2008, 1 ♂/♀ [MEFLG-15515; jama, interior bosque 100 m], (MEFLG), 14 Jan 2008, 1 ♂/♀ [MEFLG-15513; trampa-fruta, interior bosque 100 m], (MEFLG); San Roque, San José del Nus, [6°28'42"N,74°51'9"W], 1000 m, (Marín, M. A.), 29-Nov-08, 1 ♂/♀ [MEFLG-15504; jama], 1 ♂/♀ [MEFLG-15505; jama], 1 ♂/♀ [MEFLG-15506; jama], 1 ♂/♀ [MEFLG-15512; jama], 1 ♂/♀ [MEFLG-15514; jama], (MEFLG). **Ecuador**: *Carchi*: Lita, ridge east of Río Baboso, [0°53'15"N,78°26'18"W], 1000 m, (Willmott, K. R., Hall, J. P. W.), Jul, (W&H); *Esmeraldas*: Reserva Río Canandé, [0°29'N,79°12'4"W], 400 m, (Levy, E.), 2011, (QCAZ) (Checa, F., pers. comm., by email 26 Jun 2012); [km 40 Lita-San Lorenzo rd.], 'Durango' [=El Durango], [1°2'27"N,78°38'4"W], 500 m, (Piñas, F.), 20 Jul 2003, 1 ♂/♀ [FDPR-18157], (FRPI) (DABD of FRPI); c. km 5 San Mateo–Pto. Libre rd., Cerro Mutiles, [0°54'N,79°36'W], 100 m, (Willmott, K. R.), 9 May 2000, (W&H); hill at jct. rd. to Ricaurte, km 17 San Lorenzo–Lita rd., [1°10'25"N,78°44'45"W], 100 m, (Willmott, K. R., Hall, J. P. W.), 2, 4 Dec 1996, (W&H); km 10 San Lorenzo–Lita rd., Estación Experimental 'La Chiquita', [1°13'49"N,78°45'57"W], 50 m, (Willmott, K. R., Hall, J. P. W.), 2,3 Dec 1996, (W&H), 3 Mar 2001, (W&H), 7 May 2000, (W&H); km 12.5 Lita–San Lorenzo rd., Río Chuchuví, [0°52'51"N,78°30'54"W], 800–900 m, (Willmott, K. R., Hall, J. P. W.), 15,16,18 Jul 1999, (W&H); km 15 Lita–San Lorenzo rd., [0°53'52"N,78°31'29"W], 800 m, (Willmott, K. R., Hall, J. P. W.), 6 May 2000, (W&H); km 16 Lita–San Lorenzo rd., Anchayacu, [0°53'13"N,78°31'49"W], 950–1000 m, (Willmott, K. R.), 1 Jul 1998, (W&H); km 17 Lita–San Lorenzo rd., El Encanto, [0°53'35"N,78°32'13"W], 850–-900 m, (Willmott, K. R., Hall, J. P. W.), Jul, (W&H); km 18.5 San Mateo–Pto. Libre rd., Zapatta, [0°53'6"N,79°32'25"W], 500 m, (Willmott, K. R., Hall, J. P. W.), 5,6 Mar 2001, (W&H); km 24 Lita–San Lorenzo rd., Finca Durán, [0°56'20"N,78°33'30"W], 600 m, (Willmott, K. R.), 22, 24 Jul 1999, (W&H); km 40 Lita–San Lorenzo rd., El Durango, [1°2'27"N,78°38'4"W], 300-400 m, (Willmott, K. R., Hall, J. P. W.), 8 May 2000, (W&H), Aug, (W&H), Jul, (W&H), Mar, (W&H); km 44 Lita–San Lorenzo rd., La Punta, [1°3'55"N,78°39'W], 300 m, (Willmott, K. R., Hall, J. P. W.), 20,21,25 Aug 1996, (W&H); km 6 San Mateo–Pto. Libre rd., [0°53'13"N,79°35'16"W], 350 m, (Willmott, K. R., Hall, J. P. W.), 6 Mar 2001, (W&H); Reserva Canandé Lodge, [0°31'16"N,79°12'30"W], 420 m, (Willmott, K. R., Hall, J. P. W.), 30 Jul 2011, (W&H); Río Cachaví, km 20 Lita–San Lorenzo rd., 1 km W Alto Tambo, [0°54'44"N,78°32'50"W], 700-750 m, (Hall, J. P. W.), 2 Mar 2001, (W&H); Río San Miguel, San Miguel, [0°45'N,78°55'48"W], 100 m, (Willmott, K. R., Hall, J. P. W.), 9–11 Jun 1994, (W&H); San Lorenzo, [1°2'27"N,78°38'4"W], 300 m, (Piñas, F.), 19 Jul 2003, 1 ♂/♀ [FDPR-18191], (FRPI) (DABD of FRPI); San Lorenzo-Lita rd., Tundaloma Lodge, [1°10'40"N,78°44'54"W], 100 m, (Willmott, K. R., Hall, J. P. W.), 17–19 Jul 2011, (W&H); *Imbabura*: 37 km N Pedro Vicente Maldonado, Rumiñahui, [0°16'44"N,78°59'54"W], 500 m, (Willmott, K. R., Hall, J. P. W.), 9 Mar 2001, (W&H); c. 6 km E Lita, Cachaco, ridge to south, [0°48'47"N,78°25'W], 1300 m, (Willmott, K. R.), 16,23 Jul 1999, (W&H); S of Santa Rita de Cachaco, S bank Río Verde, [0°44'38"N,78°23'W], 1450 m, (Willmott, K. R.), 3 Aug 1999, (W&H); *Pichincha*: [Pedro Vicente] Maldonado, [0°4'53"N,79°2'57"W], 1100 m, (Piñas, F.), 13 Jul 2001, 1 ♂/♀ [FDPR-16680], (FRPI) (DABD of FRPI); [Pedro Vicente] Maldonado, [0°4'53"N,79°2'57"W], 710 m, (Piñas, F.), 15 May 2004, 1 ♂/♀ [FDPR-16651], (FRPI) (DABD of FRPI); 12 km E Santo Domingo de los Colorados, [Hotel] Tinalandia, [0°18'S,79°4'W], 800 m, (Covell, C. C.), 28 May 1996, 1 ♂/♀ [FDPR-18207], (FRPI), (Gil, J.), 14 Feb 2000, 1 ♂/♀ [FDPR-18179], (FRPI), (Piñas, F.), 1 Nov 1999, 1 ♂/♀ [FDPR-16633], (FRPI), 11 Aug 2000, 1 ♂/♀ [FDPR-18189], 1 ♂/♀ [FDPR-18212], (FRPI), 13 Dec 2002, 1 ♂/♀ [FDPR-16634], 1 ♂/♀ [FDPR-16636], 1 ♂/♀ [FDPR-16638], 1 ♂/♀ [FDPR-18149], 1 ♂/♀ [FDPR-18172], 1 ♂/♀ [FDPR-18174], (FRPI), 14 Feb 2000, 1 ♂/♀ [FDPR-18176], (FRPI), 15 Jun 1996, 1 ♂/♀ [FDPR-16640], 1 ♂/♀ [FDPR-18148], 1 ♂/♀ [FDPR-18150], 1 ♂/♀ [FDPR-18151], 1 ♂/♀ [FDPR-18152], 1 ♂/♀ [FDPR-18168], 1 ♂/♀ [FDPR-18201], 1 ♂/♀ [FDPR-18202], 1 ♂/♀ [FDPR-18203], 1 ♂/♀ [FDPR-18208], (FRPI), 18 Sep 1999, 1 ♂/♀ [FDPR-16683], (FRPI), 20 Mar 2001, 1 ♂/♀ [FDPR-18159], 1 ♂/♀ [FDPR-18200], (FRPI), 21 Aug 1999, 1 ♂/♀ [FDPR-18169], (FRPI), 25 Jul 2000, 1 ♂/♀ [FDPR-18164], (FRPI), 6 Sep 2000, 1 ♂/♀ [FDPR-16632], 1 ♂/♀ [FDPR-18155], 1 ♂/♀ [FDPR-18163], (FRPI), 6 Sep 2002, 1 ♂/♀ [FDPR-16642], (FRPI), 7 Oct 2001, 1 ♂/♀ [FDPR-16689], 1 ♂/♀ [FDPR-16695], 1 ♂/♀ [FDPR-18144], (FRPI), 8 Sep 2001, 1 ♂/♀ [FDPR-18130], 1 ♂/♀ [FDPR-18137], (FRPI) (DABD of FRPI); 12 km E Santo Domingo de los Colorados, [Hotel] Tinalandia, [0°18'S,79°4'W], 80–800 m, (Piñas, F.), 15 Jun 1996, 1 ♂/♀ [FDPR-18192], (FRPI) (DABD of FRPI); Hotel Tinalandia, Río Tanti, [0°20'S,79°0'30"W], 750-800 m, (Willmott, K. R., Hall, J. P. W.), 29 Jun 1993, (W&H), 8-14 May 1994, (W&H); La Esperie, [0°20'S,78°58'W], 1205-1250 m, (Piñas, F.), 13 Jun 2001, 1 ♂/♀ [FDPR-16635], (FRPI) (DABD of FRPI); La Esperie, [0°20'S,78°58'W], 1250 m, (Piñas, F.), 14 Oct 1999, 1 ♂/♀ [FDPR-18181], (FRPI), 30 Jun 2001, 1 ♂/♀ [FDPR-18153], 1 ♂/♀ [FDPR-18154], (FRPI), 4 Sep 1999, 1 ♂/♀ [FDPR-16639], (FRPI), 7 Feb 1998, 1 ♂/♀ [FDPR-16693], 1 ♂/♀ [FDPR-16706], 1 ♂/♀ [FDPR-16707], 1 ♂/♀ [FDPR-18213], (FRPI) (DABD of FRPI); Río Pilaton, Tandapi, [0°27'S,78°46'W], 1550 m, (Willmott, K. R., Hall, J. P. W.), Aug, (W&H); Río Toachi, Alluriquín, [0°19'S,78°59'45"W], 900 m, (Piñas, F.), 2 Sep 1999, 1 ♂/♀ [FDPR-18170], 1 ♂/♀ [FDPR-18206], (FRPI), 21 Sep 2000, 1 ♂/♀ [FDPR-18186], (FRPI), 29 Jul 2000, 1 ♂/♀ [FDPR-16686], (FRPI), Sep 1998, 1 ♂/♀ [FDPR-16678], (FRPI) (DABD of FRPI); San Miguel de Los Bancos, [0°1'24"N,78°53'45"W], 1000 m, (Piñas, F.), 17 Nov 1995, 1 ♂/♀ [FDPR-16687], (FRPI) (DABD of FRPI); 'Toachi', [0°19'S,78°57'W], 1100 m, (Piñas, F.), 1 Mar 2000, 1 ♂/♀ [FDPR-16701], (FRPI), 7 Oct 1995, 1 ♂/♀ [FDPR-16645], (FRPI) (DABD of FRPI); 'Toachi', [0°19'S,78°57'W], 800 m, (Piñas, F.), 7 Oct 1997, 1 ♂/♀ [FDPR-16692], (FRPI) (DABD of FRPI).

***
Amiga arnaca sericeella
***

**Specimens examined (46** ♂, **23** ♀) : **Guatemala**: *Alta Verapaz*: ‘forests of N Vera Paz’, (Bates, H. W.), 1 ♀ [HT *sericeella*: 'Godman-Salvin Coll. 1904-1. B.C.A. Lep.Rhop. Euptychiasericella, Bates.'//'B.M. TYPE No. Rh3131 Euptychiasericella, ♀ Bates.'//'Type. Sp. Figured.'//'Forests of N. Vara Paz F.D.G. & O.S.'//'Type H.T.'//'♀'], (NHMUK), (F. D. G. & O. S.), 1 ♀ [BMNH(E)#1267030], (NHMUK); Choctún, [15°40'N,90°25'W], (Godman, F. D., Salvin, O.), 1 ♀ [BMNH(E)#1420483], (NHMUK). **Honduras**: *Copán*: Copán Ruínas, [15°8'N,88°18'W], 1320 m, Mar 2006, 1 ♂ [FLMNH-MGCL-264632], (FLMNH), Mar 2006, (FLMNH). **Mexico**: *Chiapas*: Bombaná, (Wind, R.), 9-11 Oct 1973, 1 ♀ [FLMNH-MGCL-1036244], 1 ♀ [FLMNH-MGCL-1036246], (FLMNH); Campet, [16°58'N,93°15'W], (Wind, R.), 1969, 1 ♂ [FLMNH-MGCL-207911], (FLMNH); mountains above Escuintla, 610 m, (Wind, R.), 8 Sep 1974, 1 ♀ [FLMNH-MGCL-207912], (FLMNH); Mt. above San Quintín, (Wind, R.), 20–26 Nov 1973, 1 ♀ [FLMNH-MGCL-264079], (FLMNH), 26 Feb 1973, 1 ♂ [FLMNH-MGCL-207899], 1 ♂ [FLMNH-MGCL-207900], 1 ♂ [FLMNH-MGCL-207901], 1 ♂ [FLMNH-MGCL-207902], 1 ♂ [FLMNH-MGCL-207903], 1 ♂ [FLMNH-MGCL-207904], 1 ♂ [FLMNH-MGCL-207908], 1 ♂ [FLMNH-MGCL-207909], 1 ♀ [FLMNH-MGCL-207905], 1 ♀ [FLMNH-MGCL-207906], 1 ♀ [FLMNH-MGCL-207907], (FLMNH); Palestina, [16°30'43"N,91°45'56"W], 1400 m, 1 ♂ [FLMNH-MGCL-207910], (FLMNH); Pichucalco, [17°31'N,93°9'W], (Escalante, T.), 8 Sep 1974, 1 ♀ [FLMNH-MGCL-207913], (FLMNH); San Quintín, [16°24'N,91°20'W], (Wind, R.), 3 May 1973, 1 ♂ [FLMNH-MGCL-1036243], (FLMNH); Tapilula, [17°14'N,93°2'W], (Wind, R.), 14-15 Nov 1973, 1 ♀ [FLMNH-MGCL-264080; 30], (FLMNH); *Oaxaca*: Mpio. Comaltepec, Vista Hermosa, [17°41'N,96°25'W], 1000 m, (Díaz Francés, A.), Jul 1964, 1 ♂ [FLMNH-MGCL-207884], 1 ♂ [FLMNH-MGCL-207885], 1 ♂ [FLMNH-MGCL-207886], 1 ♂ [FLMNH-MGCL-207887], (FLMNH); Soyolapan el Bajo, [17°44'N,96°21'W], 183 m, (Ross, G. N.), 26 Aug 1961, 1 ♀ [FLMNH-MGCL-264077], (FLMNH), 9 Aug 1961, 1 ♀ [FLMNH-MGCL-264078], (FLMNH); Totontepec, [17°13'N,96°3'W], Jun 1945, 1 ♂ [FLMNH-MGCL-207895], (FLMNH); Tuxtepec, [18°6'N,96°7'W], (Díaz Francés, A.), Aug 1967, 1 ♂ [FLMNH-MGCL-207888], 1 ♂ [FLMNH-MGCL-207889], 1 ♂ [FLMNH-MGCL-207890], (FLMNH); *Veracruz*: Catemaco 8 mi SSE, 549 m, (Ross, G.N.), 16 Oct 1962, 1 ♂ [FLMNH-MGCL-295866; 3187], (FLMNH); Catemaco, [18°25'N,95°7'W], Jun 1959, 1 ♂ [FLMNH-MGCL-207894], (FLMNH), (Escalante, T.), Sept 1965, 1 ♀ [FLMNH-MGCL-1036247], (FLMNH); Los Tuxtlas, [18°27'N,95°10'W], 500 m, (Díaz Francés, A.), Jul 1971, 1 ♀ [FLMNH-MGCL-207898], (FLMNH); Presidio, [18°41'N,96°45'W], Sep 1914, 1 ♂, (ZSM), (Escalante, T.), Jul 1950, 1 ♀ [FLMNH-MGCL-207896], (FLMNH), Jun 1947, 1 ♂ [FLMNH-MGCL-207893], (FLMNH), Jun 1951, 1 ♀ [FLMNH-MGCL-207897], (FLMNH), Jun 1955, 1 ♂ [FLMNH-MGCL-207891], (FLMNH), Jun 1956, 1 ♂ [FLMNH-MGCL-207892], (FLMNH), (Lau, A. B.), 15 Jun 1966, 1 ♂, (BME); Presidio, [18°41'N,96°45'W], 900 m, (Welling, E. C.), 11 Jul 1975, 1 ♂ [FLMNH-MGCL-207883], (FLMNH); Veracruz, [19°12'N,96°8'W], (Ross, G. N.), 13 Apr 1965, 1 ♂ [FLMNH-MGCL-264085; 5126], (FLMNH), 16 Oct 1962, 1 ♂ [FLMNH-MGCL-264066; 3189], 1 ♂ [FLMNH-MGCL-264082; 3188], 1 ♂ [FLMNH-MGCL-264083; 3190], (FLMNH), 18 Oct 1962, 1 ♂ [FLMNH-MGCL-264073; 3221], 1 ♂ [FLMNH-MGCL-264074; 3222], 1 ♂ [FLMNH-MGCL-264081; 3223], (FLMNH), 21 Jun 1963, 1 ♂ [FLMNH-MGCL-264084; 4040], (FLMNH), 24 Oct 1962, 1 ♂ [FLMNH-MGCL-264086; 3376], (FLMNH), 24 Sep 1962, 1 ♂ [FLMNH-MGCL-264071; 2955], 1 ♂ [FLMNH-MGCL-264072; 2956], (FLMNH), 27 Sep 1962, 1 ♂ [FLMNH-MGCL-264067; 3026], (FLMNH), 29 Sep 1962, 1 ♂ [FLMNH-MGCL-264068; 3024], 1 ♂ [FLMNH-MGCL-264069; 3025], 1 ♂ [FLMNH-MGCL-264070; 3027], 1 ♀ [FLMNH-MGCL-264075; 3028], 1 ♀ [FLMNH-MGCL-264076; 3029], (FLMNH); *Not located*: ‘Mexico’, 1 ♂, (ZSM). **Country unknown**: *Not located*: no data, 1 ♂ [BMNH(E)#1420484], (NHMUK).

***Amigaarnaca*, subspecies not recorded at time of data entry**

**Specimens examined (35** ♂, **6** ♀) : **Honduras**: *Not located*: ‘Honduras’ – (error), 1 ♂ [BMNH(E)#1498781], 1 ♀ [BMNH(E)#1498951], (NHMUK). **Colombia**: *Cundinamarca*: ‘Bogotá’, [4°35'N,74°4'W], 2600-2900 m, 1 ♂ [BMNH(E)#1420037; 'Reçu du Frère Apollinaire-Marie de Bogotá'], 1 ♂ [BMNH(E)#1498799], 1 ♂ [BMNH(E)#1498800], 1 ♂ [BMNH(E)#1498801], 1 ♂ [BMNH(E)#1498802], 1 ♂ [BMNH(E)#1498803], 1 ♂ [BMNH(E)#1498804], 1 ♂ [BMNH(E)#1498805], 1 ♂ [BMNH(E)#1498806], 1 ♂ [BMNH(E)#1498807], 1 ♂ [BMNH(E)#1498808], 1 ♂ [BMNH(E)#1498809], 1 ♂ [BMNH(E)#1498810], 1 ♂ [BMNH(E)#1498811], 1 ♂ [BMNH(E)#1498812], 1 ♂ [BMNH(E)#1498813], 1 ♂ [BMNH(E)#1498814], 1 ♂ [BMNH(E)#1498815], 1 ♂ [BMNH(E)#1498818], 1 ♂ [BMNH(E)#1498819], 1 ♂ [BMNH(E)#1498820], (NHMUK); *Not located*: ‘Colombia’, 1 ♂ [BMNH(E)#1420042], 1 ♂ [BMNH(E)#1498839], 1 ♂ [BMNH(E)#1498966], 1 ♀ [BMNH(E)#1498817], (NHMUK). **Ecuador**: *Not located*: ‘Ecuador’, 1 ♂ [BMNH(E)#1420062], 1 ♂ [BMNH(E)#1420063], 1 ♂ [BMNH(E)#1420064], 1 ♀ [BMNH(E)#1420194], (NHMUK). **Venezuela**: *Not located*: ‘Venezuela’, 1 ♂ [BMNH(E)#1420071], 1 ♂ [BMNH(E)#1420072], 1 ♂ [BMNH(E)#1498902], 1 ♀ [BMNH(E)#1420073], 1 ♀ [BMNH(E)#1420074], (NHMUK), (Dyson), 1 ♂ [BMNH(E)#1420069], 1 ♂ [BMNH(E)#1420070], (NHMUK). **Country unknown**: *Not located*: no data, 1 ♂ [FLMNH-MGCL-263105], 1 ♂ [FLMNH-MGCL-263123], (FLMNH).

## Supplementary materials

Supplementary material 1 Results of maximum likelihood analysis based on each individual dataset of anchored hybrid enrichment and four genes (COI, EF1a, RPS5, GAPDH) Data type: statistical data Explanation note: Support values indicate ultrafast bootstrap. This is an open access article distributed under the terms of the Creative Commons Attribution License (CC BY 4.0), which permits unrestricted use, distribution, and reproduction in any medium, provided the original author and source are credited.Shinichi Nakahara, Gerardo Lamas, Stephanie Tyler, Mario Alejandro Marín, Blanca Huertas, Keith R. Willmott, Olaf H. H. Mielke, Marianne Espeland

## References

[B1] Barcant M (1970) Butterflies of Trinidad and Tobago. Collins, London, 314 pp.

[B2] Bates H (1864–1865) New species of butterflies from Guatemala and Panama, collected by Osbert Salvin and F. du Cane Godman, Esqs. Entomologist's Monthly Magazine 1(1): 1–6 (June 1864), (2): 31–35 (July 1864), (3): 55–59 (August 1864), (4): 81–85 (September 1864), (5): 113–116 (October 1864), (6): 126–-131 (November 1864), (7): 161–164 (December 1864), (8): 178–180 (January 1865), (9): 202–205 (February 1865).

[B3] Beccaloni GW Viloria AL Hall SK Robinson GS (2008) Catalogue of the hostplants of the Neotropical butterflies. Catálogo de las plantas huésped de las mariposas neotropicales. Monografías del Tercer Milenio, Zaragoza, Sociedad Entomológica Aragonesa 8: 1–536.

[B4] Benmesbah M Zacca T Casagrande MM Mielke OHH Lamas G Willmott KR (2018) Taxonomic notes on *Papilioocypete* Fabricius, 1776 and *Papiliohelle* Cramer, 1779 with description of two new similar species from South America (Lepidoptera: Nymphalidae: Satyrinae). Zootaxa 4425(1): 115–145. 10.11646/zootaxa.4425.1.7 30313470

[B5] Brévignon C (2008) Inventaire des Satyrinae de Guyane française (Lepidoptera : Nymphalidae). In : Lacomme D, Manil L (Eds) Lépidoptères de Guyane. Tome 3. Rhopalocères 2. Association des Lépidoptéristes de France, Paris, 62–94.

[B6] Brévignon C Benmesbah M (2012) Complément à l’inventaire des Satyrinae de Guyane (Lepidoptera: Nymphalidae). In: Lacomme D Manil L (Eds) Lépidoptères de Guyane, Tome 7, Nymphalidae.Lepidopteristes de France, Paris, 36–52.

[B7] Brown FM (1975) An annotated entomological bibliography of Romualdo Ferreira d'Almeida (1891–1969). Journal of the Lepidopterists' Society 29(1): 40–51.

[B8] Brown KS Freitas AVL (2000) Diversidade de Lepidoptera em Santa Teresa, Espírito Santo. Boletim do Museu de Biologia Mello Leitão 11/12: 71–118.

[B9] Bryk VF (1953) Lepidoptera aus dem Amazonasgebiete und aus Peru gesammelt von Dr. Douglas Melin und Dr. Abraham Roman. Arkiv för Zoologi 5(1): 1–269.

[B10] Butler AG (1867) A monograph of the genus *Euptychia*, a numerous race of butterflies belonging to the family Satyridae; with descriptions of sixty species new to science, and notes to their affinities, etc. Proceedings of the Zoological Society of London 1866: 458–504.

[B11] Butler AG (1877) On new species of the genus *Euptychia*, with a tabular view of those hitherto recorded. Journal of the Linnean Society of London (Zoology) 13(67): 116–128. 10.1111/j.1096-3642.1877.tb02375.x

[B12] Cock MJW (2014) An updated and annoted checklist of the larger butterflies (Papilionoidea) of Trinidad, West Indies: Papilionidae, Pieridae and Nymphalidae. Insecta Mundi 0353: 1–14.

[B13] Cong Q Grishin NV (2014) A new *Hermeuptychia* (Lepidoptera, Nymphalidae, Satyrinae) is sympatric and synchronic with *H.sosybius* in southeast US coastal plains, while another new *Hermeuptychia* species— not *hermes*—inhabits south Texas and northeast Mexico. ZooKeys 379: 43–91. 10.3897/zookeys.379.6394 PMC3935228 24574857

[B14] Cramer P (1775–1780) De uitlandische Kapellen voorkomende in de drie Waereld-Deelen Asia, Africa en America. Papillons exotiques des trois parties du monde l'Asie, l'Afrique et l'Amérique. Amsteldam, S. J. Baalde; Utrecht, Barthelemy Wild and J. Van Schoonhoven & Comp. 1(1/7): i–xxx, 1–16, 1–132, pls 1–84 ([31 December] 1775), (8): 133–155, pls 85–96 (1776); 2 (9/16): 1–151, pls 97–192 (1777); 3 (17/22): 1–128, pls 193–264 (1779), (23/24): 129–176, pls 265–288 (1780); 4 (25/26): 1–28, pls 289–304 (1780).

[B15] D’Abrera BL (1988) Butterflies of the Neotropical Region. Part V. Nymphalidae (Conc.) and Satyridae. Hill House, Black Rock, Victoria, 679–877.

[B16] D’Almeida R (1922) Mélanges lépidoptérologiques. Études sur les lépidoptères du Brésil. Berlin, R. Friedländer & Sohn, 226 pp.

[B17] D'Almeida RF (1937) Excursão scientifica aos rios Cuminá e Trombetas. Memórias do Instituto Oswaldo Cruz 32(2): 235–298. 10.1590/S0074-02761937000200008

[B18] de la Maza RG de la Maza J (1993) Mariposas de Chiapas. Gobierno del Estado de Chiapas, México, 224 pp.

[B19] DeVries PJ (1987) The butterflies of Costa Rica and their natural history. Papilionidae, Pieridae, Nymphalidae. Princeton University Press, Princeton, 327 pp.

[B20] Dyar HG (1914) Report on the Lepidoptera of the Smithsonian Biological Survey of the Panama Canal Zone. Proceedings of the United States National Museum 47(2050): 139–350. 10.5479/si.00963801.47-2050.139

[B21] Emmel TC Austin GT (1990) The tropical rain forest butterfly fauna of Rondonia, Brazil: species diversity and conservation. Tropical Lepidoptera 1(1): 1–12.

[B22] Espeland M Breinholt J Willmott KR Warren AD Vila R Toussaint EF Maunsell SC Aduse-Poku K Talavera G Eastwood R Jarzyna MA Guralnick R Lohman DJ Pierce NE Kawahara AY (2018) A comprehensive and dated phylogenomic analysis of butterflies. Current Biology 28(5): 770–778. 10.1016/j.cub.2018.01.061 29456146

[B23] Espeland M Breinholt J Barbosa EP Casagrande M Huertas B Lamas G Marín MA Mielke OHH Miller JY Nakahara S Tan D Warren AD Zacca T Kawahara AY Freitas AVL Willmott KR (2019) Four hundred shades of brown: higher level phylogeny of the problematic Euptychiina (Lepidoptera, Nymphalidae, Satyrinae) based on hybrid enrichment data. Molecular Phylogenetics and Evolution 131: 116–124. 10.1016/j.ympev.2018.10.039 30423438

[B24] Fabricius JC (1776) Genera insectorvm eorvmqve characteres natvrales secvndvm nvmervm, figvram, sitvm et proportionem omnivm partivm oris adiecta mantissa speciervm nvper detectarvm. Chilonii, Mich. Friedr. Bartsch, 310 pp.

[B25] Fabricius JC (1781) Species insectorvm exhibentes eorvm differentias specificas, synonyma avctorvm, loca natalia, metamorphosin adiectis observationibvs, *descriptionibvs*. Carl Ernest Bohn, Hamburgii et Kilonii 2: 1–494.

[B26] Fabricius JC (1787) Mantissa insectorvm sistens species nvper detectas adiectis synonymis, observationibvs, descriptionibvs, emendationibvs. Christian Gottlieb Proft, Hafniae, 2, 382 pp.

[B27] Fabricius JC (1793) Entomologia systematica emendata et aucta. Secundum classes, ordines, genera, species adjectis synonimis, locis, observationibus, descriptionibus. Christian Gottlieb Proft, Fil. et Soc., Hafniae, 3 (1), 488 pp.

[B28] Forster W (1964) Beitrage zur Kenntnis der Insektenfauna Boliviens XIX Lepidoptera III. Satyridae. Veröffentlichungen der zoologischen, Staatssammlung München 8: 51–188.

[B29] Francini RB Duarte M Mielke OHH Caldas A Freitas AVL (2011) Butterflies (Papilionoidea and Hesperioidea) of the "Baixada Santista" region, coastal São Paulo, southeastern Brazil. Revista Brasileira de Entomologia 55(1): 55–68. 10.1590/S0085-56262011000100010

[B30] Freitas AVL Brown KS Mielke OHH Santos JP Vasconcellos-Neto J (2016) Borboletas da Reserva Natural Vale, Linhares/ES. In: Rolim SG Menezes LFT Srbek-Araujo AC (Eds) Floresta atlântica de tabuleiro: diversidade e endemismos na Reserva Natural Vale.Belo Horizonte, Editora Rupestre, 317–328.

[B31] Fujisawa T Barraclough TG (2013) Delimiting species using single-locus data and the Generalized Mixed Yule Coalescent approach: a revised method and evaluation on simulated data sets. Systematic Biology 62(5): 707–724. 10.1093/sysbio/syt033 23681854 PMC3739884

[B32] Gaede M (1931) Familia Satyridae. Lepidopterorum Catalogus, 1–320 [Vol. 43]; 321–544 [Vol. 46]; 545–759 [Vol. 48].

[B33] Godart JB (1824) In: Latreille PA Godart JB (Eds) Encyclopédie Méthodique.Histoire naturelle. Entomologie, ou histoire naturelle des crustacés, des arachnides et des insectes. Veuve Agasse (Paris) 9(2), 329–828.

[B34] Godman FD Salvin O (1880–1881) Biologia Centrali-Americana. Insecta LepidopteraRhopalocera. Dulau & Co., Bernard Quaritch (London) 1(1): 1–32, pls 1, 2 [September 1879], 1(2) [November 1879]: 33–56; 1(3)[February 1880]: 57–72; 1(6)[August 1880]: 73–88; 1(9)[February 1881]: 89–96.

[B35] Hall A (1939) Catalogue of the LepidopteraRhopalocera (butterflies) of British Guiana. Agricultural Journal of British Guiana 10(1): 25–41.

[B36] Hancock EG (2015) The shaping role of Johan Christian Fabricius (1745–1808): William Hunter’s insect collection and entomology in eighteenth century London. In: Hancock EG Pearce N Campbell M (Eds) William Hunter’s World: the Art and Science of Eighteenth Century Collecting.Routledge, Abingdon, 151–163.

[B37] Hoang DT Chernomor O von Haeseler A Minh BQ Vinh LS (2017) UFBoot2: improving the ultrafast bootstrap approximation. Molecular Biology and Evolution 35(2): 518–522. 10.1093/molbev/msx281 PMC5850222 29077904

[B38] ICZN (1999) International Code of Zoological Nomenclature. Fourth edition. International Trust for Zoological Nomenclature, London. http://www.iczn.org/iczn/index.jsp.

[B39] Janzen DH Hallwachs W (2015) Dynamic Database for an Inventory of the Macrocaterpillar Fauna, and its Food Plants and Parasitoids, of Área de Conservación Guanacaste (ACG), Northwestern Costa Rica. http://janzen.sas.upenn.edu [2018-4-1]

[B40] Kaye WJ (1904) A catalogue of the LepidopteraRhopalocera of Trinidad. Transactions of the Entomological Society of London 1904(2): 159–224. 10.1111/j.1365-2311.1904.tb02744.x

[B41] Kalyaanamoorthy S Minh BQ Wong TKF Haeseler A von Jermiin LS (2017) ModelFinder: Fast model selection for accurate phylogenetic estimates. Nature Methods 14: 587–589. 10.1038/nmeth.4285 28481363 PMC5453245

[B42] Kirby WF (1871) A Synonymic Catalogue of Diurnal Lepidoptera. John Van Voorst, London, 690 pp.

[B43] Lamas G (2004) Nymphalidae. Satyrinae. Tribe Satyrini. Subtribe Euptychiina. In: Lamas G (Ed.) Checklist: part 4A.Hesperioidea – Papilionoidea. In: Heppner JB (Ed.) Atlas of Neotropical Lepidoptera. Volume 5A. Association for Tropical Lepidoptera/Scientific Publishers, Gainesville, 217–223.

[B44] Lamas G Grados J (1996) Mariposas de la Cordillera del Sira, Peru. Revista Peruana de Entomologia 39: 55–61.

[B45] Lamas G Robbins RK Harvey DJ (1991) A preliminary survey of the butterfly fauna of Pakitza, Parque Nacional del Manu, Peru, with an estimate of its species richness. Publicaciones del Museo de Historia Natural UNMSM (Lima), (A) 40: 1–19.

[B46] Lamas G Robbins RK Harvey DJ (1996) Mariposas del alto Río Napo, Loreto, Peru. Revista Peruana de Entomologia 39: 63–74.

[B47] Marín MA Uribe SI (2009) Actualización sobre Euptychiina (Lepidoptera: Satyrinae) representadas en la colección del Museo Entomológico Francisco Luis Gallego. Boletín del Museo Entomológico Francisco Luis Gallego 1(2): 22–32.

[B48] Mielke OHH Casagrande MM (1991) Lepidoptera: Papilionoidea e Hesperioidea coletados na Ilha de Maracá, Alto Alegre, Roraima, parte do projeto Maracá, com uma lista complementar de Hesperiidae de Roraima. Acta Amazonica 21: 175–210. 10.1590/1809-43921991211210

[B49] Miller LD (1968) The higher classification, phylogeny and zoogeography of the Satyridae (Lepidoptera). Memoirs of the American Entomological Society, Vol. 24, 174 pp.

[B50] Möschler HB (1877) Beiträge zur Schmetterlings-Fauna von Surinam. Verhandlungen der kaiserlich-königlichen zoologisch-botanischen Gesellschaft in Wien 26(1): 293–352.

[B51] Nakahara S Janzen DH Hallwachs W Espeland M (2015) Description of a new genus for *Euptychiahilara* (Lepidoptera, Nymphalidae, Satyrinae). Zootaxa 4012(3): 525–541. 10.11646/zootaxa.4012.3.7 26623873

[B52] Nakahara S Willmott KR Mielke OHH Schwartz J Zacca T Espeland M Lamas G (2018a) Seven new taxa from the butterfly subtribe Euptychiina (Lepidoptera: Nymphalidae: Satyrinae) with revisional notes on *Harjesia* Forster, 1964 and *Pseudeuptychia* Forster, 1964. Insecta Mundi 0639: 1–38.

[B53] Nakahara S Zacca T Huertas B Neild AFE Hall JPW Lamas G Holian LA Espeland M Willmott KR (2018b) Remarkable sexual dimorphism, rarity and cryptic species: a revision of the ‘*aegrota* species group’ of the Neotropical genus *Caeruleuptychia* Forster, 1964 with the description of three new species (Lepidoptera, Nymphalidae, Satyrinae). Insect Systematics and Evolution 49(2): 130–182. 10.1163/1876312X-00002167

[B54] Neild AFE (1996) The Butterflies of Venezuela. Part 1: Nymphalidae I (Limenitidinae, Apaturinae, Charaxinae). A Comprehensive Guide to the Identification of Adult Nymphalidae, Papilionidae, and Pieridae. Meridian Publications, London, 144 pp.

[B55] Paluch M Mielke OHH Nobre CEB Casagrande MM Melo DHA Freitas AVL (2011) Butterflies (Lepidoptera: Papilionoidea and Hesperioidea) of the Parque Ecológico João Vasconcelos Sobrinho, Caruaru, Pernambuco, Brazil. Biota Neotropica 11(4): 230–238.

[B56] Paradis E (2013) Molecular dating of phylogenies by likelihood methods: a comparison of models and a new information criterion. Molecular Phylogenetics and Evolution 67(2): 436–444. 10.1016/j.ympev.2013.02.008 23454091

[B57] Paradis E Claude J Strimmer K (2004) APE: analyses of phylogenetics and evolution in R language. Bioinformatics 20: 289–290. 10.1093/bioinformatics/btg412 14734327

[B58] Peña C Nylin S Freitas AVL Wahlberg N (2010) Biogeographic history of the butterfly subtribe Euptychiina (Lepidoptera, Nymphalidae, Satyrinae). Zoologica Scripta 39(3): 243–258. 10.1111/j.1463-6409.2010.00421.x

[B59] Pons J Barraclough TG Gomez-Zurita J Cardoso A Duran DP Hazel S Kamoun S Sumlin WD Vogler AP (2006) Sequence-based species delimitation for the DNA taxonomy of undescribed insects. Systematic Biology 55(4): 595–609. 10.1080/10635150600852011 16967577

[B60] Popescu AA Huber KT Paradis E (2012) ape 3.0: new tools for distance based phylogenetics and evolutionary analysis in R. Bioinformatics 28: 1536–1537. 10.1093/bioinformatics/bts184 22495750

[B61] Puillandre N Lambert A Brouillet S Achaz G (2012) ABGD, Automatic Barcode Gap Discovery for primary species delimitation. Molecular Ecology 21(8): 1864–1877. 10.1111/j.1365-294X.2011.05239.x 21883587

[B62] Ramos FA (1996) Nymphalid butterfly communities in an amazonian forest fragment. Journal of Research on the Lepidoptera 35: 29–41.

[B63] Riley ND Gabriel AG (1924) Catalogue of the Type Specimens of LepidopteraRhopalocera in the British Museum. Part I. Satyridae. Oxford University Press, London, 62 pp 10.5962/bhl.title.118835

[B64] Salinas JL Luis MA Llorente JE (2004) Papilionoidea of the evergreen tropical forests of Mexico. Journal of the Lepidopterists' Society 58(3): 125–142.

[B65] Sharpe EMB (1890) On a collection of Lepidoptera made by Mr. Edmund Reynolds on the rivers Tocantins and Araguaya and in the province of Goyaz, Brazil. Proceedings of the Zoological Society of London 1890(3): 552–577.

[B66] Singer MC Ehrlich PR (1993) Host specialization of satyrine butterflies, and their responses to habitat fragmentation in Trinidad. Journal of Research on the Lepidoptera 30(3/4): 248–256.

[B67] Tuxen SL (1967) The entomologist, J. C. Fabricius. Annual Review of Entomology 12: 1–15. 10.1146/annurev.en.12.010167.000245

[B68] Warren AD Davis KJ Stangeland EM Pelham JP Grishin NV (2018) Illustrated Lists of American Butterflies. http://www.butterfliesofamerica.com [2018-11-1]

[B69] Weymer G (1911) 4. Familie: Satyridae. In: Seitz A (Ed.) Die Gross-Schmetterlinge der Erde.A.Kernen (Stuttgart) 5: 173–192.

[B70] Whittaker PL (1983) Notes on the satyrid butterfly populations of Corcovado National Park, Costa Rica. Journal of the Lepidopterists' Society 37(2): 106–114.

[B71] Zhang J Kapli P Pavlidis P Stamatakis A (2013) A general species delimitation method with applications to phylogenetic placements. Bioinformatics 29(22): 2869–2876. 10.1093/bioinformatics/btt499 23990417 PMC3810850

