# Supplementary figures and images for: A revision of the new genus Amiga Nakahara, Willmott & Espeland, gen. n., described for Papilioarnaca Fabricius, 1776 (Lepidoptera, Nymphalidae, Satyrinae)
Source: Zookeys. 2019 Jan 31;(821):85–152. doi: 10.3897/zookeys.821.31782 (PMC6367311; doi:10.3897/zookeys.821.31782)

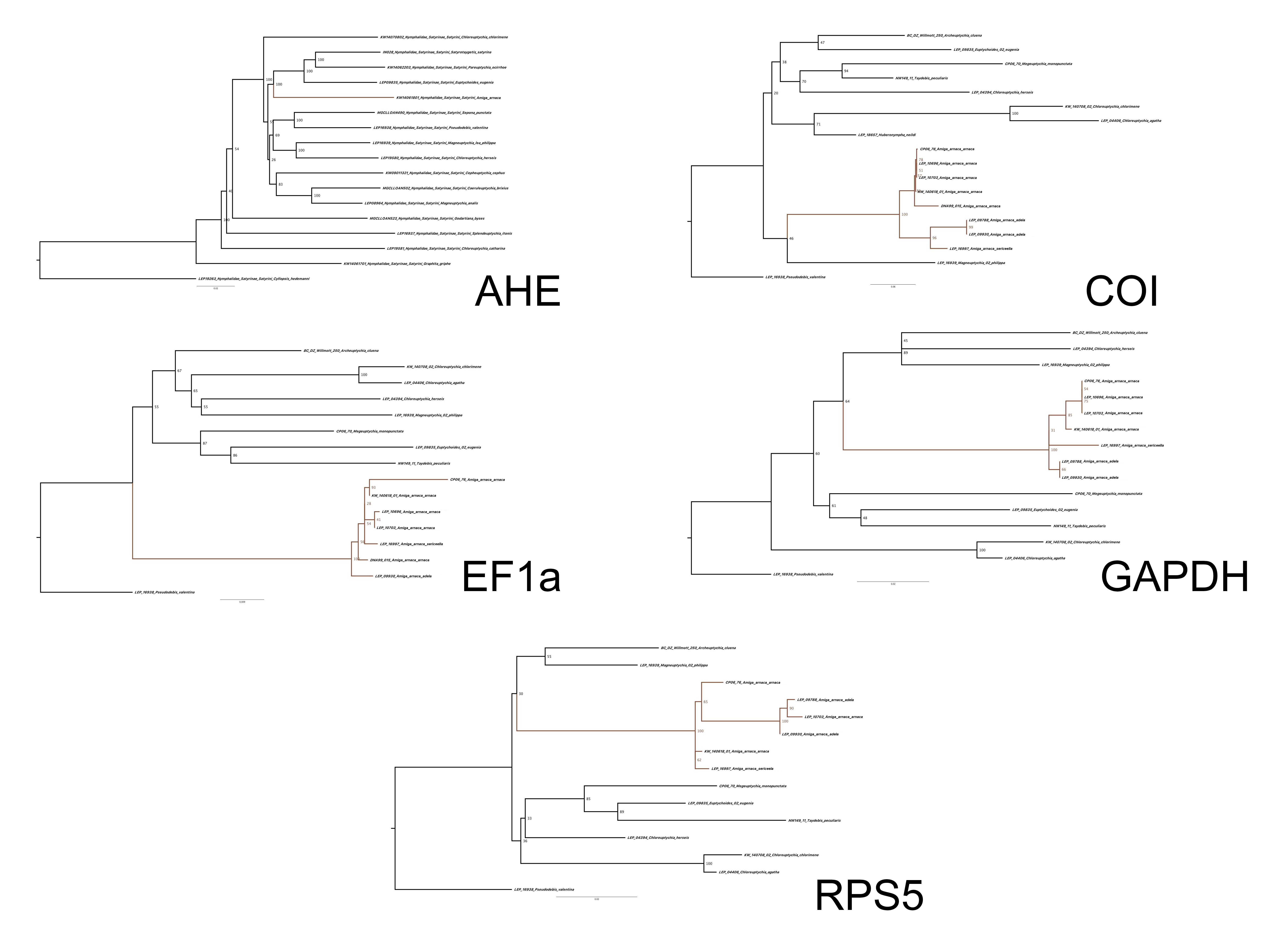

Supplement: Supplementary material 1 [file zookeys-821-085-s001.jpg]
